# Modularity and heterochrony in the evolution of the ceratopsian dinosaur frill

**DOI:** 10.1002/ece3.6361

**Published:** 2020-05-22

**Authors:** Albert Prieto‐Márquez, Joan Garcia‐Porta, Shantanu H. Joshi, Mark A. Norell, Peter J. Makovicky

**Affiliations:** ^1^ Institut Català de Paleontologia Miquel Crusafont Universitat Autònoma de Barcelona Barcelona Spain; ^2^ Integrative Research Center Field Museum of Natural History Chicago IL USA; ^3^ CREAF Barcelona Spain; ^4^ Department of Biology Washington University St. Louis MO USA; ^5^ Department of Neurology and Ahmanson Lovelace Brain Mapping Center University of California, Los Angeles Los Angeles CA USA; ^6^ Division of Paleontology American Museum of Natural History New York NY USA; ^7^ Department of Earth and Environmental Sciences University of Minnesota Minneapolis MN USA

**Keywords:** dinosaur, evolution, heterochrony, modularity, morphometrics

## Abstract

The fossil record provides compelling examples of heterochrony at macroevolutionary scales such as the peramorphic giant antlers of the Irish elk. Heterochrony has also been invoked in the evolution of the distinctive cranial frill of ceratopsian dinosaurs such as *Triceratops*. Although ceratopsian frills vary in size, shape, and ornamentation, quantitative analyses that would allow for testing hypotheses of heterochrony are lacking. Here, we use geometric morphometrics to examine frill shape variation across ceratopsian diversity and within four species preserving growth series. We then test whether the frill constitutes an evolvable module both across and within species, and compare growth trajectories of taxa with ontogenetic growth series to identify heterochronic processes. Evolution of the ceratopsian frill consisted primarily of progressive expansion of its caudal and caudolateral margins, with morphospace occupation following taxonomic groups. Although taphonomic distortion represents a complicating factor, our data support modularity both across and within species. Peramorphosis played an important role in frill evolution, with acceleration operating early in neoceratopsian evolution followed by progenesis in later diverging cornosaurian ceratopsians. Peramorphic evolution of the ceratopsian frill may have been facilitated by the decoupling of this structure from the jaw musculature, an inference that predicts an expansion of morphospace occupation and higher evolutionary rates among ceratopsids as indeed borne out by our data. However, denser sampling of the meager record of early‐diverging taxa is required to test this further.

## Graphical abstract

We find support for peramorphic evolution of the frill of ceratopsian dinosaurs, likely facilitated by the decoupling of this structure from jaw musculature.

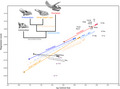

## 1 INTRODUCTION

The fossil record provides ample opportunities for the study of the role of heterochrony at macroevolutionary scales, due to the presence of taxa with larger body sizes and more extreme morphologies than those known in extant biota. For example, the Irish elk *Megaloceros gigantaeus*, with its giant antlers, is frequently cited as an iconic example of peramorphosis (Gould, 1974). Nonavian dinosaurs (hereafter dinosaurs), some of which reached extreme body sizes, and many of which exhibited bizarre anatomical traits, have also been investigated with respect to heterochronic evolution (Long & McNamara, 1995; McNamara & Long, 2012). Many previous studies on heterochrony in dinosaur evolution dealt with life‐history parameters related to size and growth (Erickson & Druckenmiller, 2011; Erickson et al., 2004; Lee & Werning, 2008; Weishampel & Horner, 1994), but a growing body of work has examined the role of heterochrony in the evolution of individual anatomical regions and character systems of dinosaurs (e.g., Bhullar et al., 2012; Farke, Chok, Herrero, Scolieri, & Werning, 2013; Forth, Hedrick, & Ezcurra, 2016; Guenther, 2009; Horner & Goodwin, 2006). Nevertheless, quantitative studies on heterochrony are still rare for this clade, despite a growing sample of species with increasing ontogenetic sampling and an improving understanding of their development.

**FIGURE 1:**
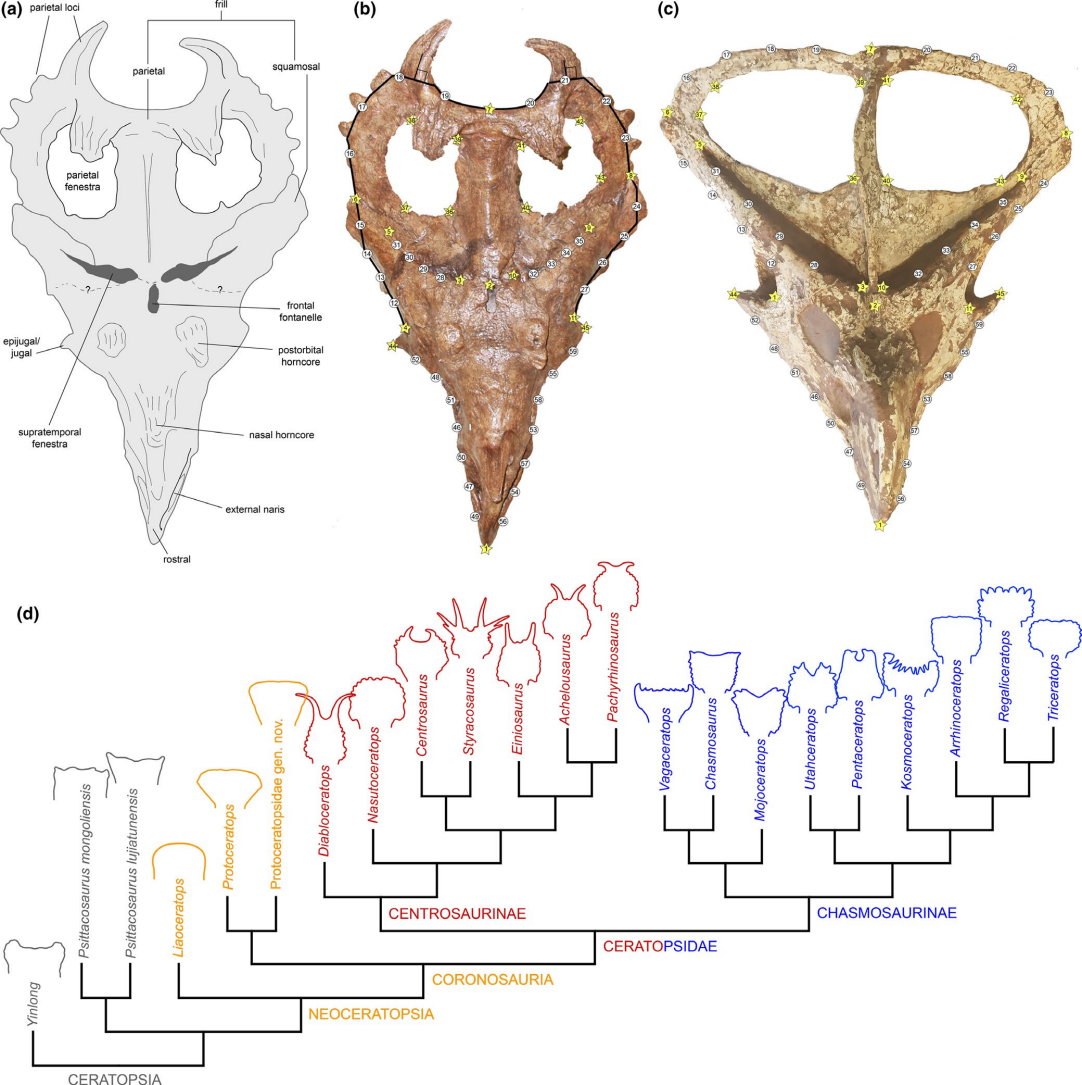
Landmark configuration used in the Procrustes superimposition shape analysis. (a) Line drawing of the dorsocaudal view of the skull of the ceratopsid *Centrosaurus apertus* (CMN 8795). (b) Dorsocaudal view of CMN 8795. (c) Dorsocaudal view of the skull of the basal neoceratopsian *Protoceratops andrewsi* (FMNH PR 14045). (d) Simplified phylogeny of Ceratopsia with the species included in this study (Table 1). The tree is based on those of Xu et al. (2006), Farke et al. (2014), Longrich (2014), Brown and Henderson (2015), and Evans and Ryan (2015). Yellow stars and white circles correspond to landmarks and semilandmarks, respectively. Landmark/semilandmark numbering is in the order in which they were digitized and indicate: 1, tip of the rostral bone; 2, rostral end of the parietal at the sagittal plane of the skull; 3 and 10, rostrolateral margin of the supratemporal fenestra; 4 and 11, rostral‐most margin of the infratemporal fenestra; 5 and 9, caudolateral end of the supratemporal fenestra; 6 and 8, squamosal–parietal contact at the caudolateral margin of the frill or skull; 7, mid‐point of the caudal margin of the frill at the interparietal contact; 36 and 40, rostromedial corner of the parietal fenestra; 37 and 43, rostrolateral corner of the parietal fenestra; 38 and 42, caudolateral corner of the parietal fenestra; 39 and 41, caudomedial corner of the parietal fenestra; and 44 and 45, lateroventral tip of the jugal boss

Here, we examine the evolution of the hallmark cranial frill (Figure 1) of one of the most iconic dinosaur lineages, the Ceratopsia. Ceratopsians were a diverse (>70 spp.) ornithischian clade and are one of the best‐sampled major clades of nonavian dinosaurs, with some species representing the most abundant taxa in their fossil deposits (Dodson, Forster, & Sampson, 2004; Dodson, 2013). Originating in the Middle to Late Jurassic (Xu, Forster, Clark, & Mo, 2006; You & Dodson, 2004; Zhao, Zhengwu, & Xu, 1999), these animals were among the more dominant herbivores of Cretaceous terrestrial ecosystems in North America (Dodson et al., 2004; Farke, Maxwell, Cifelli, & Wedel, 2014) and Asia (Makovicky & Norell, 2006; Maryanska & Osmolska, 1975; Xu et al., 2006; You, Li, Ji, Lamanna, & Dodson, 2005), and are also reported from the Late Cretaceous of Europe (Lindgren et al., 2007; Ösi, Butler, & Weishampel, 2010). Members of this clade had remarkably large heads (Sereno, Xijin, Brown, & Lin, 2007), laterally compressed turtle‐like beaks, and shearing dentitions (Ostrom, 1964), which are thought to have contributed to their evolutionary success (Ostrom, 1966). Most notably, ceratopsians are instantly recognizable for their exaggerated cranial structures (Knell & Sampson, 2011; Padian & Horner, 2011), particularly the prominent parieto‐squamosal frill at the back of the skull, characteristic of the clade as a whole, and also facial and frill ornaments in the more derived Campano‐Maastrichtian Ceratopsidae (Brown & Henderson, 2015; Dodson & Currie, 1990; Dodson et al., 2004; Sampson et al., 2010; Sampson, Lund, Loewen, Farke, & Clayton, 2013).

The ceratopsian frill is a neomorphic structure that overhangs the occiput to varying degrees and is formed by the parietal along the midline with contributions of the squamosals along its lateral edges (Dodson et al., 2004; Hatcher, Marsh, & Lull, 1907; Figure 1). In small bodied, early‐diverging ceratopsians it is relatively short and narrow, but in large bodied (>1,000 kg) ceratopsids, the frill alone can be over a meter in length and width and constitutes more than half the length of the skull (Dodson et al., 2004). It is perforated by a pair of fenestrae in most neoceratopsians. Although the frill margin is smooth or relatively unadorned in most early‐diverging species, the large and speciose ceratopsids bear epiparietal and episquamosal ossifications that form a spectacular diversity of structures projecting from the frill, and that distinguish individual species from one another (Figure 1).

Unsurprisingly, heterochronic processes have been invoked to explain this phylogenetic variation and its general relationship with the evolution of body size (~5 kg–10 tons), with various authors interpreting frill evolution as a peramorphic process (e.g., Long & McNamara, 1995, 1997; McNamara, 2012; Tumarkin & Dodson, 1998). These studies have only dealt with scant quantification of either frill size or shape, however, and are based on simple linear measurements or qualitative observations.

Here, we take a geometric morphometric approach to investigate patterns of morphological variation of the ceratopsian frill, bearing on three interrelated questions regarding the ontogeny and evolution of this structure:

1. Does shape variation vary randomly or are patterns (e.g., phylogenetic; functional) evident in frill morphospaces? Which aspects of frill structure exhibit the most variation across species and within species with good ontogenetic sampling?
2. Does the frill constitute a module in the ceratopsian skull across both evolutionary and developmental scales? Heterochronic evolution is thought to occur within semi‐independent collections of traits, or modules (Gerber & Hopkins, 2011; Goswami, 2007; Gould, 1977; Klingenberg & Marúgan‐Lobon, 2013; Olson & Rosell, 2006), so establishing whether the frill behaves as a module across evolutionary and ontogenetic scales is prerequisite to testing for heterochrony as a process for changes in frill shape.
3. Is frill evolution driven by heterochronic processes such as peramorphosis as proposed by previous authors (Long & McNamara, 1995, 1997; McNamara, 2012; Tumarkin & Dodson, 1998), and is it possible to characterize these processes as well as their potential drivers?

### 1.1 Institutional abbreviations

AMNH, American Museum of Natural History, New York, USA; BMRP, Burpee Museum of Natural History, Rockford, Illinois, USA; CMN, Canadian Museum of Nature, Ottawa, Canada; FMNH, Field Museum of Natural History, Chicago, USA; IVPP, Institute of Vertebrate Paleontology and Paleoanthropology, Beijing, China; MOR, Museum of the Rockies, Bozeman, Montana, USA; MPC, Mongolian Paleontological Collection, Ulaanbaatar, Mongolia; MDP, Mongolian Dinosaur Project; MPM, Milwaukee Public Museum, Milwaukee, Wisconsin, USA; ROM, Royal Ontario Museum, Toronto, Ontario, Canada; TMP, Royal Tyrrell Museum of Paleontology, Drumheller, Alberta, Canada; UCMP, University of California Museum of Paleontology, Berkeley, California, USA; UMNH, Natural History Museum of Utah, Salt Lake City, Utah, USA; YPM, Yale Peabody Museum of Natural History, New Haven, Connecticut, USA.

## 2 MATERIALS AND METHODS

### 2.1 Sampled taxa and specimens

Our study sample consisted of 25 species representing all taxa for which reliable data on frill shape are available and nearly half the known taxonomic diversity of Ceratopsia. The sampled species comprise three basal Ceratopsia, four basal Neoceratopsia, 10 chasmosaurine, and eight centrosaurine ceratopsids (Figure 1d and Table 1). We conducted two‐dimensional geometric morphometric analyses to quantify frill shape variation throughout ceratopsian evolution. Although skulls are three‐dimensional structures, the two‐dimensional analysis is an appropriate simplification in that it suffices to capture most of the shape of the ceratopsian frill. This is because the frill is a dorsoventrally compressed structure, and as such, its morphology is mostly contained and exposed on a dorsal plane of the skull that is facing anterodorsally (Figure 1). Furthermore, and from a practical point of view, 3D scanning was not an available option and it was not possible to photograph many of the specimens of our sample in all the required views necessary to construct a 3D photogrammetric model of the skull. In particular, it was not uncommon to encounter specimens in exhibit mounts obscuring some views (e.g., ventral or posterior) of the skull. For the analyses at interspecific level, each species was represented by one individual. As a proxy for adulthood, we selected a specimen reaching the largest (or at least within the range of the largest) known size for the species. For *Triceratops*, the select specimen (YPM 1823) exhibits anatomical criteria such as anterior postorbital horn orientation and flattened episquamosal and epiparietal ossifications identified as representative of adulthood by Horner and Goodwin (2006); these authors also regard all YPM specimens as adults (Horner and Goodwin (2006: p. 2757). With respect to *Protoceratops*, our representative skull in the all‐species sample (FMNH PR 14045) is larger than two skulls (AMNH 6418; AMNH 6466) that have been aged histologically to be 17 and 19 years and interpreted as adults in an unpublished study (Makovicky, Sadleir, Dodson, Erickson, & Norell, 2007). Our representative specimen for *P. lujitunensis* is of a size (skull length = 19 cm) that overlaps with the oldest sampled individuals in a histological study of that species (Erickson, Makovicky, Inouye, Keqin, & Zhou, 2009). Thus, for taxa with available skeletochronological or histological age data, our choice to use the largest available specimen is selecting adult or large subadult specimens for comparisons. We also selected those specimens that preserve a complete or nearly complete and undistorted frill on at least one side of the skull; taxa lacking sufficiently complete frills were excluded.

**TABLE 1:** Sample of ceratopsian specimens used as representatives of the main ceratopsian clades in the morphometric analyses

Species (Abbreviation)	Specimen
*Achelousaurus horneri* (Ach)	Centrosaurinae; Sampson, 1995, figure 5b, reconstruction based on MOR 845 and 591
*Anchiceratops ornatus* (Anc)	Chasmosaurinae; Mallon, Holmes, Eberth, Ryan, & Anderson, 2011, figure 2, AMNH 5251
*Arrhinoceratops brachyops* (Arr)	Chasmosaurinae; Mallon, Holmes, Anderson, Farke, & Evans, 2014, figure 4, ROM 796
*Centrosaurus apertus* (Cen)	Centrosaurinae; CMN 8795
*Chasmosaurus belli* (Cen)	Chasmosaurinae; CMN 2245
*Diabloceratops eatoni* (Dbl)	Centrosaurinae; UMNH cast of UMNH VP 16699
*Einiosaurus procurvicornis* (Ein)	Centrosaurinae; Sampson, 1995, figure 4b reconstruction based on MOR 456
*Kosmoceratops richardsoni* (Kos)	Chasmosaurinae; UMMH cast UMNH VP 17000
*Leptoceratops gracilis* (Lep)	Basal Neoceratopsia; Sternberg, 1951, Pl. XLVIII, CMN 8889
*Liaoceratops yanzigouensis* (Lia)	Basal Neoceratopsia; IVPP V12738
*Mojoceratops perifania* (Moj)	Chasmosaurinae; Longrich, 2010, figure 9, including TMP 1983.25.1
*Nasutoceratops titusi* (Nas)	Centrosaurinae; UMNH cast of UMNH VP 16800
*Pachyrhinosaurus lakustai* (Pac)	Centrosaurinae; UMNH cast of TMP 1986.55.258
*Pentaceratops sternbergii* (Pen)	Chasmosaurinae; Forster, Sereno, Evans, & Rowe, 1993, figure 6, AMNH 6325 & MNA Pl.1747
*Protoceratops andrewsi* (Prt)	Basal Neoceratopsia; FMNH PR 14045
Ukhaa Tolgod protoceratopsid gen. et sp. nov. (Ukt)	Basal Neoceratopsia; IGM 100/1246 (Ukhaa Tolgod, Mongolia)
*Psittacosaurus lujiatunensis* (Psm)	Basal Ceratopsia; FMNH PR 2588 (cast)
*Psittacosaurus mongoliensis* (Psl)	Basal Ceratopsia; AMNH 6254
*Regaliceratops peterhewsi* (Reg)	Chasmosaurinae; TMP 2005.055.0001
*Styracosaurus albertensis* (Sty)	Centrosaurinae; CMN 344
*Triceratops horridus* (Tri)	Chasmosaurinae; YPM 1823 (Hatcher et al., 1907, Pl. XXVIII)
*Utahceratops gettyi* (Uta)	Chasmosaurinae; Sampson et al., 2010, figure 4, including UMNH VP 16784
*Vagaceratops irvinensis* (Vag)	Chasmosaurinae; Holmes, Forster, Ryan, & Shepherd, 2001, figure 3, CMN 41357
*Wendiceratops pinhornensis* (Wen)	Centrosaurinae; Evans & Ryan, 2015, figure 15, including TMP 2011.051.0009
*Yinlong downsi* (Yin)	Basal Ceratopsia; IVPP V14530

Note

**FIGURE 2:**
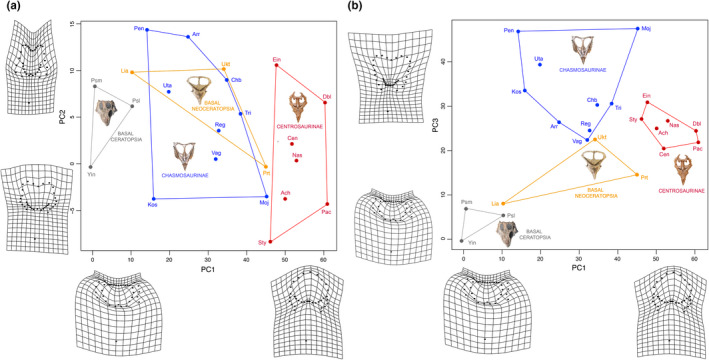
Results the morphometric analyses of the ceratopsian sample, excluding the parietal fenestra to allow inclusion of basal ceratopsians and *Triceratops*. (a) PCA bivariate plot summarizing the results of the Procrustes superimposition analysis for PC 1 and PC 2. (b) PCA bivariate plot for PC 1 and PC 3 of same

**FIGURE 3:**
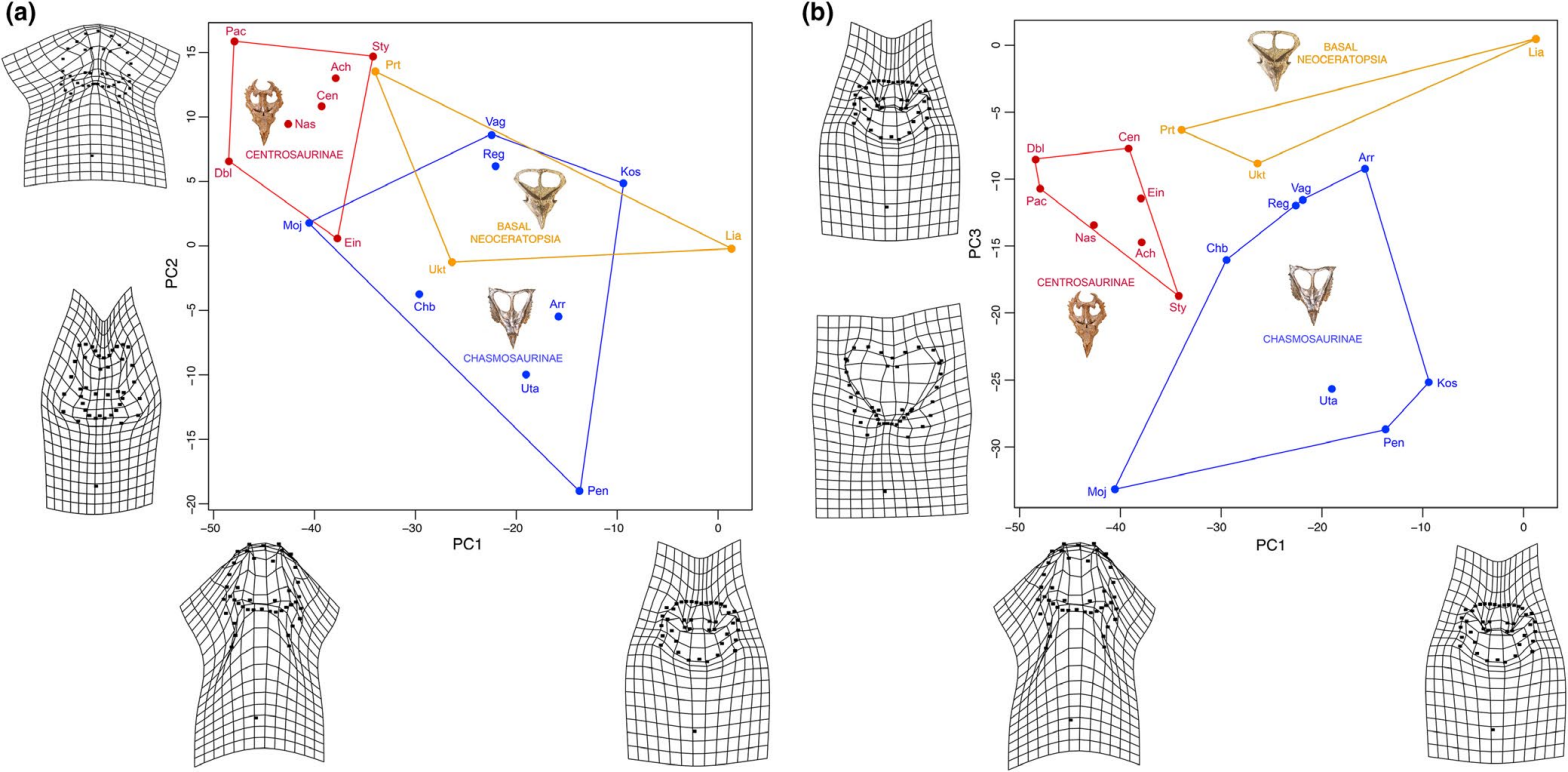
Results of the Procrustes superimposition analysis of neoceratopsians possessing a parietal fenestra. (a) PCA bivariate plot summarizing the results of the analysis for PC 1 and PC 2. (b) PCA bivariate plot for PC1 and PC3

**FIGURE 4:**
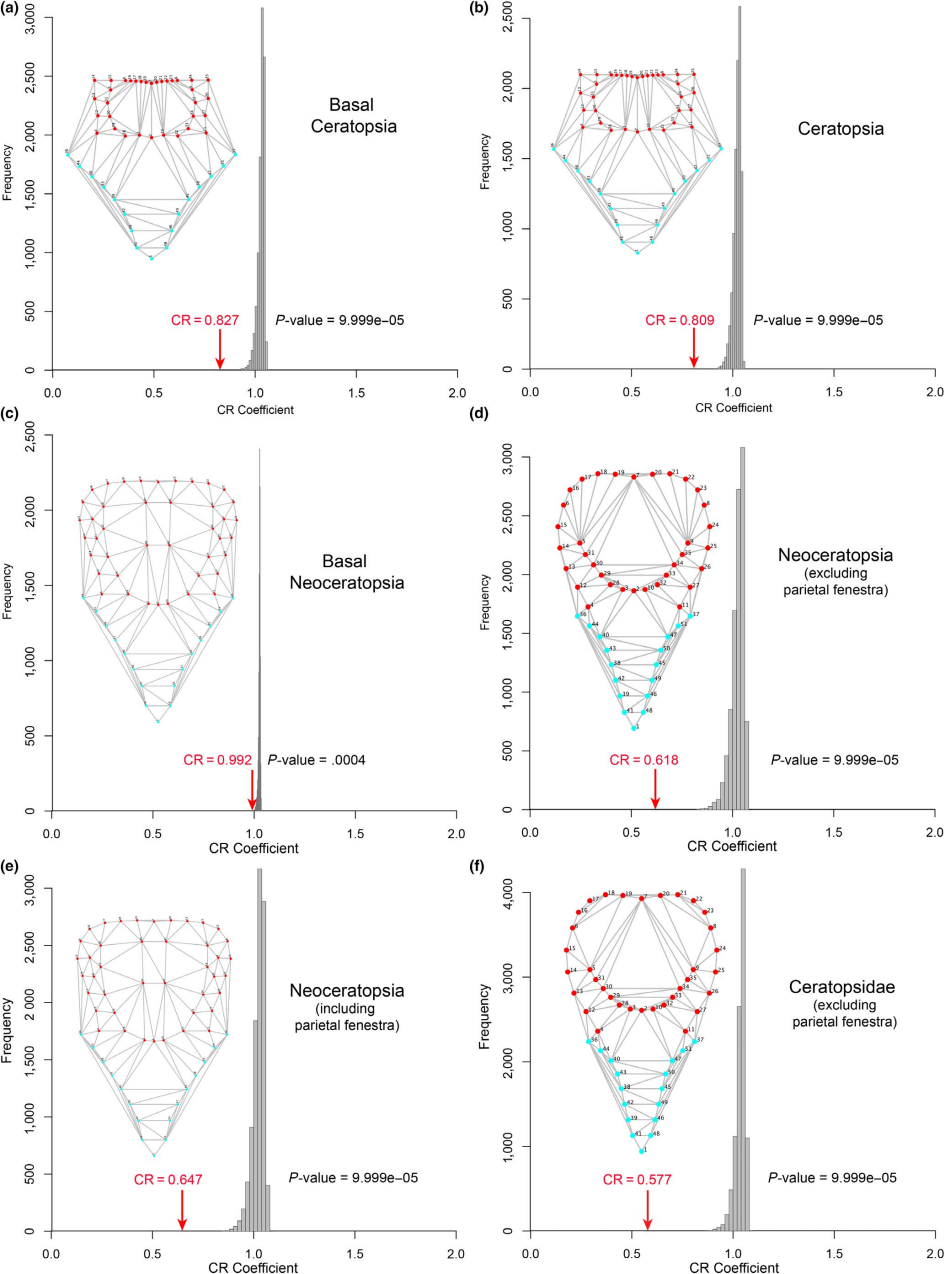
Distribution of covariance ratios (CR) for all the evaluated 10,000 random subsets of landmarks for various paraphyletic and monophyletic ceratopsian groups. The red arrow indicates the CR for the hypothesis of modularity of the frill. The frame diagrams display the landmarks digitized on the dorsal view of the skull; red landmarks correspond to those of the hypothesized frill module

**FIGURE 5:**
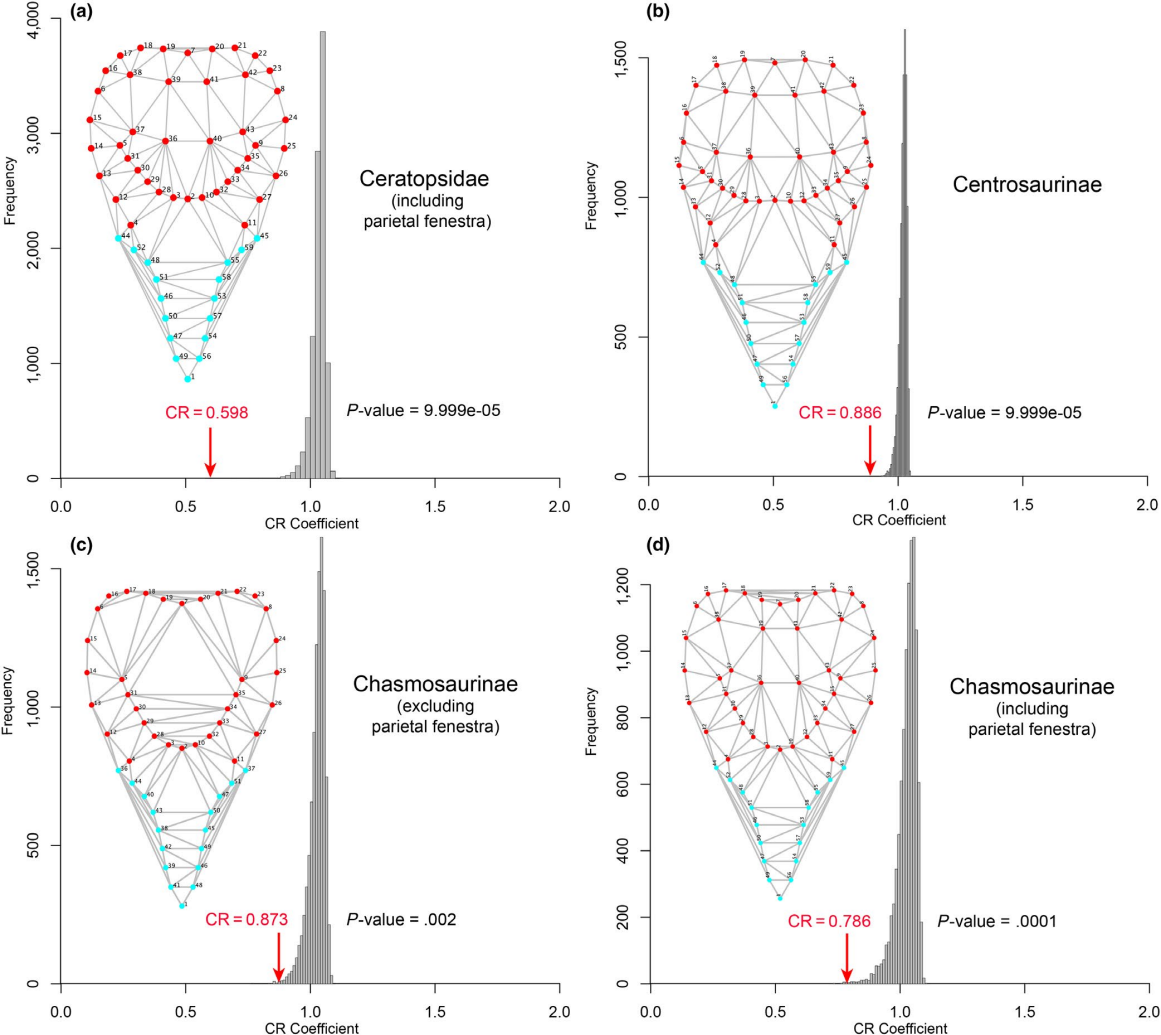
Distribution of covariance ratios (CR) for all the evaluated 10,000 random subsets of landmarks for various paraphyletic and monophyletic ceratopsian groups. The red arrow indicates the CR for the hypothesis of modularity of the frill. The frame diagrams display the landmarks digitized on the dorsal view of the skull; red landmarks correspond to those of the hypothesized frill module

**FIGURE 6:**
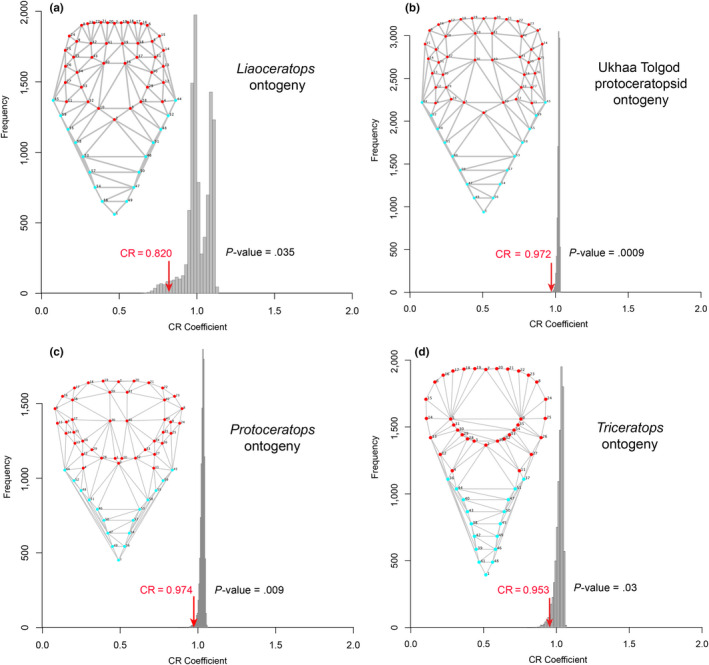
Distribution of covariance ratios (CR) for all the evaluated 10,000 random subsets of landmarks for growth series of basal neoceratopsians *Liaoceratops,* a new protoceratopsid taxon, *Protoceratops* and the chasmosaurine ceratopsid *Triceratops*. The red arrow indicates the CR for the hypothesis of modularity of the frill. The frame diagrams display the landmarks digitized on the dorsal view of the skull; red landmarks correspond to those of the hypothesized frill module

**FIGURE 7:**
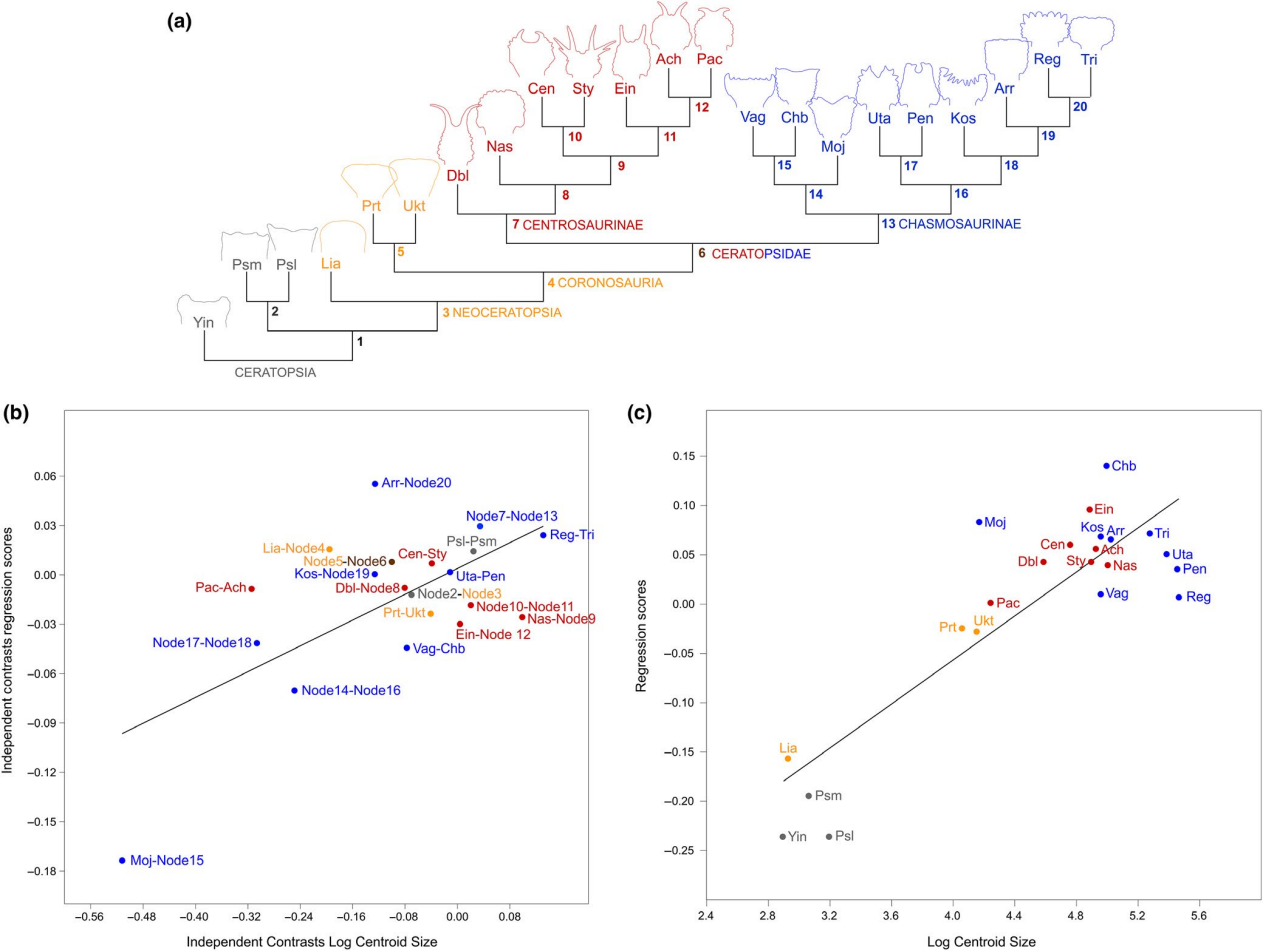
Regression analysis of ontogenetic trajectories from four neoceratopsian species against log‐transformed centroid size using the regression score as shape variable. (a) Simplified phylogeny of Ceratopsia with the species included in the regression analyses, based on the trees in Xu et al. (2006), Farke et al. (2014), Longrich (2014), Brown and Henderson (2015), and Evans and Ryan (2015); labeled nodes correspond to those appearing in b. (b) Regression analysis using phylogenetic contrasts. (c) Nonphylogenetically corrected regression analysis. Species abbreviations are as in Table 1

The asterisk denotes those specimens used only in the SRVF analysis. The abbreviations noted between brackets correspond to those used in Figures 2, 3, 4, 5, 6, 7.

The skulls of the sampled species were photographed in dorsocaudal view, orthogonal to the dorsal surface of the frill. Particular attention was paid to maintaining that orientation as strictly as possible, given that deviations from a dorsocaudal view can distort the contour of the frills and, potentially, the results of the morphometric analyses. In the case of specimens preserving only one side of the frill and/or displaying substantial taphonomic distortion, we symmetrically mirrored the better‐preserved side around the sagittal plane of the skull. This correction was applied to one *Liaoceratops yanzigouensis* specimen (IVPP V12738), four *P. andrewsi* specimens (AMNH 6425, 6433 and 6439; MPC D100/502), and the ceratopsids *Regaliceratops peterhewsi* (TMP 2005.055.0001) and *Pachyrhinosaurus lakustai* (cast of TMP 1986.55.258). For the analyses across species, we sampled only one specimen per species in order to prevent biasing the mean shape of the sample by including unequal numbers of specimens per taxon (a common situation in the dinosaurian fossil record).

For ease of interpretation of morphospace plots, we color coded the sampled taxa in four groupings: basal ceratopsians comprising non‐neoceratopsian taxa with short, incipiently developed and unfenestrated frills (gray); basal noeceratopsians, representing the nonceratopsid neoceratopsian part of our sample with larger frills with unornamented margins (orange); and the two main ceratopsid lineages Chasmosaurinae (blue) and Centrosaurinae (red), both of which have heavily ornamented frills. Although the primary function of these designations is to facilitate the interpretation of morphospace plots, they correspond approximately to previously proposed categories of frill evolution (e.g., Ostrom, 1966).

### 2.2 Shape analysis using procrustes superimposition

The ceratopsian frill and skull table were digitized using 21 homologous landmarks (Figure 1b,c). Preservational issues and fusion of cranial bones prevented the identification of additional landmarks of the skull roof and lateral portions of the facial skeleton exposed in dorsocaudal view. We added 38 evenly spaced semilandmarks (Figure 1b,c) to more completely capture the shape of the frill and skull. In most neoceratopsians, the frill is perforated by a pair of parietal fenestrae, which were digitized using four landmarks and included in our analysis. However, the parietal fenestra is absent in non‐neoceratopsian Ceratopsia, *Leptoceratops,* hatchling specimens of *Protoceratops*, and in all but the oldest individuals of *Triceratops* (Scannella & Horner, 2010; *Torosaurus* of some authors). In order to include these taxa in our study, we also used an iteration of the Procrustes superimposition analysis in which the parietal fenestra was excluded from the landmark configuration.

Within Neoceratopsia, the more derived Ceratopsidae exhibit hypertrophied frill ornamentation, often in the form of spikes, hooks, and other types of processes of greatly varying morphologies (e.g., Figure 1a). Notable examples include the long parietal spikes of *Styracosaurus albertensis* (Ryan, Holmes, & Russell, 2007) and *Diabloceratops eatoni* (Kirkland & DeBlieux, 2010). We excluded such ornamental structures from the landmark configuration (Figure 1b) in order to compare the morphology of the landmark configuration of the margins of the ceratopsid frills to that of unornamented ceratopsid outgroups, and also because the homologies between the epiparietal and episquamosal ornaments are still debated (e.g., Brown & Henderson, 2015; Farke et al., 2011; Mallon, Ott, Larson, Iuliano, & Evans, 2016). Exclusion of the ceratopsid ornamentation was accomplished by linking the bases of all ornamental epiparietal and episquamosal structures with straight lines orthogonal to their long axes, resulting in a polygon following the boundary of the frills (Figure 1b). Landmarks and semilandmarks were digitized on photographs of the ceratopsian skulls using the program tpsUtil version 1.68 (Rohlf, 2016a). Both sides of the skull were digitized to better allow for tests of modularity. The files with the landmark coordinates (Appendices [Link], [Link], [Link], [Link], [Link], [Link]) were created in tpsDig2 version 2.26 (Rohlf, 2016b).

The landmark configurations of the skull sample were superimposed by means of the generalized Procrustes procedure, which eliminates all nongeometric information after scaling, rotation, and translation relative to the grand mean (Bookstein, 1991; Klingenberg, Barluenga, & Meyer, 2002). Optimal superimposition of configurations of landmark coordinates used least‐squares estimation of translation, rotation, and other scaling parameters (Slice, 2007). The generalized Procrustes procedure was conducted using the function “gpagen” in the R package Geomorph 3.0.4 (Adams and Otarola‐Castillo, 2013).

The resulting shape variables were used to perform a phylogenetic principal components analysis (pPCA). This is a widely used dimension reduction technique that represents sample variation on a reduced number of axes, taking into account phylogenetic information (Revell, 2009). We performed the pPCA using a correlation structure derived from a Brownian motion model by means of the function “phyl.pca” in the R package Phytools 0.6‐0.0 (Revell, 2012). Because there is no published phylogenetic analysis that fully resolves the relationships of all known species of basal ceratopsians, centrosaurines, and chasmosaurines, we constructed an informal supertree (Figure 1d) that combined the topologies of Farke et al. (2014, resolving basal neoceratopsian relationships), Longrich (2014, focusing on chasmosaurines), and Evans and Ryan (2015, focusing on centrosaurines); the phylogeny of Brown and Henderson (2015) was used to position *Regaliceratops*. The tree was trimmed to only include the taxa in our GM sample and calibrated using the first and last appearance datum (Appendix S7) for each species via implementation of the R package “strap” (Bell & Lloyd, 2015). We used the function DatePhylo in the package strap to estimate branch lengths using the method of Brusatte, Benton, Ruta, and Lloyd (2008) for use in the pPCA and other comparative analyses.

Morphospace occupation by the different ceratopsian clades and grades was visualized by plotting the shapes under consideration on combinations of the first three pPCA axes. Shape variation contained in each plot was represented by estimating the mean landmark coordinates of all species and calculating the shape differences existing between the mean and each of the species lying at the extremes of each pPC axis (functions “mshape” and “plotRefToTarget” in the R package Geomorph 3.0.4 (Adams and Otaróla‐Castillo, 2013). Testing for significance in the morphological difference among the two grades and five clades of ceratopsians under consideration (i.e., basal ceratopsians, Neocertopsia, nonceratopsid neoceratopsians, Carnosauria, Ceratopsia, Centrosaurinae, and Chasmosaurinae) was accomplished by means of pairwise permutational ANOVAs along each of the first three pPC axes, which relied on 1,000 randomizations to compute p‐values (this was calculated with R custom scripts relying on the function “lm”; Tables 4 and 5).

### 2.3 Evaluation of the frill modularity hypothesis

Certain traits in organisms may become compartmentalized into modules, clusters of characters that evolve in a highly correlated manner within a cluster, but relatively independently from characters in other clusters (Klingenberg, 2008). In contrast to their strong internal integration, intermodular correlations are hypothesized to be weaker (Adams, 2016; Klingenberg, 2009; Klingenberg & Marúgan‐Lobon, 2013). Because the ceratopsian frill is a neomorphic structure among archosaurs and exhibits considerable variation in shape, size, and ornamentation among species that otherwise exhibit reltively minor differences in postcranial and dental characters, we hypothesize that it may have evolved in modular fashion, that is, relatively independent of the rest of the skull. Accordingly, we evaluated the a priori hypothesis of frill modularity by asking whether there is stronger covariation among the landmarks defining the frill than between any subset of them and subsets of landmarks from other skull partitions. We employed the covariance ratio (CR; Adams, 2016) as a measure of the covariation between subsets of landmarks. The CR measures the overall covariation between landmark partitions (or modules) relative to the overall covariation within partitions (Adams, 2016), resulting in a coefficient that ranges from zero to positive values. CR values ranging from zero to one are indicative of modularity, whereas values above one characterize greater covariation between partitions than within them (Adams, 2016). Although higher within‐partition covariation does not necessarily imply modularity, it is a testable prediction of the modularity hypothesis (Klingenberg, 2009). This reasoning also provides a basis for accepting the null hypothesis: If covariation between the frill partition and other partitions of the skull is not weaker than among most or all (e.g., >95%) of the other partitions considered, the hypothesis of frill modularity can be rejected. Notably, the CR coefficient is not affected by sample size nor the number of variables (Adams, 2016).

The CR analysis was implemented in the R package Geomorph (Adams and Otárola‐Castillo, 2013). The analysis compared the CR coefficient of the landmark partition corresponding to the frill with that of 10,000 random alternative subsets of landmarks across the whole skull. The output of this analysis was summarized as a histogram that displays the distribution of CR coefficients for all the randomized partitions in the skull, thus providing a means for statistically evaluating the robustness of the observed CR coefficient. We used the landmark configuration from the Procrustes analyses described above to test for the modularity of the frill at various evolutionary and ontogenetic levels. Two variations of the analyses, that is, including and excluding those landmarks corresponding to the parietal fenestra, were conducted for the clades Neoceratopsia, Ceratopsidae, and Chasmosaurinae to allow for inclusion of the chasmosaurine *Triceratops horridus*.

Intraspecifically, we assessed frill modularity in growth series of *Liaoceratops yanzigouensis*, *Protoceratops andrewsi*, a new undescribed protoceratopsid from Ukhaa Tolgod, and *Triceratops horridus*. Only five exemplars of *L. yanzigouensis* were available, but these spanned a substantial range of sizes (Table 2). *P. andrewsi* is known from numerous specimens, many of which are exceptionally preserved, including a nearly complete developmental series ranging from hatchlings through full‐grown adults (Brown & Schlaikjer, 1940; Dodson, 1976; Fastovsky et al., 2011; Hone, Wood, & Knell, 2016; Lull, 1908; Russell, 1935).The growth series of 21 *P. andrewsi* and the nine specimens of the new Ukhaa Tolgod protoceratopsid spanned much of the development of these species, with specimens ranging from a few dozens of mm to more than 300 mm in basal skull length (Table 2). *T. horridus* is the only available ceratopsid preserving an ontogenetic series of skulls with frills spanning much of its development (Goodwin, Clemens, Horner, & Padian, 2006; Horner & Goodwin, 2006, 2008), although the extensive MOR sample of *Triceratops* was not available to us for study. We followed Scannella and Horner (2010) in considering *Torosaurus* a junior synonym of *Triceratops* in our primary analysis. The *T. horridus* ontogenetic series was less complete, ranging from 450 mm to nearly 1,150 mm in basal skull length (*n* = 8, Table 3). The *T. horridus* specimens UCMP 154452 and MPM VP6841 were excluded from the modularity analyses, because they do not preserve the rostrum.

**TABLE 2:** Sample of basal neoceratopsian and protoceratopsid specimens used in this study

Species	Specimen	Basal skull length (mm)	Frill inclination	Relative frill length
*L. yanzigouensis*	Uncatalogued #1	66	—	—
*L. yanzigouensis*	IVPP V12633	93	—	—
*L. yanzigouensis*	IVPP V12738	99	—	—
*L yanzigouensis*	Uncatalogued #2	137	—	—
*L. yanzigouensis*	Uncatalogued #3	165	—	—
*P. andrewsi*	MPC‐D100/530	28	—	—
*P. andrewsi*	AMNH 6419	76	22°	0.61
*P. andrewsi*	AMNH 6431	150	22°	1.04
*P. andrewsi*	AMNH 6409	191	43°	1.02
*P. andrewsi*	AMNH 6444	210	27°	?
*P. andrewsi*	AMNH 6408	235	27°	0.65
*P. andrewsi*	MPC‐D100/502	250	31°	1.22
*P. andrewsi*	AMNH 6433	261	34°	0.69
*P. andrewsi*	AMNH 6429	269	33°	0.91
*P. andrewsi*	AMNH 6439	271	49°	0.73
*P. andrewsi*	MDP 530	297	?	1.06
*P. andrewsi*	AMNH 6425	313	43°	0.86
*P. andrewsi*	AMNH 6413	314	40°	0.73
*P. andrewsi*	FMNH PR 14045	330	37°	1.05
*P. andrewsi*	AMNH 6414	341	57°	0.67
*P. andrewsi*	AMNH 6466	357	36°	0.69
Ukhaa Tolgod protoceratopsid	IGM 100/3655	24	—	—
Ukhaa Tolgod protoceratopsid	IGM 100/1008	28	—	—
Ukhaa Tolgod protoceratopsid	IGM 100/10020	37	—	—
Ukhaa Tolgod protoceratopsid	IGM 100/1013	47	—	—
Ukhaa Tolgod protoceratopsid	IGM 100/1019	69	5°	—
Ukhaa Tolgod protoceratopsid	IGM 100/3658	146	18°	0.90
Ukhaa Tolgod protoceratopsid	IGM 100/3596	220	16°	0.96
Ukhaa Tolgod protoceratopsid	IGM 100/3501	323	12°	1.01
Ukhaa Tolgod protoceratopsid	IGM 100/1246	420	32°	1.02

Note

Measurements for *Protoceratops andrewsi* were taken from Dodson (1976, table 1), except for FMNH PR 14045, MPC D100/502, and MDP 530, which were measured for this study. Basal skull length was measured from the tip of the rostral to the caudal end of the quadrate. Frill inclination was measured as the angle between the sagittal crest and the maxillary tooth row. Relative frill length is the maximum length of the frill divided by the length of the remainder of the skull. The hyphen indicates that no well‐developed frill is present in the specimens. The asterisk denotes estimated length. All AMNH and FMNH specimens come from the Bayn Dzak locality, whereas the MDP and MPC specimens were collected at the Tugrikin Shire locality.

**TABLE 3:** Sample of *Triceratops horridus* specimens used in this study

Specimen	Basal skull length (mm)	Frill inclination	Relative frill length
UCMP 154452	280	?	0.27**
BMRP cast of MOR 2951	496	31°	0.65
BMRP 2006.4.1.2	854	32°**	0.78
YPM 1821	905	21°	0.91
YPM 1823	955	27°	0.72
FMNH PR 12300	1,054	20°	0.79
MPM VP6841	1,110**	?	?
AMNH 5116	1,147	38°	0.89

Note

Measurements for MOR 1120, and UCMP 154452 from Horner and Goodwin (2006), YPM 1823 from Hatcher et al. (1907), and YPM 1821 from Scannella and Horner (2010). The basal skull length is the distance between the tip of the rostrum and the caudal margin of the quadrate. Frill inclination was measured as the angle between the sagittal crest and the maxillary tooth row. Frill length is the maximum length of the frill relative to the length of the remainder of the skull. A single asterisk denotes those specimens used only in the SRVF analysis; double asterisks indicate estimated values.

Positive modularity results could result from including a greater number of evenly spaced semilandmarks than primary landmarks (Klingenberg, pers. Communication). To ensure that positive and statistically significant results were not just an artifact of such a bias, we devised a sensitivity analysis in which all of the above CR analyses were repeated with varying numbers (*n* = 10, 20, 28) of semilandmarks removed.

### 2.4 Heterochrony testing

We considered the definition of heterochrony as changes in the relative timing and rate of developmental events between ancestor and descendant species, as well as the onset and rate at which the processes responsible for such changes take place (Alberch, Gould, Oster, & Wake, 1979; Gould, 1977, 1992; Klingenberg, 1998; McKinney & McNamara, 1991). Typically, heterochronic processes are identified by analyzing allometric variation between taxa relative to developmental age (Meyer, 1987; Ryan and Semlitsch, 1998; Webster and Zelditch, 2005). In the absence of absolute specimen ages, we follow more recent studies (Drake and Klingenberg, 2007; Forth et al., 2016; Klingenberg & Marugán‐Lobón, 2013) in using centroid size as the independent variable for investigating ontogenetic allometry in the four species. We performed an initial regression of shape scores on log‐transformed centroid size for all terminals to determine whether there was evidence of allometry (Drake and Klingenberg, 2007) in the form of a significant slope. Following recommendations in Klingenberg and Marugán‐Lobón (2013), we also performed a regression of the phylogenetic independent contrasts (PIC) for both shape scores on log‐transformed centroid sizes using the tree shown in Figure 1. Slope and *F* test calculations were conducted in the software PAST (Hammer, Harper,& Ryan, 2008).

We followed analyses outlined in Forth et al. (2016) in order to determine whether allometric shifts and possibly heterochrony occurred in the evolutionary history of the ceratopsian frill. A pooled multivariate regression of Procrustes coordinates against log‐transformed centroid size was conducted on the sample of adult skulls (Tables 2 and 3), along with the smallest skull for each of the four species that were ontogenetically sampled. Ontogenetic vectors were reconstructed for those four species by connecting the smallest and largest sampled skulls. Ancestral ontogenetic vectors were subsequently reconstructed for Neoceratopsia, Coronosauria, and Protoceratopsidae by optimizing adult and juvenile values for regression scores and log centroid size on the time‐scaled phylogeny used for the pPCA analyses in the software Mesquite version 3.51 (Maddison & Maddison, 2018) using square‐changes parsimony. Because our sample of ceratopsian skull shapes is heavily skewed toward Late Cretaceous Ceratopsidae with long ghost lineages among more basal neoceratopsians, implementation of the Brusatte et al. (2008) branch smoothing algorithm for adjusting zero‐length branches leads to very deep branches subtending each species of protoceratopsid (~20 million years) and also *Liaoceratops* (>10 million years). Ongoing studies on basal neoceratopsian diversity (He et al., 2015; Morshhauser, You, Li, & Dodson, 2019) and ages and correlations of Asian Late Cretaceous red beds (Dashzeveg et al., 2005; Fanti, Currie, & Badamgarav, 2012) suggest that turnover among protoceratopsids may have occurred more rapidly within the Campano‐Maastrichtian (Makovicky, 2008). We therefore also generated ancestral ontogenetic vectors from optimizations on a tree in which branches without implied ghost lineage durations were corrected in Mesquite by addition of 1 million years, representing a punctuated model of evolution, to assess how sensitive resulting ontogenetic vectors are to branch lengths in the tree.

Allometric shifts were determined by subtracting adult regression score species values from reconstructed ancestral ones with positive differences interpreted as peramorphosis, and negative ones as paedomorphosis. Following Forth et al. (2016), we considered allometric shifts between individual ancestor‐descendant pairs as significant if they were greater than 1.5 times the confidence interval of the mean difference between all ancestor‐descendant pairs in our taxonomic sample. Differences between parallel vectors represent either predisplacement if the descendant vector is shifted toward the origin of the biplot, or postdisplacement if the reverse holds.

We estimated the rate of allometric change by dividing regression score differences between adjacent nodes and/ or tips by the length of the branch separating them, and we also employed comparative methods to examine whether the rate of frill evolution covaries with phylogeny, and whether it remained constant or varied over ceratopsian evolution. Comparative analyses were conducted in the software BayesTraits Ver. 3 (http://www.evolution.rdg.ac.uk) using the same tree topology and branch lengths as in the analyses for PIC and for the pPCA. We first calculated maximum likelihood scores for the dataset under several different model settings to determine which model best applies to it in its entirety, and used AICc values to estimate the best‐fitting overall model. Models evaluated were random walk, directional trend, and three models each favoring one of the three branch and/ or tree scaling parameters *κ*, *δ*, and *λ*. *λ* is a measure of whether trait evolution covaries with phylogeny with *λ* = 0 signifying evolution is independent of phylogeny and *λ* = 1 represents trait evolution covarying with phylogeny according to a random walk model. *κ* is a measure of how the length of individual branches scales to the rate of evolution. *δ* represents the scaling of trait evolution to path length (patristic distance from root to tips), with *δ* < 1 representing an “early burst” evolutionary scenario, *δ* > 1 often interpreted as accelerating evolution or “evolution via species adaptation,” and *δ* = 1 representing gradual evolution (Pagel, 1999).

Individual analyses investigating the effects of branch scaling parameters allow for a finer scaled interpretation of the dynamics of frill shape evolution. Following Hernandez et al. (2013), we performed separate analyses for each of the scaling parameters to estimate the optimal value. We then ran constrained analyses for each parameter set to either 0 or 1 representing the various evolutionary scenarios defined above and compared the fit of these to the values to the unconstrained analyses using Bayes factors. In order to determine whether evolutionary rates vary across the phylogeny, the Variable Rates protocol in BayesTraits ver. 3 was performed and marginal likelihoods were estimated for both models with rate variation and without.

Allometric differences related to shifts in ontogenetic vector angles between ancestor and descendant represent allometric acceleration or deceleration. Shifts in slope between ancestral and descendant ontogenetic vectors were measured as differences in their angles (Collyer & Adams, 2007) calculated as the arccosine of the dot product of the two vectors divided by the product of their lengths. We devised a simple randomization test to evaluate the significance of the angles. Using the largest and smallest components of the observed vectors as bounds, we generated 200 random vectors and measured the angles between successive pairs of random vectors using the same formula as for the observed vectors. The observed angles were compared against the distribution of one hundred randomly generated angles to see whether they fall within a probability tail, or not.

Because the above method only samples the largest and smallest individual of each of the four ontogenetically sampled species without accounting for the large individual variance in shape and size in the full samples, we performed a second pooled multivariate regression based on the full range of specimens for just the four species with growth series. We compared slopes for the ontogenetic vectors using *F* tests, but did not consider regression score differences as scores and log centroid size values were heavily biased by the differences in size range and sample sizes between the individual species (which is why sampling was limited to two individuals in the previous analysis). Slope and *F* test calculations were conducted in the software PAST (Hammer et al., 2008).

## 3 RESULTS

### 3.1 Interspecific frill variation in Ceratopsia and Neoceratopsia

Overall, variation in the ceratopsian frill (excluding frill ornamentation and parietal fenestrae) is concentrated along its caudal and caudolateral margins (Figure 2). For both Ceratopsia and Neoceratopsia, the first three PC axes accounted for about 60% of the variation (with PC through PC10 encompassing over 95% of the variation), and PC1 encapsulated only 25% of the overall variation (Table 4). Basal ceratopsians showed a nonoverlapping (albeit nonsignificant, Table 5) distribution with neoceratopsians, appearing more segregated in morphospace (Figure 2a,b). Significant (Table 5) nonoverlapping morphospace distributions were found between Centrosaurines and both basal Ceratopsia and basal Neoceratopsia. Nonceratopsid neoceratopsians and chasmosaurine ceratopsids showed overlapping distributions when considering variation of along PC2 (Figure 2a), due to the relatively greater lateral expansion of the frill of these two groups.

**TABLE 4:** Variance components for the first three PC axes for the Procrustes superimposition analyses for the two ceratopsian groups considered

PC axis	Ceratopsia	Neoceratopsia
PC1	25.4	24.4
PC2	19.1	18.3
PC3	17.2	16.5
PC4	9.8	9.4
PC5	6.7	8.3
PC6	5.5	5.6
PC7	3.9	4.4
PC8	3.5	3.4
PC9	2.9	2.8
PC10	1.7	2.5

**TABLE 5:** Results of the pairwise permutational ANOVAs computed between ceratopsian groups on each of the PC axes

pPC axis	Group 1	Group 2	*F*‐value	*df*1	*df*2	*p*‐Value
PC1	Centrosaurinae	Chasmosaurinae	29.15	1	14	0
PC1	Centrosaurinae	Basal Neoceratopsia	10.70	1	8	.007
PC1	Centrosaurinae	Basal Ceratopsia	149.64	1	8	.008
PC1	Chasmosaurinae	Basal Neoceratopsia	0.02	1	10	.926
PC1	Chasmosaurinae	Basal Ceratopsia	14.68	1	10	.004
PC1	Basal Neoceratopsia	Basal Ceratopsia	5.87	1	4	.142
PC2	Centrosaurinae	Chasmosaurinae	2.01	1	14	.186
PC2	Centrosaurinae	Basal Neoceratopsia	1.85	1	8	.219
PC2	Centrosaurinae	Basal Ceratopsia	1.01	1	8	.351
PC2	Chasmosaurinae	Basal Neoceratopsia	0.09	1	10	.752
PC2	Chasmosaurinae	Basal Ceratopsia	0.01	1	10	.926
PC2	Basal Neoceratopsia	Basal Ceratopsia	0.18	1	4	.617
PC3	Centrosaurinae	Chasmosaurinae	4.99	1	14	.053
PC3	Centrosaurinae	Basal Neoceratopsia	9.66	1	8	.021
PC3	Centrosaurinae	Basal Ceratopsia	72.78	1	8	.008
PC3	Chasmosaurinae	Basal Neoceratopsia	9.68	1	10	.012
PC3	Chasmosaurinae	Basal Ceratopsia	27.38	1	10	.001
PC3	Basal Neoceratopsia	Basal Ceratopsia	5.34	1	4	.104

Note

*p*‐Values are estimated by means of 1,000 randomizations. In this case, the parietal fenestra of the frill was excluded from the morphometric analysis.

Abbreviation: *df*, degrees of freedom.

There is a general trend of progressive caudal elongation and rostrolateral expansion of the caudal and lateral margins of the frill, respectively, from basal ceratopsians to centrosaurine ceratopsids (Figure 2a,b). Other components of frill variation include caudomedial extension of the caudal border (PC2, Figure 2a) and caudolateral projection of the caudolateral corner (PC3, Figure 2b) of the frill. Basal ceratopsians occupied the region of the morphospace defined by a rostrocaudally abbreviated caudal region of the skull, whereas centrosaurine ceratopsids are at the other end of the spectrum featuring a rostrocaudally elongate frill.

Morphospace occupation patterns in fenestrated neoceratopsians broadly mirror those found for the dataset that excluded the fenestra, despite the exclusion of several taxa with solid frills such as basal ceratopsians and the chasmosaurine ceratopsid *Triceratops*. Again, substantial overlap in morphospace occupation between chasmosaurines and basal coronosaurs is observed in the PC1 versus PC2 plot (Figure 3a), due to the greater lateral expansion of the frill in these two groups. However, when considering PC1 versus PC3, basal neoceratopsians, chasmosaurines, and centrosaurines occupy significantly distinct (Table 6) nonoverlapping regions of the morphospace (Figure 3b). Both the frill and parietal fenestra show a strong trend toward rostrocaudal lengthening from basal neoceratopsians to ceratopsids, particularly in centrosaurines, as captured by both PC1 and PC2 (Figure 3a).

**TABLE 6:** Results of the pairwise permutational ANOVAs computed between ceratopsian groups on each of the PC axes

pPC axis	Group 1	Group 2	*F*‐value	*df*1	*df*2	*p*‐Value
PC1	Centrosaurinae	Chasmosaurinae	21.79	1	13	0
PC1	Centrosaurinae	Basal Neoceratopsia	8.83	1	8	.005
PC1	Chasmosaurinae	Basal Neoceratopsia	0.05	1	9	.816
PC2	Centrosaurinae	Chasmosaurinae	9.32	1	13	.004
PC2	Centrosaurinae	Basal Neoceratopsia	2.06	1	8	.206
PC2	Chasmosaurinae	Basal Neoceratopsia	0.98	1	9	.359
PC3	Centrosaurinae	Chasmosaurinae	4.66	1	13	.05
PC3	Centrosaurinae	Basal Neoceratopsia	6.69	1	8	.047
PC3	Chasmosaurinae	Basal Neoceratopsia	7.35	1	9	.015

Note

*p*‐Values are estimated by means of 1,000 randomizations. In this case, the parietal fenestra of the frill was included in the morphometric analysis.

Abbreviation: *df*, degrees of freedom.

### 3.2 Tests of frill modularity

The recovered CR coefficients provided varying degrees of support for modularity of the frill in the four species and within different groupings of ceratopsians, with actual CR values consistently located at the tail end of the distribution of randomized values (Figures 4, 5, 6). Weak but significant support for modularity was found with CR values approaching one in the grade of basal ceratopsians (Figure 4a), Ceratopsia as a whole (Figure 4b) and in basal neoceratopsians (Figure 4c). Support for the modularity of the frill was stronger in the clades Neoceratopsia (Figure 4d,e) and Ceratopsidae (Figures 4f and 5a), as evidenced by the substantially lower CR and significance values. Within Ceratopsidae, consideration of each subclade separately resulted in weaker support for frill modularity (Figure 5b–d) likely due to sample size. However, inclusion of the parietal fenestra in the analysis resulted in higher support for modularity in Chasmosaurinae (lower CR value located nearer the end of the distribution) relative to Centrosaurinae.

Evidence for frill modularity was weaker, though still statistically significant, in the ontogenetic series of *Liaoceratops yanzigouensis,* the Ukhaa Tolgod protoceratopsid, *Protoceratops andrewsi* and *Triceratops horridus* (Figure 6d) as CR coefficients for all taxa approached unity (Figure 6). Furthermore, those CR values were located further from the tails of the distributions (Figure 6) than in the clade analyses reported above (Figures 4 and 5), indicating the existence of a number of possible landmark partitions in these four species with a greater potential for within‐module covariation than in the frill partition.

The iterative subsampling analyses with removal of 10, 20, and 28 semilandmarks resulted in very similar CR values for these ceratopsian groups as were recovered for the full dataset, although the spread of their distributions slightly increased progressively and CR values migrated slightly away from the tail end of the such distributions (Appendices [Link], [Link], [Link]). The latter effect was more noticeable after removal of 20 and 28 semilandmarks (Appendices S9 and S10). However, results indicate that our overall results were not biased by inclusion of a large number of semilandmarks.

### 3.3 Heterochrony of the frill

**FIGURE 8:**
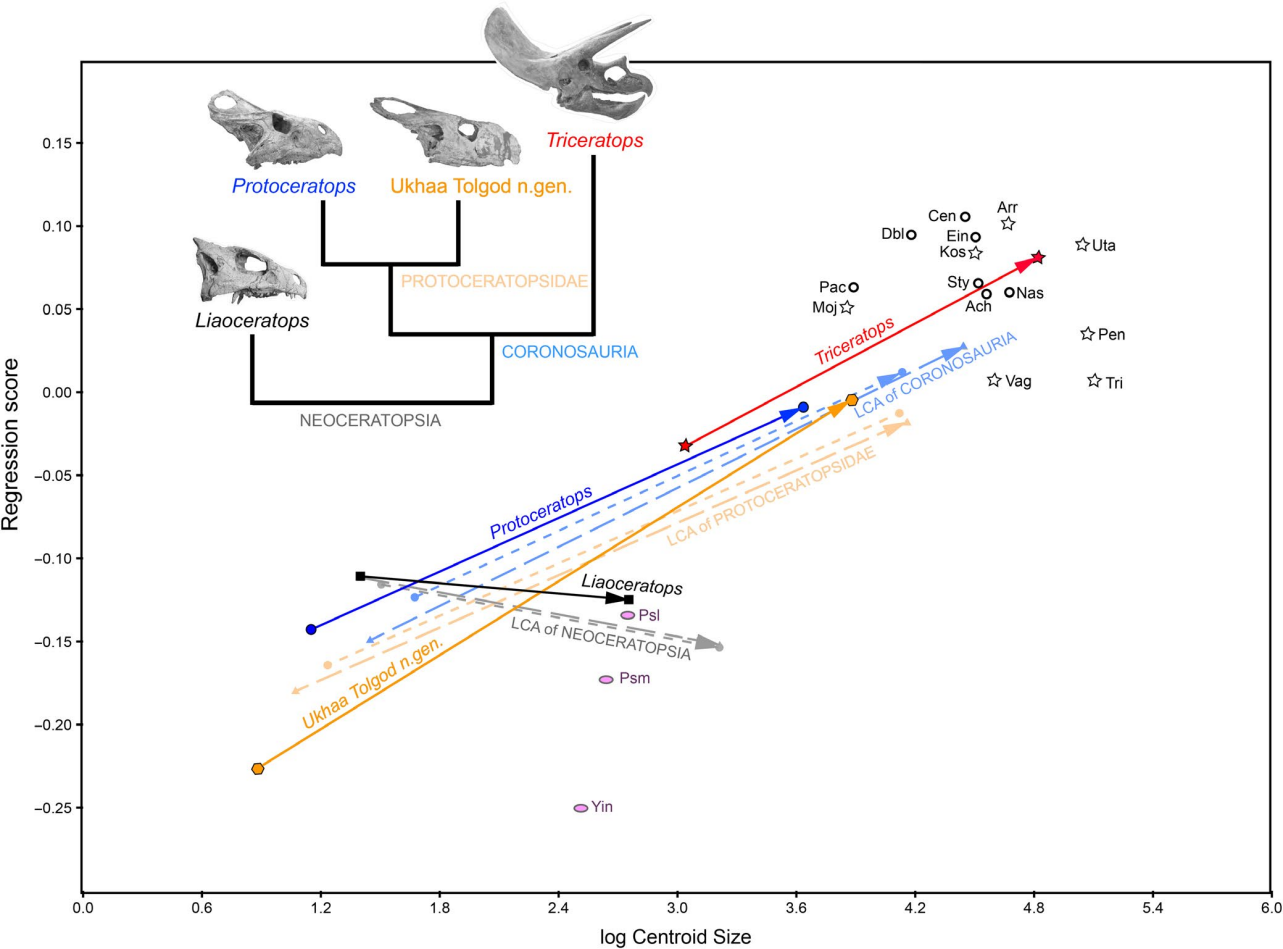
Regression analysis of ontogenetic trajectories from four neoceratopsian species against log‐transformed centroid size using the regression score as shape variable. Solid lines represent observed taxa. Ontogenetic vectors for reconstructed ancestors are dashed to distinguish them from species. Short dashed vectors with circular points indicate reconstructions based on trees for which branch lengths were smoothed using the Brusatte et al. (2008) method. Long dashed vectors with triangular data points show reconstructions based on trees calibrated against record with no branch smoothing, but in which 1 Ma is added to taxa with zero‐branch length ghost lineages (i.e., oldest sister taxon in a clade). Hollow stars and circles indicate chasmosaurine and centrosaurine ceratopsid species, respectively, and pink ellipses indicate basal ceratopsian species; the abbreviations for all those species are as in Table 1

We found a significant (*p*‐value = .0001) positive (slope = 0.196) allometric relationship between regression scores and log centroid size for adults across Ceratopsia, and this relationship was robust to phylogenetic correction using PIC values (*p*‐value = .0053; slope = 0.112; Figure 7). Reconstructed ontogenetic vectors for *Liaoceratops* and the last common ancestor (LCA) of Neoceratopsia are flat to slightly negative, but slopes are positive in the LCAs of Coronosauria and Protoceratopsidae, and in the individual species they subtend (Figure 8). Positive differences in regression scores greater than the significance threshold (Appendix S11) are observed between the LCAs of Ceratopsia and Neoceratopsia, between the LCAs of Neoceratopsia and Coronosauria, and between the latter and the LCA of Protoceratopsidae. Significant positive differences are also observed in the branches immediately subtending the LCA of Ceratopsidae and in the terminal branches for *Liaoceratops, Einiosaurus*, and *Chasmosaurus*. Significant negative differences are calculated for the LCA of Chasmosaurinae and the LCA of (*Regaliceratops, Triceratops*), in each of those two individual species, and also in *Protoceratops, Vagaceratops*, and *Pachyrhinosaurus*.

Dividing the Regression Score differences by branch lengths provides an estimated rate of heterochronic change (Appendix S11). When comparing these rates, it is notable that rates for LCAs of several clades including Coronosauria, Protoceratopsidae, Ceratopsidae, and Chasmosaurinae with significant regression score differences lie within the two lowest rate estimate quartiles, suggesting that heterochronic shifts were achieved by incremental changes over long periods. By contrast, rapid changes in frill shape are principally observed in individual ceratopsid species and shallow subclades, especially among Chasmosaurinae, which dominate the top quartile of estimated rate values (Appendix S11).

Likelihood estimation for the five different evolutionary models overwhelmingly favors random walk (= Brownian motion) as the best‐fitting model for frill shape evolution across ceratopsian diversity. All single parameter analyses therefore employed the random walk model. Results of our scaling parameter analyses in BayesTraits are given in Appendix S12. In our unconstrained analysis, the estimate for *L* = 0.88 but is not significantly different from either *L* = 0 (trait evolution independent of phylogeny) or *L* = 1 (trait evolution tracks phylogeny). Parsing this result at finer taxonomic levels, *L* = 0 in centrosaurine and chasmosaurine ceratopsids, but is closer to one in nonceratopsid taxa indicating an abrupt shift in this parameter during ceratopsian frill evolution. The branch scaling parameter was estimated to be 0.465 suggesting that shorter branches (i.e., those among ceratopsids) capture more evolutionary change than long branches. However, this model, while better supported, is not significantly different from either *K* = 0 or *K* = 1 in our dataset, so a gradual (i.e., scaled to branch length) model of trait evolution cannot be excluded. The best‐supported model for the path length scaling parameter is the one that estimates *D* = 1.9, but it is not significantly different from 1 as determined by log Bayes factor differences, again not allowing us to exclude gradual evolution. However, testing for variable rates found positive evidence for a rate shift in our data (log Bayes factor difference > 3). Over 28% percent of Bayesian Monte Carlo Markov Chain iterations posite a rate shift within Chasmosaurinae, with rates above 65% in the chasmosaurine subclade that includes *Chasmosaurus*, *Vagaceratops*, and *Mojoceratops*. Percentages of iterations within centrosaurines are mostly lower (18% < 32%) than among chasmosaurines, but are still higher than among any nodes and branches that subtend nonceratopsid taxa, mirroring the distribution for rate estimates reported in Appendix S11 and supporting one or more shifts in the rate of frill evolution in ceratopsids.

Angles between the ontogenetic vectors of Neoceratopsia and Coronosauria are more than three times greater than the angles between any other ancestor‐descendant pair of vectors in the analysis regardless of branch length adjustment method indicating heterochrony through an allometric shift in growth rate at this node (Figure 8). The observed angles are greater than 85% of angles generated in our randomization tests, suggesting that these differences are substantial, though not necessarily significant at the 5% level. All other angles between ontogenetic vectors do not differ noticeably from random.

Within Neoceratopsia, a progression toward higher centroid size and regression score is observed in the adult endpoint in the vectors for the LCAs of Coronosauria, Protoceratopsidae, and in *Triceratops*, even though their angles do not differ substantially. This shift toward larger values in later diverging (i.e., descendant) taxa is consistent with hypermorphosis according to Alberch et al. (1979) (Figure 8). However, because of the great difference in sampling of the youngest specimens between species (neonates in *Tengr* and *Protoceratops* versus older juveniles in *Liaoceratops* and *Triceratops*), we cannot assess whether or postdisplacement may also have contributed to these results.

## 4 DISCUSSION

### 4.1 Shape variation across Ceratopsia

Frill shape distributions in morphospace exhibit a phylogenetic pattern even after correction for phylogenetic covariance, with early‐diverging forms occupying their own region of morphospace distinct from neoceratopsians in all analyses they were included in (Figures 2 and 3). Maiorino, Farke, Kotsakis, and Piras (2017) also recovered a phylogenetic structuring in the cranial morphospace of most ceratopsian clades, although their morphometric analysis considered the ceratopsian skull in lateral view. We observed further phylogenetic structuring in morphospace within Neoceratopsia as well, with chasmosaurines overlapping in morphospace occupation (PC1/PC2) with early‐diverging neoceratopsians (Figures 2a and 3a), while centrosaurines occupy their own sector to the left of all other species (Figures 2a,b and 3). A greater separation between groups is observed in the PC1/PC3 morphospace, with early‐diverging taxa occupying the upper right quadrant and progressively later diverging taxa moving to the left along PC3 (caudally wider frills; Figures 2b and 3b).

Ceratopsian frills, and in particular those of ceratopsids, have been interpreted as highly variable by multiple authors (e.g., Brown & Henderson, 2015; Dodson & Currie, 1990; Dodson et al., 2004; Evans & Ryan, 2015; Knell & Sampson, 2011; Ostrom, 1966; Padian & Horner, 2011; Ryan et al., 2007; Sampson & Loewen, 2010). These qualitative observations are largely supported by our quantitative results with ceratopsids occupying unique and markedly larger part of morphospace than nonceratopsids. Notably, the expansion of frill morphospace in ceratopsids coincides with increased rates of shape change (Appendices S11 and S12), which can be interpreted as a conservative proxy for frill evolutionary rates since the elaboration of episquamosal and epiparietal ornamentation is not accounted for using our methodology. These results are in agreement with the accelerated evolutionary rates in cranial morphology found by Maiorino et al. (2017) in ceratopsids relative to early‐diverging clades. While in our study this pattern is undoubtedly related to the uneven sampling across ceratopsian clades and grades with a bias toward ceratopsids, qualitative comparisons to frill shape and anatomy among unsampled basal ceratopsians (Makovicky & Norell, 2006; Morshhauser et al., 2019; Xu, Makovicky, Wang, Norell, & You, 2002) indicates that they are generally similar to sampled noncoronosaurian taxa in being relatively short, narrow, and having a round profile with a gradual rather than stepped parietal‐squamosal contact in dorsal aspect. We therefore see no indication that they would markedly increase morphospace occupation of grades stemward of Coronosauria.

### 4.2 Modularity

We found a strong interspecific signal for modularity (Figures 4 and 5), but a weak (though still significant) signal at intraspecific levels within ontogeny (Figure 6). Recently, Maiorino et al. (2017) found in ceratopsians a greater degree of integration between the frill and the mandible than between the frill and rest of the skull, which is consistent with the interspecific modularity of the frill hypothesized in our study. The weak modularity signal at intraspecific levels within ontogeny may relate to smaller samples sizes for individual species compared to clades, and also the greater effects of taphonomic distortion on the more limited morphospace of intraspecific samples. Other morphometric analyses of ceratopsian species including *Psittacosaurus lujiatunesis* (Hedrick & Dodson, 2013) and *Protoceratops andrewsi* (Maiorino, Farke, Kotsakis, & Piras, 2015) also recovered a large amount of taphonomically induced shape variation that overruled earlier studies suggesting greater taxonomic diversity (You & Dodson, 2004) or sexual dimorphism (Dodson, 1976) for the two taxa respectively. Despite the taphonomic effects at species level, our results are congruent with the well‐established hypothesis positing that modularity is requisite for heterochronic evolution (Gerber & Hopkins, 2011; Olson & Rosell, 2006; Raff & Raff, 2000).

Studies on other vertebrates have shown that ontogenetic patterns of variation in modularity and integration are variable, depending on the skull components and characters (Goswami, Polly, Mock, & Sánchez‐Villagra, 2012; Klenovsek & Jojic, 2016; Zelditch & Carmichael, 1989) and taxa (Goswami & Polly, 2010; Willmore, Leamy, & Hallgrimsson, 2006; Zelditch, Bookstein, & Lundrigan, 1992) under consideration. The decrease in frill modularity within ontogenetic series of select species relative to that we observe at macroevolutionary levels in ceratopsians doubtless reflects taphonomic distortion as noted above, with the larger shape differences between exemplars of different species less affected by such artifacts, than are the smaller differences between specimens of a single species. However, we cannot exclude that some of this difference in the robustness of our CR results above and below the species level could be explained by the “palimpsest” metaphor (Hallgrímsson et al., 2009). According to this concept, observed patterns of modularity or integration in a particular specimen of a developmental series are the cumulative effects of all previous processes that occurred prior to that stage (Klingenberg, 2014). Processes taking place in later ontogenetic stages may only partially “overwrite” the patterns that occurred in previous stages. In this way, the overall result of overlaying modular patterns on each other throughout development may lead to a higher degree of integration where there is less statistical support for modularity, even though the processes acting on the structure under study do so in a modular fashion (Klingenberg, 2014).

### 4.3 Heterochrony

As one of the first quantitative studies of ceratopsian cranial shape, we found substantial support for heterochronic processes in the evolution of the hallmark parieto‐squamosal frill that diagnoses the clade. Acceleration is the main mechanism that accounts for the macroevolutionary shift in frill shape and size between the LCA of Neoceratopsia with a short, narrow frill throughout ontogeny, and coronosaurs, which have significantly longer and wider frills in adults (Hone et al., 2016). Furthermore, shifts in Regression Scores correlated with shifts in centroid size between early‐diverging coronosaurs and the *Triceratops* ontogenetic vector are broadly consistent with a peramorphocline between early‐diverging coronosaurs and ceratopsids as suggested by Long and McNamara (1995), although the exact details differ.

Previous discussions of heterochrony in ceratopsians have focused heavily on ceratopsids. Because of the lack of growth series for almost all taxa, our analysis cannot quantitatively evaluate these previous qualitative generalizations (e.g., Long & McNamara, 1995; McNamara & Long, 2012; Tumarkin & Dodson, 1998), but we note that high rates of frill shape change accompany both negative and positive allometric shifts between adults of various species and their respective LCAs, indicating that a combination of both paedomorphic and peramorphic processes underpin the great disparity of ceratopsian frill shapes. Such mosaic heterochrony may facilitate the evolution of disparate morphologies (Guerrant, 1982), which is consistent with the morphospace occupation patterns we observe in ceratopsids (Figures 2 and 7a).

Although we provide the first strong quantitative evidence for heterochrony in ceratopsian skull evolution, heterochrony alone is most likely insufficient as a mechanism to explain frill disparity. Innovation was probably an important process in ceratopsian diversification, especially through the evolution of the episquamosal and epiparietal structures that are characteristic of Ceratopsidae. These structures are, however, difficult to homologize for the purposes of identifying landmarks suitable for geometric morphometric analysis, and the quantitative analysis of their morphology will require other methods such as outline‐based techniques (e.g., Joshi, Prieto‐Márquez, & Parker, 2011) to capture the true disparity of frill forms.

Overall patterns of morphospace occupation (Figure 2) support the results of our analyses of evolutionary rates (Appendices S11 and S12), which reveal a marked shift in the tempo and mode of frill evolution. Morphospace occupation and evolutionary rates are constrained in early‐diverging nonceratopsid taxa, with frill evolution happening gradually and largely predicted by phylogeny. Ceratopsid frill evolution, on the other hand, is characterized by significantly greater disparity driven by evolution at elevated rates that is independent of phylogeny. Elucidating the biological drivers behind this change is challenging, as they are likely multiple and complex, but the ceratopsian literature (e.g., Makovicky, 2012; Makovicky & Norell, 2006) suggests that the observed acceleration between the neoceratopsian LCA and coronosaurs, as well as subsequent high rates of evolution in ceratopsids, may relate to a shift in frill function.

Although myriad hypotheses have been put forth for the function of the ceratopsian frill, recent studies favor either feeding (e.g., Maiorino et al., 2017) or intraspecific signaling (e.g., Horner & Goodwin, 2006) as the primary function for this structure. The frills of basal neoceratopsians exhibit a short, deep morphology with muscle scars along most of the rim providing evidence of the insertion of enlarged jaw adductor muscles (Makovicky & Norell, 2006; Morshhauser et al., 2019; Xu et al., 2002). Muscle scarring is much less developed in the expanded frills of coronosaurs. Current reconstructions of jaw muscles in these animals place the jaw adductor origins closer to the rostral edge of the supratemporal fenestra, along the midline keel of the frill, and along the inner surface of the squamosal, with the majority of the frill, including its caudal margin, free of musculature (Dodson, 1996; Nabavizadeh, 2018). These differences in frill anatomy between early and late diverging ceratopsians prompted Makovicky (2012) and Makovicky and Norell (2006) to propose that the ceratopsian frill underwent a shift in its primary function within Neoceratopsia from providing an increased area for attachment of jaw adductor muscles as (Haas, 1955; Lull, 1908; Ostrom, 1964; Xu et al., 2002), to serving as a species‐specific display structure with possible social and/or sexual functions (Dodson, 1976, 1993, 1996; Farlow & Dodson, 1975; Hone, Naish, & Cuthill, 2012; Hone et al., 2016; Horner & Goodwin, 2006, 2008; O'Brien et al., 2018; Padian & Horner, 2011; Sampson, Ryan, & Tanke, 1997). Insertion of jaw musculature along the majority of the frill margin in early‐diverging taxa such as *Liaoceratops*, *Yamaceratops* (Makovicky & Norell, 2006), and *Auroraceratops* (Morshhauser et al., 2019) would likely have constrained allometric growth patterns by optimizing muscle orientations and mechanical leverage, a conclusion supported by the observed absence of ontogenetic shape change in *Liaoceratops* and the LCA of Neoceratopsia (Figure 7a). However, both anatomical data (Dodson, 1993; Farke, Chapman, & Andersen, ; Nabavizadeh, 2019; Rieppel, 1981) and biomechanical models (Bell, Snively, & Shychoski, 2009) support the conclusion that most of the frill margin was free of muscular attachment in ceratopsids, and likely all coronosaurs. Thus released from ancestral constraints, the frill was able to respond more rapidly to other selective pressures such as sociosexual signaling (Hone et al., 2012; Horner & Goodwin, 2006, 2008; Sampson et al., 1997).

While our morphometric and comparative analyses cannot directly test such functional hypotheses, they can be used to evaluate predictions arising from them. For the frill to shift function in ceratopsian evolution, it has to first represent a discrete functional unit, an inference that is consistent with our results for modularity across multiple clades and grades of ceratopsian evolution and also within individual species. Secondly, we predict that a shift in the primary function of a modular trait should be mirrored in shifts in morphospace occupation. This prediction is also met by our morphospace results (Figures 2 and 3) where ceratopsids occupy discrete morphospace from basal ceratopsians characterized by more expanded and variable frills regardless of whether or not fenestrae are excluded. Finally, if the shift in frill function entails a functional release of the caudal frill border from serving as anchorage for jaw adductor muscles (Dodson, 1996; Makovicky, 2012) and frees it up to evolve in response to other selective pressures, we may predict that frills exhibit greater disparity and an increased rate of evolution. Both of these predictions are also met by our results, with our comparative analyses revealing that the increased disparity of ceratopsid frills was achieved by an increase in evolutionary rates and a decoupling of trait evolution from phylogeny. It is worth stressing that these patterns are robust even without considering the highly disparate epiparietal and episquamosal ossifications that characterize ceratopsid frill ornamentation.

## 5 CONCLUSIONS

The parieto‐squamosal frill of ceratopsian dinosaurs is a neomorphic structure that has been proposed to have evolved via heterochrony. Through geometric morphometric methods, we demonstrate that frill shapes vary by clade and also that the highly variable frills of later diverging ceratopsids occupy a greater area of morphospace relative to the more constrained occupation of earlier diverging taxa. Our morphometric analyses demonstrate that the principal mechanism of macroevolutionary frill shape is expansion along the caudolateral “corners” and caudal margin of the frill. In the more derived ornamented ceratopsians that radiated during the Campanian and Maastrichtian (latest Cretaceous), morphological variation is concentrated along the caudolateral (Chasmosaurinae) and lateral (Centrosaurinae) margins of the frill.

The frill constituted a module at both macroevolutionary, and to a lesser extent, ontogenetic levels, and evolved via peramorphosis within Ceratopsia. This included acceleration of frill shape change early in ceratopsian history between early‐diverging neoceratopsians and coronosaurs, followed by peramorphosis, possibly by hypermorphosis, between early‐diverging coronosaurs and the ceratopsid radiation. Within the latter radiation, fast rates of frill shape evolution decoupled from phylogeny produced both peramorphic and paedomorphic shape changes, indicating that mosaic heterochrony accounts for the great disparity in frill shapes. The constrained morphospace occupation and low rates of frill shape evolution among early‐diverging ceratopsians when compared to ceratopsids are consistent with a shift in frill function from primarily serving as a platform for enlarged jaw adductor muscles, to being a highly variable display structure. This hypothesis along with better insights on the heterochronic processes within ceratopsids await a denser sampling within ceratopsian growth series and across ceratopsian diversity. Nevertheless, our study provides the first quantitative evidence supporting previous assertions that peramorphosis played a key role in the early evolution of the ceratopsian frill.

## CONFLICT OF INTERESTS

None declared.

## AUTHOR CONTRIBUTION

**Albert Prieto‐Márquez:** Conceptualization (equal); Data curation (equal); Formal analysis (equal); Funding acquisition (equal); Investigation (equal); Methodology (equal); Project administration (equal); Resources (equal); Software (equal); Supervision (equal); Validation (equal); Visualization (equal); Writing‐original draft (equal); Writing‐review & editing (equal). **Joan Garcia‐Porta:** Formal analysis (equal); Investigation (equal); Methodology (equal); Software (equal); Supervision (equal); Validation (equal); Visualization (equal); Writing‐review & editing (equal). **Shantanu H. Joshi:** Formal analysis (equal); Investigation (equal); Methodology (equal); Software (equal). **Mark A. Norell:** Data curation (equal); Resources (equal); Supervision (equal); Validation (equal); Visualization (equal); Writing‐review & editing (equal). **Peter J. Makovicky:** Conceptualization (equal); Data curation (equal); Formal analysis (equal); Funding acquisition (equal); Investigation (equal); Methodology (equal); Resources (equal); Software (equal); Supervision (equal); Validation (equal); Visualization (equal); Writing‐original draft (equal); Writing‐review & editing (equal).

## Supporting information

Appendix S1

Appendix S2

Appendix S3

Appendix S4

Appendix S5

Appendix S6

Appendix S7

Appendix S8

Appendix S9

Appendix S10

Appendix S11

Appendix S12

## Data Availability

The data used in this research that is not included in the tables are provided in the appendices listed above.
