# Supplementary material for: Modularity and heterochrony in the evolution of the ceratopsian dinosaur frill
Source: Ecol Evol. 2020 May 22;10(13):6288–309. doi: 10.1002/ece3.6361 (PMC7381594; doi:10.1002/ece3.6361)
Supplement: Supplementary file 2 — Appendix S2 [file ECE3-10-6288-s002.pdf]

Appendix 2. Landmark and semilandmark coordinates for the sample of Neoceratopsian species (parietal fenestra included)

LM=59
268.00000 7.00000
271.00000 357.00000
229.00000 379.00000
129.00000 337.00000
152.00000 521.00000
52.00000 761.00000
259.00000 776.00000
438.00000 761.00000
379.00000 529.00000
305.00000 386.00000
407.00000 343.00000
119.00000 405.00000
48.00000 497.00000
31.00000 595.00000
32.00000 672.00000
138.00000 775.00000
180.00000 776.00000
212.00000 776.00000
233.00000 777.00000
319.00000 778.00000
366.00000 775.00000
393.00000 770.00000
414.00000 766.00000
496.00000 699.00000
495.00000 642.00000
489.00000 583.00000
487.00000 512.00000
197.00000 391.00000
185.00000 413.00000
170.00000 432.00000
153.00000 463.00000
339.00000 399.00000
350.00000 425.00000
368.00000 459.00000
381.00000 491.00000
212.00000 546.00000
139.00000 665.00000
159.00000 690.00000
231.00000 622.00000
312.00000 549.00000
312.00000 658.00000
361.00000 693.00000
387.00000 671.00000
87.00000 315.00000
448.00000 323.00000
239.00000 56.00000
230.00000 95.00000
211.00000 148.00000
205.00000 191.00000

181.00000 229.00000
165.00000 243.00000
122.00000 280.00000
296.00000 46.00000
310.00000 79.00000
316.00000 117.00000
327.00000 162.00000
332.00000 202.00000
355.00000 233.00000
394.00000 266.00000
ID=Arrhinoceratops
SCALE=0.079399
LM=59
373.00000 23.00000
363.00000 442.00000
319.00000 445.00000
244.00000 368.00000
268.00000 542.00000
84.00000 756.00000
372.00000 865.00000
691.00000 785.00000
502.00000 538.00000
425.00000 442.00000
501.00000 368.00000
205.00000 440.00000
157.00000 498.00000
140.00000 549.00000
122.00000 618.00000
77.00000 838.00000
84.00000 884.00000
148.00000 934.00000
211.00000 926.00000
431.00000 897.00000
486.00000 934.00000
551.00000 948.00000
639.00000 926.00000
669.00000 735.00000
652.00000 653.00000
640.00000 568.00000
617.00000 468.00000
218.00000 577.00000
171.00000 621.00000
143.00000 682.00000
118.00000 728.00000
548.00000 590.00000
583.00000 637.00000
612.00000 677.00000
637.00000 718.00000
331.00000 552.00000
87.00000 796.00000
187.00000 908.00000
347.00000 809.00000
423.00000 562.00000
410.00000 819.00000

570.00000 924.00000
676.00000 839.00000
212.00000 337.00000
535.00000 334.00000
348.00000 72.00000
329.00000 123.00000
321.00000 171.00000
315.00000 224.00000
310.00000 264.00000
285.00000 285.00000
254.00000 310.00000
401.00000 73.00000
411.00000 113.00000
416.00000 157.00000
416.00000 196.00000
421.00000 234.00000
444.00000 279.00000
470.00000 304.00000
ID=Mojoceratops
SCALE=0.027753
LM=59
135.00000 19.00000
133.00000 307.00000
112.00000 309.00000
80.00000 244.00000
56.00000 346.00000
33.00000 361.00000
140.00000 480.00000
251.00000 372.00000
221.00000 351.00000
155.00000 307.00000
186.00000 240.00000
71.00000 264.00000
53.00000 276.00000
36.00000 286.00000
22.00000 321.00000
36.00000 389.00000
45.00000 413.00000
54.00000 444.00000
70.00000 470.00000
166.00000 489.00000
193.00000 489.00000
224.00000 464.00000
238.00000 435.00000
247.00000 333.00000
239.00000 308.00000
224.00000 286.00000
196.00000 274.00000
95.00000 312.00000
84.00000 321.00000
73.00000 329.00000
65.00000 336.00000
169.00000 315.00000
184.00000 326.00000

196.00000 332.00000
209.00000 341.00000
108.00000 368.00000
74.00000 388.00000
89.00000 438.00000
125.00000 440.00000
159.00000 377.00000
160.00000 439.00000
196.00000 446.00000
210.00000 378.00000
68.00000 234.00000
199.00000 221.00000
121.00000 48.00000
121.00000 77.00000
118.00000 104.00000
118.00000 136.00000
114.00000 167.00000
97.00000 189.00000
85.00000 210.00000
145.00000 49.00000
145.00000 74.00000
148.00000 101.00000
153.00000 134.00000
154.00000 155.00000
163.00000 182.00000
184.00000 201.00000
ID=Einiosaurus
SCALE=0.135531
LM=59
279.00000 9.00000
277.00000 361.00000
231.00000 362.00000
143.00000 250.00000
108.00000 440.00000
95.00000 562.00000
277.00000 599.00000
463.00000 562.00000
449.00000 442.00000
327.00000 364.00000
415.00000 247.00000
140.00000 310.00000
102.00000 369.00000
58.00000 404.00000
60.00000 502.00000
128.00000 595.00000
171.00000 602.00000
209.00000 602.00000
237.00000 600.00000
313.00000 599.00000
354.00000 600.00000
396.00000 602.00000
425.00000 595.00000
482.00000 528.00000
501.00000 486.00000

507.00000 420.00000
467.00000 379.00000
209.00000 374.00000
181.00000 390.00000
149.00000 407.00000
127.00000 422.00000
360.00000 380.00000
386.00000 405.00000
410.00000 416.00000
431.00000 430.00000
223.00000 459.00000
182.00000 481.00000
187.00000 514.00000
252.00000 486.00000
337.00000 459.00000
305.00000 490.00000
362.00000 516.00000
380.00000 492.00000
135.00000 225.00000
420.00000 227.00000
247.00000 32.00000
235.00000 63.00000
226.00000 93.00000
218.00000 126.00000
209.00000 156.00000
194.00000 180.00000
167.00000 196.00000
312.00000 36.00000
324.00000 60.00000
329.00000 93.00000
339.00000 128.00000
349.00000 161.00000
365.00000 187.00000
391.00000 198.00000
ID=Regaliceratops
SCALE=0.148992
LM=59
343.00000 21.00000
349.00000 675.00000
284.00000 676.00000
195.00000 593.00000
226.00000 764.00000
170.00000 1071.00000
357.00000 1033.00000
541.00000 1071.00000
465.00000 763.00000
407.00000 675.00000
504.00000 590.00000
186.00000 676.00000
106.00000 722.00000
94.00000 826.00000
107.00000 948.00000
203.00000 1064.00000
239.00000 1061.00000

287.00000 1053.00000
321.00000 1040.00000
396.00000 1037.00000
429.00000 1048.00000
465.00000 1053.00000
492.00000 1060.00000
589.00000 1020.00000
597.00000 948.00000
600.00000 855.00000
606.00000 743.00000
268.00000 694.00000
258.00000 714.00000
252.00000 732.00000
240.00000 749.00000
420.00000 691.00000
427.00000 707.00000
437.00000 722.00000
448.00000 743.00000
270.00000 880.00000
228.00000 968.00000
251.00000 1000.00000
301.00000 979.00000
448.00000 871.00000
420.00000 972.00000
475.00000 989.00000
493.00000 956.00000
163.00000 578.00000
532.00000 570.00000
304.00000 112.00000
300.00000 178.00000
299.00000 250.00000
289.00000 329.00000
267.00000 402.00000
246.00000 475.00000
210.00000 525.00000
376.00000 94.00000
383.00000 158.00000
391.00000 236.00000
397.00000 297.00000
411.00000 354.00000
423.00000 413.00000
449.00000 467.00000
ID=Kosmoceratops
SCALE=0.059346
LM=59
502.00000 25.00000
497.00000 654.00000
434.00000 689.00000
247.00000 723.00000
192.00000 1126.00000
117.00000 1321.00000
489.00000 1318.00000
890.00000 1306.00000
790.00000 1066.00000

566.00000 689.00000
740.00000 722.00000
218.00000 823.00000
150.00000 910.00000
70.00000 995.00000
33.00000 1132.00000
202.00000 1324.00000
263.00000 1318.00000
337.00000 1316.00000
411.00000 1318.00000
574.00000 1318.00000
658.00000 1315.00000
740.00000 1318.00000
797.00000 1323.00000
962.00000 1205.00000
943.00000 1066.00000
900.00000 941.00000
784.00000 834.00000
378.00000 783.00000
334.00000 889.00000
291.00000 970.00000
249.00000 1036.00000
619.00000 780.00000
653.00000 865.00000
703.00000 939.00000
745.00000 997.00000
403.00000 1092.00000
157.00000 1215.00000
195.00000 1252.00000
437.00000 1211.00000
532.00000 1091.00000
534.00000 1219.00000
840.00000 1203.00000
803.00000 1105.00000
221.00000 701.00000
766.00000 704.00000
463.00000 95.00000
431.00000 180.00000
413.00000 274.00000
390.00000 365.00000
363.00000 461.00000
332.00000 538.00000
295.00000 612.00000
545.00000 114.00000
576.00000 216.00000
600.00000 317.00000
613.00000 391.00000
648.00000 514.00000
677.00000 593.00000
711.00000 647.00000
ID=Triceratops
SCALE=0.021579
LM=59
502.00000 25.00000

497.00000 654.00000
434.00000 689.00000
247.00000 723.00000
192.00000 1126.00000
117.00000 1321.00000
489.00000 1318.00000
890.00000 1306.00000
790.00000 1066.00000
566.00000 689.00000
740.00000 722.00000
218.00000 823.00000
150.00000 910.00000
70.00000 995.00000
33.00000 1132.00000
202.00000 1324.00000
263.00000 1318.00000
337.00000 1316.00000
411.00000 1318.00000
574.00000 1318.00000
658.00000 1315.00000
740.00000 1318.00000
797.00000 1323.00000
962.00000 1205.00000
943.00000 1066.00000
900.00000 941.00000
784.00000 834.00000
378.00000 783.00000
334.00000 889.00000
291.00000 970.00000
249.00000 1036.00000
619.00000 780.00000
653.00000 865.00000
703.00000 939.00000
745.00000 997.00000
403.00000 1092.00000
157.00000 1215.00000
195.00000 1252.00000
437.00000 1211.00000
532.00000 1091.00000
534.00000 1219.00000
840.00000 1203.00000
803.00000 1105.00000
221.00000 701.00000
766.00000 704.00000
463.00000 95.00000
431.00000 180.00000
413.00000 274.00000
390.00000 365.00000
363.00000 461.00000
332.00000 538.00000
295.00000 612.00000
545.00000 114.00000
576.00000 216.00000
600.00000 317.00000

613.00000 391.00000
648.00000 514.00000
677.00000 593.00000
711.00000 647.00000
ID=Vagaceratops
SCALE=0.043713
LM=59
192.00000 0.00000
191.00000 356.00000
160.00000 336.00000
77.00000 256.00000
70.00000 371.00000
33.00000 391.00000
186.00000 595.00000
346.00000 389.00000
315.00000 366.00000
224.00000 336.00000
309.00000 251.00000
77.00000 290.00000
45.00000 306.00000
28.00000 332.00000
32.00000 366.00000
30.00000 448.00000
44.00000 490.00000
85.00000 530.00000
124.00000 573.00000
228.00000 593.00000
271.00000 556.00000
308.00000 518.00000
351.00000 459.00000
348.00000 366.00000
351.00000 334.00000
340.00000 308.00000
303.00000 282.00000
136.00000 331.00000
118.00000 338.00000
99.00000 347.00000
87.00000 356.00000
251.00000 333.00000
274.00000 340.00000
287.00000 348.00000
299.00000 359.00000
166.00000 395.00000
139.00000 393.00000
136.00000 505.00000
161.00000 511.00000
217.00000 396.00000
221.00000 511.00000
251.00000 501.00000
245.00000 394.00000
55.00000 227.00000
326.00000 227.00000
145.00000 18.00000
130.00000 51.00000

115.00000 73.00000
103.00000 110.00000
112.00000 149.00000
106.00000 172.00000
93.00000 200.00000
248.00000 41.00000
261.00000 69.00000
275.00000 97.00000
275.00000 129.00000
272.00000 159.00000
280.00000 178.00000
289.00000 201.00000
ID=Pachyrhinosaurus
SCALE=0.052371
LM=59
357.00000 14.00000
353.00000 513.00000
304.00000 525.00000
168.00000 509.00000
154.00000 737.00000
123.00000 1011.00000
345.00000 982.00000
570.00000 1017.00000
536.00000 748.00000
391.00000 525.00000
532.00000 513.00000
115.00000 571.00000
46.00000 611.00000
20.00000 701.00000
58.00000 837.00000
194.00000 1077.00000
259.00000 1069.00000
291.00000 1034.00000
320.00000 1001.00000
377.00000 1009.00000
400.00000 1058.00000
463.00000 1079.00000
533.00000 1069.00000
608.00000 962.00000
634.00000 869.00000
660.00000 738.00000
648.00000 612.00000
283.00000 549.00000
254.00000 593.00000
222.00000 632.00000
183.00000 668.00000
417.00000 568.00000
452.00000 605.00000
499.00000 645.00000
524.00000 686.00000
307.00000 708.00000
180.00000 702.00000
138.00000 985.00000
200.00000 1037.00000

385.00000 706.00000
503.00000 1034.00000
556.00000 985.00000
511.00000 700.00000
130.00000 507.00000
566.00000 511.00000
320.00000 82.00000
308.00000 154.00000
299.00000 227.00000
285.00000 290.00000
259.00000 351.00000
226.00000 399.00000
176.00000 453.00000
392.00000 74.00000
407.00000 132.00000
415.00000 206.00000
429.00000 306.00000
447.00000 355.00000
488.00000 411.00000
527.00000 467.00000
ID=Utaceratops
SCALE=0.089271
LM=59
269.00000 23.00000
268.00000 483.00000
215.00000 458.00000
130.00000 340.00000
124.00000 522.00000
66.00000 547.00000
275.00000 775.00000
485.00000 552.00000
453.00000 527.00000
333.00000 466.00000
392.00000 353.00000
131.00000 382.00000
124.00000 418.00000
110.00000 457.00000
81.00000 505.00000
64.00000 615.00000
75.00000 658.00000
99.00000 713.00000
156.00000 762.00000
328.00000 778.00000
397.00000 773.00000
445.00000 734.00000
484.00000 634.00000
458.00000 512.00000
434.00000 481.00000
411.00000 445.00000
388.00000 408.00000
191.00000 463.00000
172.00000 467.00000
159.00000 479.00000
141.00000 498.00000

364.00000 475.00000
393.00000 489.00000
415.00000 498.00000
434.00000 507.00000
236.00000 553.00000
116.00000 535.00000
137.00000 690.00000
245.00000 694.00000
309.00000 553.00000
314.00000 715.00000
425.00000 703.00000
424.00000 547.00000
95.00000 309.00000
435.00000 315.00000
243.00000 62.00000
232.00000 115.00000
217.00000 171.00000
207.00000 225.00000
203.00000 262.00000
178.00000 288.00000
142.00000 291.00000
289.00000 73.00000
303.00000 118.00000
307.00000 164.00000
318.00000 223.00000
329.00000 258.00000
339.00000 285.00000
392.00000 294.00000
ID=Nasutoceratops
SCALE=0.090099
LM=59
378.00000 9.00000
366.00000 385.00000
343.00000 397.00000
196.00000 382.00000
84.00000 632.00000
30.00000 686.00000
362.00000 786.00000
687.00000 652.00000
593.00000 565.00000
384.00000 396.00000
519.00000 373.00000
181.00000 457.00000
137.00000 516.00000
88.00000 569.00000
59.00000 605.00000
54.00000 734.00000
95.00000 768.00000
155.00000 786.00000
237.00000 793.00000
413.00000 791.00000
469.00000 786.00000
552.00000 770.00000
633.00000 739.00000

664.00000 584.00000
617.00000 538.00000
581.00000 498.00000
548.00000 443.00000
276.00000 433.00000
232.00000 470.00000
182.00000 521.00000
129.00000 567.00000
442.00000 420.00000
485.00000 453.00000
524.00000 496.00000
566.00000 535.00000
333.00000 578.00000
82.00000 678.00000
114.00000 738.00000
343.00000 731.00000
388.00000 575.00000
388.00000 741.00000
599.00000 714.00000
584.00000 577.00000
134.00000 389.00000
574.00000 391.00000
346.00000 69.00000
332.00000 118.00000
295.00000 181.00000
265.00000 227.00000
229.00000 268.00000
196.00000 307.00000
166.00000 346.00000
414.00000 65.00000
419.00000 115.00000
433.00000 166.00000
457.00000 216.00000
489.00000 258.00000
515.00000 287.00000
536.00000 335.00000
ID=Protoceratops
SCALE=0.029170
LM=59
352.00000 8.00000
354.00000 532.00000
313.00000 535.00000
192.00000 422.00000
177.00000 590.00000
91.00000 681.00000
343.00000 866.00000
625.00000 739.00000
520.00000 611.00000
400.00000 535.00000
527.00000 450.00000
170.00000 488.00000
147.00000 537.00000
118.00000 573.00000
82.00000 615.00000

72.00000 776.00000
79.00000 838.00000
128.00000 911.00000
217.00000 925.00000
415.00000 879.00000
502.00000 935.00000
573.00000 887.00000
614.00000 818.00000
648.00000 643.00000
606.00000 588.00000
584.00000 550.00000
537.00000 509.00000
275.00000 529.00000
257.00000 538.00000
233.00000 546.00000
205.00000 561.00000
442.00000 544.00000
463.00000 560.00000
484.00000 572.00000
509.00000 589.00000
260.00000 659.00000
182.00000 667.00000
164.00000 830.00000
278.00000 792.00000
419.00000 677.00000
413.00000 805.00000
514.00000 851.00000
561.00000 732.00000
170.00000 401.00000
540.00000 431.00000
332.00000 72.00000
314.00000 131.00000
297.00000 192.00000
291.00000 243.00000
274.00000 298.00000
235.00000 348.00000
204.00000 377.00000
389.00000 67.00000
408.00000 119.00000
430.00000 183.00000
442.00000 246.00000
452.00000 305.00000
471.00000 344.00000
507.00000 383.00000
ID=Centrosaurus
SCALE=0.057365
LM=59
300.00000 12.00000
301.00000 464.00000
270.00000 449.00000
160.00000 422.00000
164.00000 691.00000
142.00000 917.00000
302.00000 838.00000

451.00000 926.00000
445.00000 716.00000
329.00000 448.00000
441.00000 419.00000
53.00000 493.00000
62.00000 624.00000
102.00000 733.00000
124.00000 853.00000
187.00000 924.00000
238.00000 899.00000
260.00000 865.00000
275.00000 845.00000
336.00000 855.00000
349.00000 893.00000
395.00000 937.00000
428.00000 933.00000
474.00000 842.00000
493.00000 735.00000
537.00000 617.00000
555.00000 506.00000
215.00000 471.00000
192.00000 521.00000
172.00000 571.00000
164.00000 635.00000
382.00000 482.00000
410.00000 528.00000
434.00000 589.00000
445.00000 652.00000
262.00000 603.00000
195.00000 644.00000
178.00000 836.00000
279.00000 767.00000
338.00000 600.00000
326.00000 780.00000
417.00000 836.00000
404.00000 626.00000
129.00000 411.00000
468.00000 414.00000
273.00000 51.00000
262.00000 115.00000
254.00000 172.00000
246.00000 233.00000
228.00000 291.00000
210.00000 319.00000
168.00000 361.00000
325.00000 68.00000
336.00000 121.00000
344.00000 194.00000
358.00000 241.00000
372.00000 292.00000
392.00000 325.00000
439.00000 371.00000
ID=Pentaceratops
SCALE=0.109728

LM=59
381.00000 5.00000
371.00000 309.00000
335.00000 306.00000
231.00000 308.00000
229.00000 450.00000
56.00000 725.00000
364.00000 774.00000
639.00000 725.00000
476.00000 429.00000
402.00000 300.00000
497.00000 309.00000
192.00000 368.00000
118.00000 409.00000
90.00000 493.00000
74.00000 604.00000
90.00000 797.00000
178.00000 814.00000
238.00000 795.00000
306.00000 771.00000
428.00000 777.00000
498.00000 795.00000
536.00000 806.00000
571.00000 810.00000
631.00000 648.00000
622.00000 576.00000
603.00000 504.00000
567.00000 383.00000
310.00000 320.00000
291.00000 344.00000
275.00000 367.00000
251.00000 401.00000
421.00000 323.00000
433.00000 354.00000
446.00000 374.00000
459.00000 396.00000
301.00000 506.00000
216.00000 528.00000
166.00000 772.00000
331.00000 705.00000
401.00000 527.00000
406.00000 726.00000
572.00000 760.00000
490.00000 527.00000
209.00000 312.00000
522.00000 311.00000
354.00000 57.00000
339.00000 95.00000
331.00000 138.00000
301.00000 177.00000
285.00000 217.00000
266.00000 247.00000
240.00000 280.00000
405.00000 56.00000

413.00000 97.00000
421.00000 134.00000
429.00000 179.00000
447.00000 222.00000
458.00000 247.00000
470.00000 277.00000
ID=Chasmosaurus_belli
SCALE=0.073490
LM=59
339.00000 51.00000
347.00000 424.00000
307.00000 431.00000
243.00000 383.00000
240.00000 486.00000
174.00000 543.00000
348.00000 636.00000
517.00000 537.00000
456.00000 486.00000
392.00000 428.00000
444.00000 377.00000
232.00000 420.00000
201.00000 435.00000
179.00000 466.00000
171.00000 505.00000
183.00000 584.00000
196.00000 624.00000
216.00000 662.00000
264.00000 672.00000
388.00000 666.00000
449.00000 670.00000
489.00000 647.00000
505.00000 592.00000
523.00000 506.00000
517.00000 478.00000
499.00000 446.00000
466.00000 422.00000
287.00000 433.00000
273.00000 446.00000
265.00000 457.00000
253.00000 467.00000
408.00000 436.00000
419.00000 448.00000
430.00000 459.00000
444.00000 471.00000
276.00000 511.00000
229.00000 557.00000
270.00000 609.00000
304.00000 584.00000
386.00000 530.00000
422.00000 607.00000
462.00000 541.00000
421.00000 509.00000
218.00000 371.00000
468.00000 364.00000

317.00000 89.00000
308.00000 134.00000
301.00000 166.00000
295.00000 214.00000
284.00000 259.00000
274.00000 296.00000
259.00000 332.00000
357.00000 80.00000
368.00000 121.00000
378.00000 160.00000
386.00000 202.00000
398.00000 251.00000
408.00000 291.00000
426.00000 326.00000
ID=Styracosaurus
SCALE=0.096097
LM=59
231.00000 47.00000
248.00000 399.00000
211.00000 396.00000
153.00000 269.00000
150.00000 439.00000
130.00000 458.00000
249.00000 602.00000
367.00000 429.00000
346.00000 414.00000
273.00000 393.00000
335.00000 273.00000
163.00000 302.00000
164.00000 337.00000
140.00000 363.00000
125.00000 404.00000
140.00000 493.00000
150.00000 532.00000
166.00000 572.00000
191.00000 605.00000
291.00000 609.00000
325.00000 599.00000
349.00000 544.00000
360.00000 492.00000
370.00000 391.00000
365.00000 364.00000
347.00000 342.00000
332.00000 311.00000
195.00000 403.00000
185.00000 410.00000
175.00000 416.00000
159.00000 426.00000
286.00000 390.00000
297.00000 390.00000
312.00000 395.00000
334.00000 404.00000
207.00000 427.00000
169.00000 464.00000

198.00000 590.00000
223.00000 563.00000
284.00000 424.00000
281.00000 556.00000
311.00000 588.00000
332.00000 451.00000
119.00000 238.00000
366.00000 233.00000
210.00000 69.00000
206.00000 101.00000
204.00000 132.00000
192.00000 162.00000
180.00000 182.00000
167.00000 196.00000
162.00000 217.00000
251.00000 71.00000
263.00000 102.00000
276.00000 134.00000
288.00000 162.00000
307.00000 187.00000
324.00000 203.00000
331.00000 225.00000
ID=Diabloceratops
SCALE=0.080797
LM=59
233.00000 5.00000
235.00000 321.00000
216.00000 329.00000
136.00000 264.00000
113.00000 384.00000
87.00000 416.00000
234.00000 549.00000
379.00000 413.00000
351.00000 380.00000
260.00000 333.00000
330.00000 271.00000
136.00000 299.00000
116.00000 320.00000
94.00000 344.00000
85.00000 372.00000
91.00000 466.00000
106.00000 507.00000
130.00000 530.00000
169.00000 549.00000
278.00000 555.00000
307.00000 543.00000
340.00000 514.00000
367.00000 468.00000
382.00000 375.00000
375.00000 341.00000
362.00000 322.00000
341.00000 298.00000
185.00000 334.00000
157.00000 343.00000

138.00000 353.00000
124.00000 364.00000
286.00000 333.00000
305.00000 342.00000
320.00000 351.00000
337.00000 364.00000
190.00000 420.00000
124.00000 413.00000
154.00000 507.00000
199.00000 505.00000
272.00000 415.00000
253.00000 485.00000
314.00000 489.00000
347.00000 411.00000
125.00000 248.00000
346.00000 255.00000
217.00000 38.00000
211.00000 75.00000
203.00000 101.00000
187.00000 131.00000
183.00000 162.00000
178.00000 183.00000
167.00000 216.00000
245.00000 51.00000
248.00000 80.00000
257.00000 106.00000
271.00000 132.00000
282.00000 153.00000
287.00000 183.00000
297.00000 211.00000
ID=Achelousaurus
SCALE=0.111646
LM=59
339.00000 233.00000
346.00000 590.00000
308.00000 597.00000
170.00000 618.00000
115.00000 930.00000
64.00000 1001.00000
345.00000 1071.00000
626.00000 1003.00000
581.00000 947.00000
387.00000 598.00000
518.00000 619.00000
153.00000 709.00000
128.00000 770.00000
102.00000 846.00000
70.00000 919.00000
103.00000 1045.00000
162.00000 1067.00000
225.00000 1073.00000
283.00000 1076.00000
411.00000 1078.00000
461.00000 1075.00000

514.00000 1069.00000
583.00000 1046.00000
622.00000 924.00000
599.00000 863.00000
570.00000 799.00000
546.00000 728.00000
244.00000 624.00000
209.00000 695.00000
187.00000 764.00000
151.00000 838.00000
455.00000 635.00000
485.00000 709.00000
507.00000 776.00000
546.00000 847.00000
323.00000 812.00000
134.00000 922.00000
147.00000 1018.00000
303.00000 1024.00000
366.00000 812.00000
390.00000 1024.00000
546.00000 1020.00000
554.00000 921.00000
109.00000 628.00000
581.00000 619.00000
321.00000 303.00000
303.00000 356.00000
278.00000 410.00000
255.00000 466.00000
218.00000 509.00000
193.00000 551.00000
164.00000 586.00000
371.00000 316.00000
387.00000 369.00000
408.00000 407.00000
435.00000 457.00000
467.00000 502.00000
498.00000 548.00000
524.00000 587.00000
ID=Ukhaa_Tolgod_protoceratopsid
SCALE=0.030357
