# Supplementary material for: Modularity and heterochrony in the evolution of the ceratopsian dinosaur frill
Source: Ecol Evol. 2020 May 22;10(13):6288–309. doi: 10.1002/ece3.6361 (PMC7381594; doi:10.1002/ece3.6361)
Supplement: Supplementary file 3 — Appendix S3 [file ECE3-10-6288-s003.pdf]

Appendix 3. Landmark and semilandmark coordinates for the sample of *Liaoceratops yanzigouensis* specimens used in this study

LM=59
1025.00000 224.00000
1025.00000 1298.00000
958.00000 1358.00000
378.00000 1589.00000
581.00000 2277.00000
712.00000 2423.00000
1023.00000 2426.00000
1332.00000 2432.00000
1457.00000 2283.00000
1095.00000 1357.00000
1663.00000 1615.00000
444.00000 1769.00000
430.00000 1975.00000
451.00000 2173.00000
518.00000 2343.00000
772.00000 2434.00000
829.00000 2440.00000
903.00000 2443.00000
961.00000 2434.00000
1092.00000 2429.00000
1146.00000 2437.00000
1218.00000 2434.00000
1278.00000 2434.00000
1529.00000 2360.00000
1603.00000 2183.00000
1623.00000 1984.00000
1612.00000 1779.00000
743.00000 1449.00000
678.00000 1669.00000
618.00000 1909.00000
541.00000 2080.00000
1321.00000 1497.00000
1366.00000 1686.00000
1415.00000 1889.00000
1498.00000 2074.00000
923.00000 1940.00000
849.00000 1999.00000
829.00000 2114.00000
927.00000 2122.00000
1138.00000 1946.00000
1128.00000 2121.00000
1229.00000 2112.00000
1213.00000 1996.00000
249.00000 1637.00000
1798.00000 1637.00000
635.00000 924.00000
751.00000 511.00000
461.00000 1278.00000
828.00000 343.00000

722.00000 737.00000
547.00000 1102.00000
353.00000 1448.00000
1413.00000 928.00000
1292.00000 534.00000
1590.00000 1269.00000
1216.00000 333.00000
1325.00000 746.00000
1512.00000 1105.00000
1689.00000 1435.00000
ID=IVPPV12738
SCALE=0.034622
LM=59
286.00000 7.00000
286.00000 366.00000
239.00000 383.00000
100.00000 447.00000
139.00000 652.00000
155.00000 717.00000
284.00000 734.00000
415.00000 719.00000
434.00000 648.00000
324.00000 386.00000
473.00000 448.00000
107.00000 514.00000
101.00000 572.00000
98.00000 630.00000
117.00000 678.00000
177.00000 730.00000
202.00000 735.00000
228.00000 739.00000
256.00000 740.00000
314.00000 740.00000
344.00000 740.00000
368.00000 736.00000
394.00000 731.00000
456.00000 682.00000
475.00000 633.00000
470.00000 572.00000
466.00000 515.00000
188.00000 428.00000
162.00000 481.00000
146.00000 539.00000
128.00000 593.00000
387.00000 427.00000
413.00000 480.00000
425.00000 537.00000
441.00000 587.00000
251.00000 617.00000
192.00000 635.00000
179.00000 673.00000
254.00000 686.00000
316.00000 619.00000
313.00000 686.00000

388.00000 670.00000
377.00000 634.00000
51.00000 450.00000
523.00000 453.00000
178.00000 233.00000
225.00000 117.00000
109.00000 336.00000
250.00000 61.00000
206.00000 175.00000
144.00000 285.00000
78.00000 388.00000
399.00000 240.00000
352.00000 119.00000
466.00000 340.00000
325.00000 64.00000
372.00000 179.00000
432.00000 292.00000
496.00000 387.00000
ID=Uncatalogued#3
SCALE=1.383041
LM=59
1239.00000 247.00000
1069.00000 1798.00000
832.00000 1966.00000
243.00000 1882.00000
619.00000 2754.00000
593.00000 3155.00000
1117.00000 3194.00000
1662.00000 3101.00000
1638.00000 2718.00000
1395.00000 1956.00000
1990.00000 1842.00000
293.00000 2116.00000
372.00000 2376.00000
412.00000 2648.00000
467.00000 2871.00000
687.00000 3195.00000
788.00000 3210.00000
887.00000 3209.00000
1001.00000 3217.00000
1226.00000 3189.00000
1340.00000 3169.00000
1441.00000 3158.00000
1558.00000 3143.00000
1791.00000 2835.00000
1836.00000 2618.00000
1857.00000 2351.00000
1933.00000 2086.00000
650.00000 2014.00000
573.00000 2199.00000
573.00000 2382.00000
555.00000 2570.00000
1551.00000 2071.00000
1629.00000 2206.00000

1674.00000 2359.00000
1691.00000 2538.00000
865.00000 2524.00000
728.00000 2508.00000
751.00000 2907.00000
957.00000 2879.00000
1379.00000 2520.00000
1278.00000 2860.00000
1502.00000 2893.00000
1528.00000 2506.00000
190.00000 1525.00000
2075.00000 1564.00000
743.00000 927.00000
959.00000 577.00000
457.00000 1238.00000
1080.00000 412.00000
848.00000 742.00000
607.00000 1099.00000
316.00000 1373.00000
1665.00000 959.00000
1415.00000 643.00000
1839.00000 1273.00000
1316.00000 454.00000
1545.00000 805.00000
1749.00000 1107.00000
1940.00000 1427.00000
ID=Uncatalogued#1
SCALE=0.151313
LM=59
2602.00000 475.00000
2732.00000 2419.00000
2441.00000 2711.00000
1721.00000 2773.00000
1949.00000 4082.00000
1989.00000 4305.00000
2729.00000 4319.00000
3478.00000 4311.00000
3515.00000 4088.00000
3023.00000 2705.00000
3708.00000 2773.00000
1751.00000 3141.00000
1730.00000 3467.00000
1692.00000 3759.00000
1747.00000 4042.00000
2146.00000 4321.00000
2294.00000 4346.00000
2447.00000 4354.00000
2581.00000 4319.00000
2864.00000 4321.00000
3008.00000 4353.00000
3171.00000 4345.00000
3323.00000 4326.00000
3697.00000 4053.00000
3770.00000 3765.00000

3710.00000 3439.00000
3686.00000 3119.00000
2109.00000 2797.00000
1933.00000 3118.00000
1881.00000 3452.00000
1838.00000 3784.00000
3345.00000 2797.00000
3527.00000 3117.00000
3555.00000 3407.00000
3613.00000 3765.00000
2499.00000 3475.00000
2158.00000 3848.00000
2181.00000 3991.00000
2519.00000 3894.00000
2954.00000 3471.00000
2926.00000 3896.00000
3265.00000 3995.00000
3293.00000 3855.00000
1578.00000 2964.00000
3864.00000 2964.00000
2055.00000 1696.00000
2246.00000 1048.00000
1837.00000 2290.00000
2361.00000 742.00000
2161.00000 1333.00000
1971.00000 2018.00000
1709.00000 2607.00000
3368.00000 1739.00000
3061.00000 1097.00000
3634.00000 2330.00000
2905.00000 785.00000
3241.00000 1389.00000
3519.00000 2087.00000
3755.00000 2639.00000
ID=IVPPV12633
SCALE=0.136776
LM=59
512.00000 76.00000
513.00000 418.00000
463.00000 448.00000
328.00000 540.00000
360.00000 749.00000
390.00000 804.00000
514.00000 807.00000
632.00000 805.00000
664.00000 754.00000
690.00000 550.00000
555.00000 445.00000
334.00000 604.00000
331.00000 663.00000
330.00000 718.00000
336.00000 777.00000
412.00000 807.00000
436.00000 809.00000

463.00000 809.00000
489.00000 808.00000
539.00000 809.00000
564.00000 810.00000
589.00000 810.00000
611.00000 808.00000
687.00000 781.00000
700.00000 724.00000
692.00000 669.00000
690.00000 612.00000
431.00000 504.00000
403.00000 562.00000
367.00000 612.00000
357.00000 681.00000
592.00000 508.00000
620.00000 564.00000
657.00000 617.00000
666.00000 684.00000
482.00000 644.00000
442.00000 655.00000
431.00000 727.00000
501.00000 733.00000
540.00000 649.00000
525.00000 733.00000
592.00000 727.00000
583.00000 657.00000
283.00000 543.00000
738.00000 542.00000
394.00000 302.00000
430.00000 174.00000
336.00000 411.00000
463.00000 126.00000
415.00000 238.00000
365.00000 353.00000
312.00000 477.00000
633.00000 308.00000
597.00000 178.00000
693.00000 425.00000
564.00000 130.00000
610.00000 241.00000
664.00000 364.00000
715.00000 480.00000
ID=Uncatalogued#2
SCALE=1.315472
