# Supplementary material for: Modularity and heterochrony in the evolution of the ceratopsian dinosaur frill
Source: Ecol Evol. 2020 May 22;10(13):6288–309. doi: 10.1002/ece3.6361 (PMC7381594; doi:10.1002/ece3.6361)
Supplement: Supplementary file 4 — Appendix S4 [file ECE3-10-6288-s004.pdf]

Appendix 4. Landmark and semilandmark coordinates for the sample of Protoceratops andrewsi specimens used in this study

LM=59
540.00000 74.00000
538.00000 805.00000
479.00000 838.00000
191.00000 854.00000
276.00000 895.00000
208.00000 1164.00000
530.00000 1195.00000
852.00000 1177.00000
799.00000 907.00000
592.00000 839.00000
887.00000 859.00000
201.00000 915.00000
189.00000 983.00000
179.00000 1048.00000
174.00000 1114.00000
269.00000 1205.00000
336.00000 1213.00000
399.00000 1217.00000
467.00000 1207.00000
598.00000 1210.00000
673.00000 1220.00000
735.00000 1218.00000
803.00000 1210.00000
889.00000 1131.00000
891.00000 1056.00000
882.00000 988.00000
877.00000 926.00000
427.00000 820.00000
382.00000 807.00000
331.00000 816.00000
288.00000 846.00000
637.00000 817.00000
692.00000 808.00000
745.00000 824.00000
781.00000 861.00000
513.00000 986.00000
300.00000 886.00000
244.00000 1147.00000
469.00000 1187.00000
564.00000 986.00000
596.00000 1190.00000
820.00000 1161.00000
767.00000 886.00000
73.00000 881.00000
991.00000 892.00000
242.00000 459.00000
396.00000 275.00000
150.00000 651.00000
465.00000 181.00000

295.00000 345.00000
202.00000 553.00000
119.00000 763.00000
848.00000 476.00000
696.00000 281.00000
931.00000 669.00000
635.00000 180.00000
803.00000 361.00000
888.00000 574.00000
954.00000 778.00000
ID=MPC100/530b
SCALE=0.009508
LM=59
272.00000 8.00000
271.00000 320.00000
253.00000 339.00000
143.00000 303.00000
54.00000 418.00000
31.00000 435.00000
278.00000 577.00000
496.00000 497.00000
470.00000 455.00000
287.00000 339.00000
391.00000 298.00000
138.00000 345.00000
96.00000 365.00000
60.00000 372.00000
25.00000 389.00000
55.00000 476.00000
89.00000 501.00000
123.00000 529.00000
169.00000 561.00000
317.00000 571.00000
359.00000 568.00000
401.00000 559.00000
442.00000 546.00000
491.00000 413.00000
460.00000 398.00000
427.00000 378.00000
388.00000 352.00000
220.00000 353.00000
189.00000 373.00000
142.00000 382.00000
93.00000 395.00000
321.00000 362.00000
359.00000 383.00000
400.00000 408.00000
439.00000 431.00000
253.00000 449.00000
109.00000 421.00000
142.00000 507.00000
253.00000 516.00000
308.00000 435.00000
296.00000 526.00000

436.00000 513.00000
439.00000 441.00000
88.00000 286.00000
433.00000 301.00000
256.00000 51.00000
246.00000 84.00000
231.00000 119.00000
212.00000 153.00000
189.00000 182.00000
160.00000 211.00000
130.00000 235.00000
287.00000 56.00000
299.00000 95.00000
306.00000 135.00000
323.00000 166.00000
338.00000 193.00000
374.00000 218.00000
398.00000 253.00000
ID=AMNH6413
SCALE=0.154030
LM=59
228.00000 15.00000
212.00000 332.00000
193.00000 346.00000
88.00000 317.00000
42.00000 462.00000
14.00000 503.00000
208.00000 567.00000
404.00000 499.00000
383.00000 468.00000
226.00000 356.00000
331.00000 319.00000
96.00000 361.00000
74.00000 388.00000
45.00000 416.00000
27.00000 450.00000
31.00000 532.00000
53.00000 555.00000
101.00000 570.00000
157.00000 574.00000
243.00000 578.00000
299.00000 582.00000
334.00000 567.00000
375.00000 535.00000
409.00000 452.00000
383.00000 428.00000
359.00000 402.00000
331.00000 373.00000
163.00000 361.00000
137.00000 384.00000
105.00000 407.00000
70.00000 430.00000
260.00000 364.00000
290.00000 387.00000

316.00000 405.00000
346.00000 431.00000
181.00000 471.00000
85.00000 474.00000
108.00000 535.00000
181.00000 510.00000
221.00000 466.00000
230.00000 522.00000
277.00000 554.00000
324.00000 465.00000
45.00000 297.00000
372.00000 293.00000
212.00000 48.00000
192.00000 90.00000
177.00000 128.00000
150.00000 178.00000
129.00000 202.00000
96.00000 217.00000
71.00000 245.00000
240.00000 57.00000
250.00000 101.00000
259.00000 147.00000
280.00000 192.00000
308.00000 216.00000
340.00000 244.00000
364.00000 266.00000
ID=AMNH6414
SCALE=0.170795
LM=59
439.00000 16.00000
428.00000 438.00000
404.00000 467.00000
238.00000 455.00000
81.00000 755.00000
34.00000 802.00000
433.00000 929.00000
798.00000 763.00000
724.00000 697.00000
443.00000 465.00000
607.00000 443.00000
203.00000 543.00000
158.00000 601.00000
115.00000 654.00000
62.00000 711.00000
51.00000 850.00000
100.00000 890.00000
182.00000 924.00000
271.00000 927.00000
514.00000 927.00000
600.00000 913.00000
670.00000 894.00000
755.00000 859.00000
762.00000 676.00000
721.00000 630.00000

683.00000 584.00000
643.00000 523.00000
336.00000 499.00000
269.00000 550.00000
205.00000 621.00000
133.00000 688.00000
510.00000 488.00000
566.00000 537.00000
626.00000 593.00000
676.00000 642.00000
391.00000 678.00000
93.00000 796.00000
133.00000 862.00000
405.00000 848.00000
453.00000 672.00000
453.00000 872.00000
703.00000 831.00000
675.00000 672.00000
157.00000 456.00000
670.00000 458.00000
409.00000 83.00000
388.00000 137.00000
354.00000 209.00000
312.00000 265.00000
267.00000 319.00000
226.00000 363.00000
192.00000 408.00000
481.00000 75.00000
494.00000 144.00000
509.00000 204.00000
532.00000 249.00000
559.00000 287.00000
599.00000 331.00000
623.00000 389.00000
ID=FMNHP14045
SCALE=0.100877
LM=59
247.00000 19.00000
228.00000 360.00000
211.00000 360.00000
122.00000 325.00000
56.00000 460.00000
25.00000 501.00000
211.00000 580.00000
379.00000 511.00000
366.00000 476.00000
246.00000 355.00000
332.00000 325.00000
122.00000 364.00000
97.00000 396.00000
63.00000 426.00000
37.00000 454.00000
30.00000 538.00000
71.00000 562.00000

114.00000 579.00000
165.00000 582.00000
252.00000 585.00000
290.00000 579.00000
327.00000 564.00000
356.00000 538.00000
383.00000 452.00000
366.00000 429.00000
351.00000 404.00000
323.00000 363.00000
169.00000 379.00000
138.00000 401.00000
106.00000 420.00000
81.00000 437.00000
275.00000 374.00000
298.00000 396.00000
333.00000 424.00000
355.00000 447.00000
197.00000 456.00000
56.00000 485.00000
46.00000 514.00000
176.00000 556.00000
234.00000 464.00000
224.00000 551.00000
352.00000 516.00000
334.00000 464.00000
62.00000 310.00000
412.00000 311.00000
220.00000 62.00000
202.00000 109.00000
178.00000 142.00000
153.00000 172.00000
131.00000 204.00000
106.00000 241.00000
87.00000 275.00000
265.00000 66.00000
279.00000 99.00000
299.00000 135.00000
320.00000 157.00000
339.00000 189.00000
352.00000 222.00000
368.00000 262.00000
ID=AMNH6466
SCALE=0.175599
LM=59
514.00000 5.00000
517.00000 754.00000
480.00000 744.00000
242.00000 612.00000
91.00000 1015.00000
24.00000 1091.00000
522.00000 1284.00000
1004.00000 1045.00000
920.00000 1016.00000

546.00000 759.00000
767.00000 670.00000
261.00000 736.00000
178.00000 831.00000
96.00000 894.00000
33.00000 955.00000
78.00000 1161.00000
147.00000 1222.00000
231.00000 1268.00000
350.00000 1292.00000
630.00000 1298.00000
738.00000 1295.00000
849.00000 1245.00000
927.00000 1173.00000
949.00000 952.00000
888.00000 914.00000
808.00000 851.00000
738.00000 760.00000
368.00000 778.00000
292.00000 839.00000
228.00000 889.00000
147.00000 947.00000
625.00000 786.00000
696.00000 830.00000
756.00000 875.00000
830.00000 941.00000
450.00000 1045.00000
239.00000 955.00000
129.00000 1034.00000
463.00000 1218.00000
595.00000 1065.00000
604.00000 1229.00000
799.00000 1195.00000
886.00000 1013.00000
78.00000 606.00000
909.00000 664.00000
445.00000 116.00000
397.00000 193.00000
363.00000 267.00000
323.00000 351.00000
282.00000 437.00000
224.00000 486.00000
152.00000 530.00000
577.00000 89.00000
625.00000 172.00000
659.00000 251.00000
690.00000 311.00000
735.00000 398.00000
762.00000 483.00000
832.00000 575.00000
ID=AMNH6439
SCALE=0.059020
LM=59
310.00000 17.00000

318.00000 277.00000
296.00000 269.00000
180.00000 262.00000
87.00000 412.00000
37.00000 455.00000
338.00000 561.00000
552.00000 436.00000
499.00000 407.00000
340.00000 271.00000
420.00000 261.00000
166.00000 311.00000
127.00000 343.00000
81.00000 371.00000
44.00000 396.00000
78.00000 495.00000
135.00000 522.00000
184.00000 534.00000
248.00000 558.00000
384.00000 567.00000
439.00000 562.00000
486.00000 543.00000
537.00000 513.00000
519.00000 400.00000
486.00000 374.00000
455.00000 344.00000
429.00000 310.00000
253.00000 290.00000
220.00000 320.00000
184.00000 345.00000
142.00000 371.00000
383.00000 283.00000
407.00000 318.00000
438.00000 350.00000
468.00000 377.00000
311.00000 412.00000
99.00000 435.00000
102.00000 481.00000
329.00000 512.00000
341.00000 398.00000
384.00000 538.00000
510.00000 491.00000
459.00000 401.00000
66.00000 268.00000
495.00000 265.00000
266.00000 52.00000
243.00000 84.00000
219.00000 127.00000
201.00000 161.00000
178.00000 187.00000
154.00000 217.00000
123.00000 245.00000
332.00000 56.00000
342.00000 98.00000
365.00000 141.00000

386.00000 164.00000
404.00000 188.00000
416.00000 208.00000
435.00000 237.00000
ID=MPD530
SCALE=0.127902
LM=59
573.00000 35.00000
555.00000 880.00000
509.00000 878.00000
277.00000 830.00000
149.00000 1298.00000
52.00000 1450.00000
612.00000 1596.00000
1088.00000 1264.00000
1032.00000 1206.00000
596.00000 868.00000
837.00000 770.00000
270.00000 981.00000
195.00000 1103.00000
128.00000 1210.00000
38.00000 1332.00000
155.00000 1553.00000
270.00000 1605.00000
399.00000 1598.00000
497.00000 1596.00000
718.00000 1578.00000
838.00000 1562.00000
964.00000 1518.00000
1058.00000 1367.00000
1065.00000 1126.00000
1003.00000 1059.00000
918.00000 983.00000
835.00000 873.00000
438.00000 924.00000
360.00000 997.00000
286.00000 1075.00000
188.00000 1171.00000
693.00000 882.00000
782.00000 962.00000
853.00000 1011.00000
954.00000 1103.00000
534.00000 1201.00000
234.00000 1206.00000
303.00000 1512.00000
511.00000 1491.00000
615.00000 1202.00000
610.00000 1427.00000
908.00000 1456.00000
971.00000 1217.00000
148.00000 834.00000
954.00000 770.00000
521.00000 186.00000
488.00000 285.00000

458.00000 359.00000
406.00000 457.00000
335.00000 572.00000
287.00000 627.00000
220.00000 729.00000
596.00000 140.00000
612.00000 241.00000
628.00000 333.00000
679.00000 419.00000
725.00000 512.00000
812.00000 577.00000
876.00000 669.00000
ID=AMNH6425
SCALE=0.056471
LM=59
361.00000 17.00000
385.00000 484.00000
360.00000 501.00000
223.00000 478.00000
105.00000 713.00000
31.00000 774.00000
406.00000 989.00000
799.00000 785.00000
712.00000 722.00000
425.00000 503.00000
586.00000 492.00000
215.00000 536.00000
169.00000 605.00000
120.00000 648.00000
58.00000 698.00000
58.00000 828.00000
106.00000 874.00000
171.00000 931.00000
285.00000 970.00000
483.00000 999.00000
579.00000 992.00000
646.00000 957.00000
731.00000 888.00000
777.00000 714.00000
713.00000 663.00000
643.00000 623.00000
603.00000 564.00000
285.00000 508.00000
237.00000 563.00000
190.00000 613.00000
140.00000 662.00000
515.00000 533.00000
576.00000 590.00000
644.00000 644.00000
701.00000 696.00000
351.00000 754.00000
118.00000 776.00000
238.00000 912.00000
359.00000 924.00000

435.00000 785.00000
451.00000 948.00000
665.00000 883.00000
704.00000 764.00000
162.00000 465.00000
686.00000 465.00000
333.00000 76.00000
336.00000 143.00000
338.00000 207.00000
321.00000 272.00000
283.00000 321.00000
259.00000 357.00000
227.00000 406.00000
400.00000 79.00000
418.00000 146.00000
451.00000 202.00000
486.00000 263.00000
545.00000 313.00000
596.00000 359.00000
629.00000 409.00000
ID=MPC-D100/502
SCALE=0.072548
LM=59
253.00000 21.00000
256.00000 298.00000
233.00000 301.00000
142.00000 267.00000
60.00000 392.00000
8.00000 430.00000
271.00000 575.00000
487.00000 422.00000
420.00000 389.00000
276.00000 301.00000
370.00000 262.00000
134.00000 309.00000
94.00000 331.00000
62.00000 361.00000
45.00000 387.00000
24.00000 473.00000
66.00000 517.00000
112.00000 544.00000
184.00000 571.00000
319.00000 572.00000
363.00000 562.00000
415.00000 547.00000
467.00000 499.00000
471.00000 389.00000
445.00000 367.00000
414.00000 345.00000
365.00000 305.00000
189.00000 320.00000
155.00000 338.00000
123.00000 351.00000
93.00000 368.00000

314.00000 319.00000
343.00000 335.00000
366.00000 353.00000
396.00000 371.00000
208.00000 369.00000
61.00000 402.00000
47.00000 457.00000
247.00000 534.00000
276.00000 426.00000
281.00000 529.00000
422.00000 515.00000
431.00000 422.00000
84.00000 245.00000
442.00000 252.00000
230.00000 65.00000
221.00000 96.00000
210.00000 131.00000
193.00000 154.00000
172.00000 176.00000
151.00000 205.00000
141.00000 234.00000
276.00000 44.00000
288.00000 73.00000
304.00000 106.00000
321.00000 134.00000
343.00000 159.00000
367.00000 182.00000
393.00000 213.00000
ID=AMNH6409
SCALE=0.096038
LM=59
200.00000 13.00000
209.00000 320.00000
185.00000 323.00000
75.00000 310.00000
43.00000 482.00000
28.00000 561.00000
221.00000 588.00000
400.00000 529.00000
382.00000 491.00000
229.00000 324.00000
332.00000 307.00000
81.00000 342.00000
66.00000 375.00000
44.00000 418.00000
28.00000 460.00000
59.00000 578.00000
99.00000 590.00000
129.00000 600.00000
181.00000 597.00000
268.00000 592.00000
308.00000 589.00000
346.00000 576.00000
374.00000 560.00000

402.00000 476.00000
392.00000 435.00000
372.00000 396.00000
341.00000 359.00000
144.00000 320.00000
122.00000 352.00000
91.00000 394.00000
65.00000 431.00000
286.00000 332.00000
312.00000 368.00000
344.00000 409.00000
366.00000 445.00000
187.00000 470.00000
65.00000 475.00000
53.00000 546.00000
144.00000 577.00000
243.00000 475.00000
244.00000 555.00000
365.00000 540.00000
371.00000 482.00000
11.00000 329.00000
394.00000 324.00000
183.00000 78.00000
170.00000 116.00000
156.00000 154.00000
130.00000 188.00000
107.00000 217.00000
81.00000 243.00000
51.00000 284.00000
226.00000 62.00000
241.00000 101.00000
252.00000 144.00000
279.00000 181.00000
305.00000 210.00000
333.00000 241.00000
358.00000 279.00000
ID=AMNH6429
SCALE=0.128650
LM=59
175.00000 26.00000
177.00000 380.00000
140.00000 383.00000
57.00000 336.00000
42.00000 491.00000
48.00000 544.00000
177.00000 585.00000
304.00000 539.00000
290.00000 473.00000
206.00000 385.00000
297.00000 332.00000
60.00000 368.00000
60.00000 391.00000
54.00000 422.00000
38.00000 456.00000

63.00000 570.00000
85.00000 581.00000
112.00000 592.00000
146.00000 593.00000
205.00000 587.00000
232.00000 583.00000
259.00000 572.00000
286.00000 556.00000
312.00000 497.00000
299.00000 459.00000
290.00000 419.00000
289.00000 374.00000
111.00000 383.00000
90.00000 389.00000
70.00000 422.00000
57.00000 458.00000
234.00000 381.00000
261.00000 400.00000
272.00000 424.00000
280.00000 454.00000
147.00000 499.00000
64.00000 534.00000
104.00000 577.00000
153.00000 549.00000
197.00000 485.00000
213.00000 568.00000
257.00000 550.00000
279.00000 469.00000
16.00000 320.00000
346.00000 323.00000
153.00000 57.00000
141.00000 95.00000
122.00000 128.00000
105.00000 165.00000
86.00000 199.00000
65.00000 236.00000
49.00000 275.00000
198.00000 57.00000
219.00000 84.00000
241.00000 115.00000
258.00000 143.00000
282.00000 179.00000
296.00000 214.00000
314.00000 252.00000
ID=AMNH6419
SCALE=0.037355
LM=59
194.00000 10.00000
193.00000 292.00000
172.00000 303.00000
99.00000 305.00000
66.00000 452.00000
25.00000 507.00000
212.00000 588.00000

369.00000 503.00000
326.00000 454.00000
215.00000 297.00000
290.00000 296.00000
97.00000 348.00000
88.00000 387.00000
65.00000 426.00000
43.00000 460.00000
43.00000 539.00000
90.00000 562.00000
134.00000 577.00000
171.00000 581.00000
247.00000 589.00000
290.00000 587.00000
323.00000 577.00000
359.00000 546.00000
350.00000 460.00000
321.00000 422.00000
299.00000 383.00000
288.00000 344.00000
145.00000 307.00000
128.00000 338.00000
112.00000 363.00000
90.00000 404.00000
241.00000 314.00000
265.00000 346.00000
285.00000 372.00000
303.00000 409.00000
174.00000 471.00000
88.00000 447.00000
73.00000 527.00000
191.00000 555.00000
213.00000 483.00000
227.00000 557.00000
327.00000 552.00000
340.00000 482.00000
44.00000 322.00000
352.00000 315.00000
180.00000 58.00000
170.00000 101.00000
157.00000 141.00000
138.00000 178.00000
119.00000 213.00000
95.00000 256.00000
77.00000 286.00000
209.00000 47.00000
228.00000 93.00000
237.00000 130.00000
249.00000 158.00000
269.00000 187.00000
285.00000 223.00000
307.00000 264.00000
ID=AMNH6431
SCALE=0.073338

LM=59
213.00000 23.00000
233.00000 324.00000
215.00000 326.00000
117.00000 295.00000
68.00000 417.00000
26.00000 454.00000
252.00000 561.00000
452.00000 423.00000
408.00000 410.00000
254.00000 324.00000
340.00000 298.00000
129.00000 340.00000
90.00000 370.00000
47.00000 396.00000
20.00000 417.00000
49.00000 499.00000
89.00000 531.00000
135.00000 551.00000
189.00000 563.00000
294.00000 557.00000
338.00000 548.00000
387.00000 529.00000
428.00000 486.00000
432.00000 392.00000
401.00000 364.00000
354.00000 346.00000
329.00000 324.00000
180.00000 342.00000
157.00000 362.00000
137.00000 381.00000
106.00000 394.00000
288.00000 341.00000
309.00000 363.00000
344.00000 377.00000
383.00000 389.00000
209.00000 414.00000
68.00000 432.00000
79.00000 491.00000
234.00000 534.00000
262.00000 426.00000
269.00000 537.00000
410.00000 458.00000
387.00000 410.00000
40.00000 272.00000
424.00000 267.00000
183.00000 64.00000
164.00000 96.00000
143.00000 129.00000
122.00000 159.00000
105.00000 198.00000
84.00000 223.00000
62.00000 243.00000
239.00000 61.00000

258.00000 94.00000
286.00000 122.00000
310.00000 147.00000
329.00000 170.00000
364.00000 195.00000
394.00000 229.00000
ID=AMNH6444
SCALE=0.108562
LM=59
213.00000 3.00000
213.00000 355.00000
185.00000 361.00000
96.00000 325.00000
47.00000 469.00000
24.00000 497.00000
223.00000 583.00000
400.00000 488.00000
381.00000 472.00000
249.00000 357.00000
323.00000 323.00000
101.00000 354.00000
91.00000 388.00000
68.00000 419.00000
36.00000 443.00000
45.00000 538.00000
82.00000 565.00000
118.00000 578.00000
174.00000 587.00000
257.00000 583.00000
297.00000 579.00000
346.00000 569.00000
390.00000 532.00000
394.00000 454.00000
365.00000 428.00000
340.00000 402.00000
319.00000 368.00000
148.00000 363.00000
125.00000 393.00000
98.00000 422.00000
75.00000 442.00000
284.00000 368.00000
304.00000 400.00000
326.00000 421.00000
359.00000 445.00000
180.00000 471.00000
63.00000 489.00000
74.00000 542.00000
192.00000 571.00000
247.00000 491.00000
248.00000 564.00000
336.00000 556.00000
366.00000 497.00000
33.00000 297.00000
387.00000 292.00000

187.00000 36.00000
179.00000 77.00000
167.00000 115.00000
148.00000 152.00000
129.00000 189.00000
113.00000 210.00000
86.00000 249.00000
239.00000 41.00000
250.00000 79.00000
260.00000 125.00000
278.00000 165.00000
299.00000 195.00000
331.00000 230.00000
349.00000 264.00000
ID=AMNH6408.jpg
SCALE=0.112478
LM=59
513.00000 6.00000
501.00000 808.00000
445.00000 832.00000
253.00000 790.00000
142.00000 1151.00000
99.00000 1286.00000
498.00000 1394.00000
894.00000 1188.00000
914.00000 1268.00000
551.00000 839.00000
719.00000 807.00000
252.00000 891.00000
204.00000 982.00000
155.00000 1067.00000
102.00000 1162.00000
162.00000 1335.00000
223.00000 1359.00000
310.00000 1365.00000
393.00000 1391.00000
584.00000 1393.00000
701.00000 1382.00000
778.00000 1348.00000
851.00000 1304.00000
927.00000 1146.00000
851.00000 1082.00000
767.00000 998.00000
720.00000 919.00000
347.00000 848.00000
287.00000 927.00000
239.00000 993.00000
181.00000 1071.00000
651.00000 848.00000
677.00000 940.00000
751.00000 1019.00000
854.00000 1109.00000
427.00000 1182.00000
152.00000 1204.00000

162.00000 1291.00000
410.00000 1330.00000
572.00000 1187.00000
579.00000 1311.00000
814.00000 1278.00000
827.00000 1193.00000
139.00000 802.00000
815.00000 821.00000
463.00000 122.00000
435.00000 212.00000
429.00000 322.00000
382.00000 420.00000
323.00000 517.00000
276.00000 594.00000
245.00000 662.00000
558.00000 146.00000
584.00000 246.00000
590.00000 346.00000
625.00000 430.00000
667.00000 513.00000
706.00000 592.00000
737.00000 695.00000
ID=AMNH6433.jpg
SCALE=0.052702
