# Supplementary material for: Modularity and heterochrony in the evolution of the ceratopsian dinosaur frill
Source: Ecol Evol. 2020 May 22;10(13):6288–309. doi: 10.1002/ece3.6361 (PMC7381594; doi:10.1002/ece3.6361)
Supplement: Supplementary file 5 — Appendix S5 [file ECE3-10-6288-s005.pdf]

Appendix 5. Landmark and semilandmark coordinates for the sample of the undescribed Ukhaa Tolgod protoceratopsid specimens used in this study

LM=59
408.00000 160.00000
435.00000 623.00000
396.00000 675.00000
219.00000 673.00000
150.00000 916.00000
110.00000 1028.00000
441.00000 1136.00000
773.00000 1031.00000
724.00000 918.00000
472.00000 666.00000
678.00000 655.00000
188.00000 746.00000
141.00000 794.00000
115.00000 871.00000
80.00000 948.00000
170.00000 1063.00000
239.00000 1087.00000
298.00000 1115.00000
368.00000 1140.00000
518.00000 1133.00000
589.00000 1118.00000
654.00000 1092.00000
712.00000 1064.00000
806.00000 944.00000
770.00000 868.00000
739.00000 794.00000
694.00000 739.00000
322.00000 657.00000
266.00000 709.00000
231.00000 780.00000
195.00000 839.00000
553.00000 656.00000
604.00000 712.00000
649.00000 781.00000
694.00000 844.00000
388.00000 914.00000
228.00000 931.00000
194.00000 1003.00000
416.00000 1067.00000
477.00000 926.00000
467.00000 1075.00000
689.00000 995.00000
649.00000 930.00000
142.00000 676.00000
785.00000 677.00000
297.00000 443.00000
350.00000 312.00000
233.00000 565.00000

361.00000 232.00000
334.00000 383.00000
253.00000 498.00000
196.00000 623.00000
574.00000 426.00000
473.00000 298.00000
682.00000 536.00000
447.00000 227.00000
525.00000 363.00000
626.00000 476.00000
723.00000 601.00000
ID=IGM100/3658
SCALE=0.035618
LM=59
501.00000 112.00000
505.00000 655.00000
467.00000 721.00000
299.00000 710.00000
211.00000 999.00000
118.00000 1053.00000
532.00000 1245.00000
919.00000 1053.00000
803.00000 987.00000
542.00000 722.00000
724.00000 699.00000
305.00000 788.00000
273.00000 861.00000
222.00000 918.00000
164.00000 974.00000
164.00000 1139.00000
238.00000 1204.00000
339.00000 1238.00000
434.00000 1251.00000
637.00000 1238.00000
734.00000 1227.00000
824.00000 1193.00000
894.00000 1133.00000
869.00000 975.00000
811.00000 913.00000
752.00000 852.00000
712.00000 784.00000
394.00000 764.00000
361.00000 831.00000
323.00000 894.00000
268.00000 939.00000
619.00000 761.00000
657.00000 827.00000
692.00000 890.00000
746.00000 935.00000
473.00000 988.00000
203.00000 1040.00000
285.00000 1172.00000
496.00000 1145.00000
561.00000 991.00000

552.00000 1149.00000
786.00000 1151.00000
773.00000 972.00000
178.00000 697.00000
859.00000 647.00000
381.00000 429.00000
440.00000 280.00000
306.00000 571.00000
466.00000 200.00000
413.00000 350.00000
344.00000 505.00000
258.00000 638.00000
621.00000 436.00000
562.00000 277.00000
719.00000 560.00000
538.00000 201.00000
578.00000 357.00000
669.00000 500.00000
766.00000 622.00000
ID=GM100/3596
SCALE=0.045990
LM=59
477.00000 86.00000
463.00000 678.00000
242.00000 760.00000
96.00000 748.00000
168.00000 1056.00000
195.00000 1146.00000
467.00000 1148.00000
780.00000 1133.00000
793.00000 991.00000
712.00000 738.00000
850.00000 750.00000
108.00000 829.00000
113.00000 922.00000
121.00000 1011.00000
131.00000 1096.00000
253.00000 1152.00000
309.00000 1149.00000
364.00000 1148.00000
418.00000 1147.00000
530.00000 1148.00000
592.00000 1147.00000
655.00000 1143.00000
719.00000 1140.00000
827.00000 1081.00000
833.00000 997.00000
838.00000 913.00000
835.00000 831.00000
174.00000 763.00000
147.00000 824.00000
147.00000 899.00000
156.00000 985.00000
772.00000 738.00000

793.00000 794.00000
797.00000 860.00000
798.00000 926.00000
402.00000 981.00000
258.00000 994.00000
266.00000 1093.00000
390.00000 1089.00000
531.00000 965.00000
535.00000 1077.00000
689.00000 1058.00000
624.00000 954.00000
83.00000 721.00000
884.00000 760.00000
216.00000 366.00000
325.00000 208.00000
128.00000 543.00000
391.00000 137.00000
259.00000 283.00000
170.00000 455.00000
100.00000 632.00000
728.00000 391.00000
654.00000 206.00000
844.00000 566.00000
582.00000 141.00000
689.00000 299.00000
794.00000 475.00000
872.00000 661.00000
ID=IGM100/3655
SCALE=0.005367
LM=59
462.00000 70.00000
463.00000 555.00000
255.00000 627.00000
104.00000 670.00000
178.00000 983.00000
166.00000 1043.00000
462.00000 1052.00000
757.00000 1047.00000
754.00000 967.00000
686.00000 628.00000
815.00000 674.00000
110.00000 761.00000
105.00000 841.00000
96.00000 917.00000
104.00000 991.00000
222.00000 1050.00000
281.00000 1054.00000
342.00000 1057.00000
397.00000 1056.00000
524.00000 1057.00000
582.00000 1058.00000
638.00000 1055.00000
696.00000 1052.00000
823.00000 990.00000

823.00000 911.00000
816.00000 839.00000
810.00000 756.00000
174.00000 635.00000
151.00000 718.00000
160.00000 812.00000
156.00000 904.00000
759.00000 648.00000
774.00000 722.00000
771.00000 800.00000
770.00000 889.00000
391.00000 883.00000
279.00000 891.00000
280.00000 991.00000
393.00000 992.00000
530.00000 880.00000
528.00000 996.00000
650.00000 997.00000
648.00000 884.00000
68.00000 656.00000
855.00000 643.00000
237.00000 344.00000
327.00000 196.00000
147.00000 501.00000
385.00000 133.00000
276.00000 267.00000
190.00000 423.00000
105.00000 576.00000
697.00000 345.00000
602.00000 197.00000
782.00000 502.00000
538.00000 136.00000
650.00000 272.00000
737.00000 428.00000
817.00000 572.00000
ID=IGM100/1008
SCALE=0.007082
LM=59
473.00000 80.00000
480.00000 674.00000
445.00000 707.00000
263.00000 681.00000
244.00000 916.00000
143.00000 1085.00000
488.00000 1196.00000
834.00000 1089.00000
740.00000 932.00000
509.00000 709.00000
692.00000 698.00000
263.00000 764.00000
223.00000 842.00000
179.00000 925.00000
129.00000 993.00000
194.00000 1134.00000

279.00000 1167.00000
351.00000 1172.00000
419.00000 1182.00000
559.00000 1201.00000
633.00000 1185.00000
705.00000 1168.00000
777.00000 1148.00000
846.00000 994.00000
803.00000 931.00000
756.00000 857.00000
710.00000 785.00000
381.00000 705.00000
347.00000 752.00000
316.00000 803.00000
280.00000 856.00000
581.00000 709.00000
617.00000 769.00000
658.00000 829.00000
704.00000 878.00000
437.00000 964.00000
276.00000 907.00000
222.00000 1104.00000
425.00000 1140.00000
544.00000 968.00000
550.00000 1151.00000
801.00000 1087.00000
728.00000 943.00000
192.00000 675.00000
751.00000 706.00000
366.00000 409.00000
424.00000 258.00000
286.00000 544.00000
433.00000 178.00000
405.00000 340.00000
321.00000 477.00000
256.00000 609.00000
586.00000 423.00000
507.00000 274.00000
672.00000 557.00000
494.00000 184.00000
538.00000 348.00000
632.00000 493.00000
711.00000 626.00000
ID=IGM100/3501
SCALE=0.068789
LM=59
468.00000 69.00000
501.00000 731.00000
352.00000 794.00000
184.00000 746.00000
207.00000 1263.00000
188.00000 1378.00000
497.00000 1393.00000
795.00000 1375.00000

783.00000 1192.00000
649.00000 801.00000
799.00000 719.00000
176.00000 868.00000
182.00000 990.00000
157.00000 1118.00000
136.00000 1265.00000
245.00000 1402.00000
308.00000 1402.00000
372.00000 1400.00000
436.00000 1397.00000
554.00000 1392.00000
613.00000 1392.00000
675.00000 1393.00000
736.00000 1388.00000
831.00000 1246.00000
827.00000 1108.00000
800.00000 986.00000
804.00000 860.00000
249.00000 824.00000
224.00000 927.00000
218.00000 1046.00000
189.00000 1152.00000
739.00000 788.00000
758.00000 882.00000
758.00000 982.00000
783.00000 1076.00000
392.00000 1110.00000
264.00000 1120.00000
276.00000 1245.00000
399.00000 1242.00000
545.00000 1106.00000
554.00000 1238.00000
680.00000 1227.00000
690.00000 1090.00000
91.00000 817.00000
863.00000 776.00000
240.00000 423.00000
357.00000 242.00000
179.00000 612.00000
393.00000 144.00000
273.00000 321.00000
236.00000 522.00000
129.00000 702.00000
705.00000 388.00000
597.00000 241.00000
762.00000 589.00000
537.00000 164.00000
663.00000 310.00000
743.00000 488.00000
821.00000 679.00000
ID=IGM100/10020
SCALE=0.006657
LM=59

459.00000 162.00000
459.00000 678.00000
419.00000 712.00000
251.00000 745.00000
199.00000 1111.00000
147.00000 1190.00000
459.00000 1255.00000
769.00000 1184.00000
714.00000 1106.00000
492.00000 716.00000
664.00000 746.00000
231.00000 849.00000
199.00000 943.00000
163.00000 1035.00000
144.00000 1110.00000
198.00000 1239.00000
273.00000 1260.00000
339.00000 1262.00000
401.00000 1263.00000
534.00000 1263.00000
588.00000 1262.00000
654.00000 1258.00000
728.00000 1238.00000
759.00000 1062.00000
722.00000 962.00000
700.00000 886.00000
680.00000 833.00000
333.00000 736.00000
296.00000 828.00000
276.00000 921.00000
231.00000 1018.00000
605.00000 776.00000
632.00000 865.00000
650.00000 941.00000
688.00000 1023.00000
433.00000 954.00000
222.00000 1097.00000
246.00000 1203.00000
427.00000 1199.00000
484.00000 957.00000
491.00000 1203.00000
682.00000 1200.00000
688.00000 1087.00000
176.00000 752.00000
741.00000 752.00000
364.00000 483.00000
403.00000 325.00000
288.00000 631.00000
431.00000 245.00000
396.00000 407.00000
326.00000 560.00000
246.00000 699.00000
552.00000 479.00000
517.00000 326.00000

628.00000 626.00000
491.00000 246.00000
517.00000 408.00000
588.00000 560.00000
669.00000 698.00000
ID=IGM100/1246
SCALE=0.090578
LM=59
519.00000 107.00000
518.00000 573.00000
358.00000 639.00000
262.00000 645.00000
328.00000 858.00000
351.00000 953.00000
518.00000 956.00000
689.00000 952.00000
709.00000 859.00000
675.00000 640.00000
772.00000 647.00000
277.00000 718.00000
276.00000 795.00000
277.00000 864.00000
288.00000 931.00000
386.00000 955.00000
420.00000 957.00000
454.00000 957.00000
487.00000 957.00000
553.00000 957.00000
589.00000 958.00000
621.00000 958.00000
654.00000 956.00000
747.00000 933.00000
763.00000 863.00000
761.00000 791.00000
759.00000 721.00000
331.00000 678.00000
324.00000 724.00000
318.00000 767.00000
322.00000 813.00000
704.00000 681.00000
714.00000 726.00000
714.00000 770.00000
714.00000 814.00000
470.00000 770.00000
409.00000 777.00000
409.00000 840.00000
478.00000 845.00000
556.00000 758.00000
560.00000 846.00000
645.00000 845.00000
650.00000 761.00000
210.00000 686.00000
826.00000 683.00000
319.00000 384.00000

415.00000 229.00000
251.00000 539.00000
465.00000 169.00000
365.00000 309.00000
281.00000 456.00000
242.00000 616.00000
711.00000 380.00000
624.00000 224.00000
783.00000 538.00000
574.00000 169.00000
670.00000 305.00000
756.00000 456.00000
793.00000 609.00000
ID=IGM100/1013
SCALE=0.013223
LM=59
466.00000 31.00000
482.00000 766.00000
358.00000 819.00000
153.00000 779.00000
219.00000 1098.00000
157.00000 1229.00000
459.00000 1328.00000
740.00000 1265.00000
709.00000 1154.00000
579.00000 826.00000
733.00000 826.00000
202.00000 862.00000
189.00000 948.00000
177.00000 1042.00000
171.00000 1129.00000
203.00000 1276.00000
268.00000 1297.00000
329.00000 1318.00000
396.00000 1330.00000
517.00000 1343.00000
581.00000 1336.00000
639.00000 1321.00000
692.00000 1302.00000
765.00000 1173.00000
755.00000 1083.00000
738.00000 992.00000
724.00000 910.00000
291.00000 832.00000
261.00000 886.00000
244.00000 954.00000
233.00000 1027.00000
646.00000 863.00000
668.00000 929.00000
688.00000 1001.00000
706.00000 1070.00000
447.00000 1057.00000
270.00000 1106.00000
263.00000 1220.00000

433.00000 1233.00000
491.00000 1066.00000
491.00000 1232.00000
651.00000 1202.00000
632.00000 1050.00000
34.00000 769.00000
882.00000 776.00000
251.00000 417.00000
373.00000 249.00000
149.00000 600.00000
413.00000 142.00000
313.00000 343.00000
201.00000 507.00000
107.00000 690.00000
661.00000 419.00000
567.00000 233.00000
774.00000 587.00000
520.00000 134.00000
611.00000 320.00000
720.00000 508.00000
809.00000 678.00000
ID=IGM100/1019
SCALE=0.012596
