# Supplementary material for: Modularity and heterochrony in the evolution of the ceratopsian dinosaur frill
Source: Ecol Evol. 2020 May 22;10(13):6288–309. doi: 10.1002/ece3.6361 (PMC7381594; doi:10.1002/ece3.6361)
Supplement: Supplementary file 6 — Appendix S6 [file ECE3-10-6288-s006.pdf]

Appendix 6. Landmark and semilandmark coordinates for the sample of Triceratops horridus specimens used in this study

LM=51
344.00000 12.00000
327.00000 687.00000
286.00000 725.00000
182.00000 554.00000
135.00000 845.00000
79.00000 1020.00000
343.00000 1112.00000
614.00000 1003.00000
523.00000 821.00000
373.00000 723.00000
482.00000 562.00000
96.00000 618.00000
54.00000 729.00000
37.00000 827.00000
35.00000 930.00000
121.00000 1062.00000
171.00000 1092.00000
229.00000 1100.00000
282.00000 1113.00000
407.00000 1108.00000
473.00000 1101.00000
534.00000 1090.00000
581.00000 1055.00000
653.00000 903.00000
633.00000 807.00000
602.00000 714.00000
550.00000 632.00000
248.00000 743.00000
210.00000 759.00000
172.00000 779.00000
143.00000 807.00000
414.00000 725.00000
451.00000 744.00000
481.00000 765.00000
509.00000 791.00000
160.00000 510.00000
498.00000 520.00000
309.00000 87.00000
290.00000 167.00000
278.00000 249.00000
242.00000 352.00000
230.00000 402.00000
203.00000 439.00000
175.00000 473.00000
365.00000 79.00000
375.00000 162.00000
387.00000 225.00000
397.00000 272.00000
413.00000 326.00000

425.00000 393.00000
453.00000 466.00000
ID=BMRP2006.4.1.2
SCALE=0.068006
LM=51
319.00000 17.00000
333.00000 624.00000
277.00000 640.00000
185.00000 517.00000
178.00000 732.00000
91.00000 937.00000
342.00000 943.00000
584.00000 929.00000
476.00000 732.00000
391.00000 646.00000
471.00000 503.00000
130.00000 600.00000
44.00000 641.00000
20.00000 753.00000
35.00000 852.00000
145.00000 951.00000
195.00000 961.00000
243.00000 954.00000
294.00000 951.00000
391.00000 949.00000
436.00000 952.00000
484.00000 953.00000
534.00000 943.00000
639.00000 842.00000
643.00000 745.00000
615.00000 642.00000
541.00000 578.00000
251.00000 643.00000
235.00000 666.00000
216.00000 688.00000
195.00000 709.00000
418.00000 648.00000
430.00000 669.00000
446.00000 689.00000
463.00000 709.00000
159.00000 474.00000
502.00000 467.00000
281.00000 108.00000
276.00000 183.00000
264.00000 253.00000
247.00000 306.00000
232.00000 366.00000
213.00000 406.00000
190.00000 437.00000
353.00000 77.00000
359.00000 143.00000
371.00000 212.00000
387.00000 266.00000
404.00000 317.00000

419.00000 365.00000
450.00000 419.00000
ID=YPM1823
SCALE=0.088307
LM=51
432.00000 14.00000
434.00000 949.00000
365.00000 939.00000
217.00000 782.00000
260.00000 1147.00000
107.00000 1491.00000
444.00000 1539.00000
772.00000 1481.00000
636.00000 1172.00000
507.00000 943.00000
658.00000 772.00000
168.00000 929.00000
81.00000 1061.00000
49.00000 1211.00000
49.00000 1353.00000
170.00000 1538.00000
245.00000 1547.00000
317.00000 1553.00000
381.00000 1552.00000
511.00000 1550.00000
580.00000 1556.00000
655.00000 1542.00000
722.00000 1527.00000
832.00000 1350.00000
833.00000 1203.00000
807.00000 1060.00000
726.00000 923.00000
338.00000 979.00000
318.00000 1024.00000
300.00000 1062.00000
281.00000 1101.00000
550.00000 986.00000
577.00000 1033.00000
601.00000 1079.00000
622.00000 1125.00000
188.00000 765.00000
686.00000 759.00000
390.00000 126.00000
371.00000 218.00000
353.00000 313.00000
323.00000 420.00000
284.00000 521.00000
261.00000 597.00000
227.00000 677.00000
472.00000 126.00000
496.00000 220.00000
518.00000 324.00000
558.00000 437.00000
583.00000 521.00000

618.00000 601.00000
659.00000 675.00000
ID=YPM1821
SCALE=0.051231
LM=51
254.00000 5.00000
289.00000 656.00000
218.00000 688.00000
74.00000 485.00000
92.00000 786.00000
86.00000 925.00000
287.00000 967.00000
502.00000 919.00000
484.00000 752.00000
374.00000 678.00000
427.00000 471.00000
16.00000 589.00000
15.00000 703.00000
13.00000 791.00000
16.00000 859.00000
116.00000 960.00000
155.00000 971.00000
201.00000 982.00000
244.00000 979.00000
331.00000 969.00000
375.00000 965.00000
424.00000 954.00000
464.00000 941.00000
542.00000 843.00000
542.00000 740.00000
532.00000 651.00000
479.00000 570.00000
179.00000 695.00000
157.00000 716.00000
135.00000 738.00000
115.00000 759.00000
403.00000 690.00000
427.00000 702.00000
446.00000 714.00000
466.00000 729.00000
42.00000 421.00000
425.00000 380.00000
226.00000 48.00000
212.00000 108.00000
203.00000 174.00000
187.00000 248.00000
169.00000 301.00000
142.00000 353.00000
107.00000 389.00000
287.00000 59.00000
298.00000 123.00000
313.00000 175.00000
329.00000 231.00000
341.00000 282.00000

365.00000 325.00000
383.00000 368.00000
ID=FMNHP12003
SCALE=0.095406
LM=51
334.00000 34.00000
348.00000 605.00000
293.00000 623.00000
209.00000 509.00000
206.00000 711.00000
115.00000 849.00000
352.00000 935.00000
553.00000 854.00000
482.00000 723.00000
397.00000 622.00000
483.00000 500.00000
154.00000 565.00000
140.00000 640.00000
127.00000 712.00000
106.00000 780.00000
142.00000 898.00000
186.00000 931.00000
242.00000 949.00000
297.00000 948.00000
402.00000 940.00000
449.00000 940.00000
493.00000 922.00000
528.00000 892.00000
567.00000 776.00000
560.00000 708.00000
547.00000 629.00000
540.00000 558.00000
264.00000 622.00000
244.00000 639.00000
228.00000 660.00000
214.00000 682.00000
428.00000 620.00000
452.00000 641.00000
467.00000 666.00000
478.00000 691.00000
193.00000 470.00000
492.00000 458.00000
301.00000 112.00000
291.00000 173.00000
284.00000 244.00000
270.00000 303.00000
260.00000 352.00000
239.00000 400.00000
215.00000 440.00000
367.00000 85.00000
377.00000 130.00000
384.00000 202.00000
393.00000 254.00000
415.00000 305.00000

429.00000 358.00000
453.00000 407.00000
ID=AMNH5116
SCALE=0.109409
LM=51
422.00000 21.00000
401.00000 783.00000
294.00000 849.00000
166.00000 656.00000
184.00000 962.00000
96.00000 1202.00000
414.00000 1313.00000
704.00000 1192.00000
619.00000 983.00000
461.00000 831.00000
608.00000 653.00000
131.00000 754.00000
55.00000 857.00000
44.00000 1008.00000
57.00000 1128.00000
181.00000 1268.00000
257.00000 1287.00000
313.00000 1302.00000
366.00000 1306.00000
488.00000 1300.00000
551.00000 1282.00000
608.00000 1253.00000
653.00000 1226.00000
778.00000 1105.00000
765.00000 965.00000
728.00000 862.00000
662.00000 767.00000
263.00000 864.00000
242.00000 884.00000
226.00000 909.00000
208.00000 931.00000
493.00000 839.00000
534.00000 852.00000
566.00000 881.00000
587.00000 917.00000
126.00000 606.00000
629.00000 617.00000
381.00000 102.00000
350.00000 168.00000
342.00000 240.00000
324.00000 316.00000
288.00000 404.00000
257.00000 479.00000
198.00000 551.00000
471.00000 105.00000
482.00000 192.00000
485.00000 282.00000
496.00000 372.00000
509.00000 438.00000

537.00000 511.00000
579.00000 558.00000
ID=BMRPcastMOR2951

LM=35 (used only in the heterochrony analyses, in which the rostrum was not considered)
327.00000 74.00000
348.00000 625.00000
299.00000 581.00000
163.00000 632.00000
225.00000 844.00000
208.00000 973.00000
379.00000 989.00000
571.00000 938.00000
513.00000 787.00000
409.00000 578.00000
550.00000 607.00000
165.00000 703.00000
164.00000 780.00000
158.00000 851.00000
157.00000 924.00000
245.00000 980.00000
278.00000 986.00000
314.00000 988.00000
346.00000 990.00000
424.00000 990.00000
466.00000 986.00000
504.00000 976.00000
540.00000 960.00000
587.00000 881.00000
588.00000 812.00000
571.00000 744.00000
556.00000 681.00000
257.00000 601.00000
224.00000 645.00000
220.00000 698.00000
224.00000 750.00000
465.00000 580.00000
495.00000 622.00000
501.00000 677.00000
507.00000 732.00000
ID=UCMP154452
SCALE=0.026499

LM=35 (rostrum missing in this specimen; specimen used only in the heterochrony analyses)
349.00000 14.00000
350.00000 697.00000
232.00000 743.00000
160.00000 604.00000
88.00000 894.00000
37.00000 1009.00000
397.00000 1160.00000
740.00000 965.00000

697.00000 888.00000
479.00000 744.00000
569.00000 594.00000
93.00000 666.00000
67.00000 753.00000
46.00000 838.00000
5.00000 928.00000
94.00000 1055.00000
163.00000 1105.00000
236.00000 1137.00000
316.00000 1156.00000
493.00000 1161.00000
573.00000 1133.00000
642.00000 1097.00000
711.00000 1039.00000
746.00000 871.00000
705.00000 801.00000
668.00000 736.00000
637.00000 663.00000
185.00000 747.00000
148.00000 784.00000
125.00000 818.00000
104.00000 860.00000
531.00000 758.00000
585.00000 775.00000
623.00000 810.00000
667.00000 854.00000
ID=MPMVP6841
SCALE=0.084224
