# Supplementary material for: Modularity and heterochrony in the evolution of the ceratopsian dinosaur frill
Source: Ecol Evol. 2020 May 22;10(13):6288–309. doi: 10.1002/ece3.6361 (PMC7381594; doi:10.1002/ece3.6361)
Supplement: Supplementary file 7 — Appendix S7 [file ECE3-10-6288-s007.pdf]

Taxon	**Abbreviation**	**Maximum Age (Ma)**
Achelousaurus horneri	AchC	74,50
Arrhinoceratops brachyops	ArrH	71,20
Centrosaurus apertus	CenC	77,00
Chasmosaurus belli	ChbH	76,00
Diabloceratops eatoni	DblC	79,60
Einiosaurus procurvicornis	EinC	74,60
Kosmoceratops richardsoni	KosH	76,00
Liaoceratops yanzigouensis	LiaN	125,00
Mojoceratops perifania	MojH	75,50
Nasutoceratops titusi	NasC	75,90
Pachyrhinosaurus lakustai	PacC	73,20
Pentaceratops sternbergii	PenH	74,00
Protoceratops andrewsi	PrtN	75,00
Psittacosaurus lujiatunensis	PslB	132,90
Psittacosaurus mongoliensis	PsmB	125,50
Regaliceratops peterhewsi	RegH	68,50
Styracosaurus albertensis	StyC	76,50
Ukhaa Tolgod protoceratopsid	Ukt	75,00
Triceratops horridus	TriH	66,50
Utahceratops gettyi	UtaH	76,00
Vagaceratops irvinensis	VagH	75,20
Yinlong downsi	YinB	163,50

Minimum Age (Ma)	Source
74,00	Evans and Ryan 2015, Fig. 14
70,60	Mallon et al. 2014, Fig. 2
76,40	Evans and Ryan 2015, Fig. 14
75,50	Mallon et al. 2012, Fig . 1; Gates et al. 2012
79,60	Kirkland and DeBlieux 2010
74,10	Evans and Ryan 2015, Fig. 14
76,00	Gates et al. 2012
125,00	You et al. 2007
75,00	Longrich 2010
75,50	Sampson et al. 2013
73,20	Currie et al. 2008
73,50	Evans and Ryan 2015, Fig. 14
71,00	Dashzeveg et al. 2005; Tsogtbaatar et al. 2014
129,40	Zhou et al. 2006
112,60	http://fossilworks.org/bridge.pl?a=taxonInfo&taxon_no=52831
67,50	Brown and Henderson 2015
75,50	Evans and Ryan 2015, Fig. 14
71,00	Dashzeveg et al 2005, Tsogtbaatar et al 2014
66,50	Gates et al. 2012
76,00	Gates et al. 2012
75,20	Holmes et al. 2001, Fig. 11; Mallon et al 2012, Fig . 1
157,30	Xu et al. 2006
