# Supplementary material for: Modularity and heterochrony in the evolution of the ceratopsian dinosaur frill
Source: Ecol Evol. 2020 May 22;10(13):6288–309. doi: 10.1002/ece3.6361 (PMC7381594; doi:10.1002/ece3.6361)
Supplement: Supplementary file 8 — Appendix S8 [file ECE3-10-6288-s008.pdf]

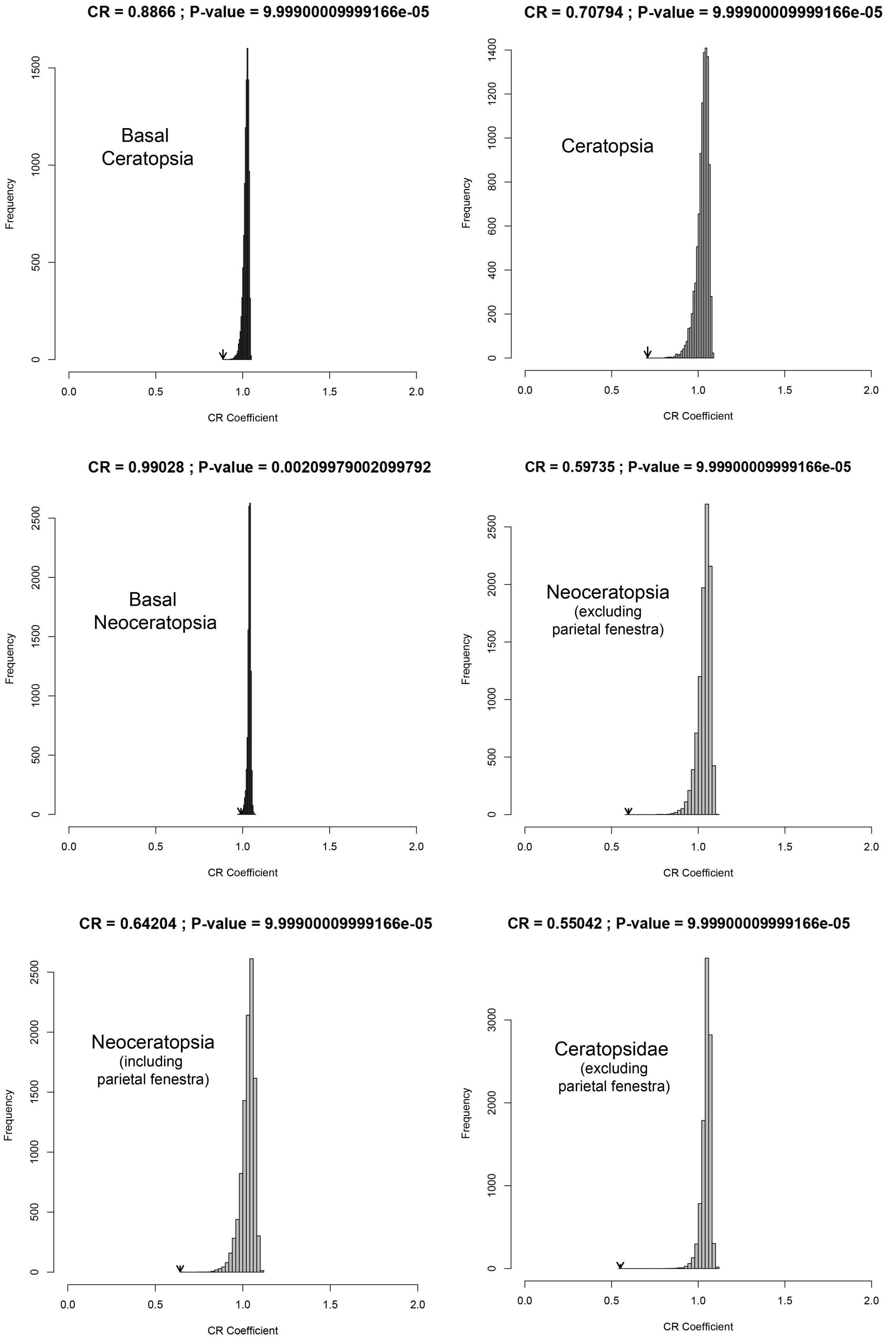

CR = 0.8866 ; P-value = 9.99900009999166e-05
Basal Ceratopsia
CR = 0.70794 ; P-value = 9.99900009999166e-05
Ceratopsia
CR = 0.99028 ; P-value = 0.00209979002099792
Basal Neoceratopsia
CR = 0.59735 ; P-value = 9.99900009999166e-05
Neoceratopsia (excluding parietal fenestra)
CR = 0.64204 ; P-value = 9.99900009999166e-05
Neoceratopsia (including parietal fenestra)
CR = 0.55042 ; P-value = 9.99900009999166e-05
Ceratopsidae (excluding parietal fenestra)
Frequency
CR Coefficient
0.0
0.5
1.0
1.5
2.0

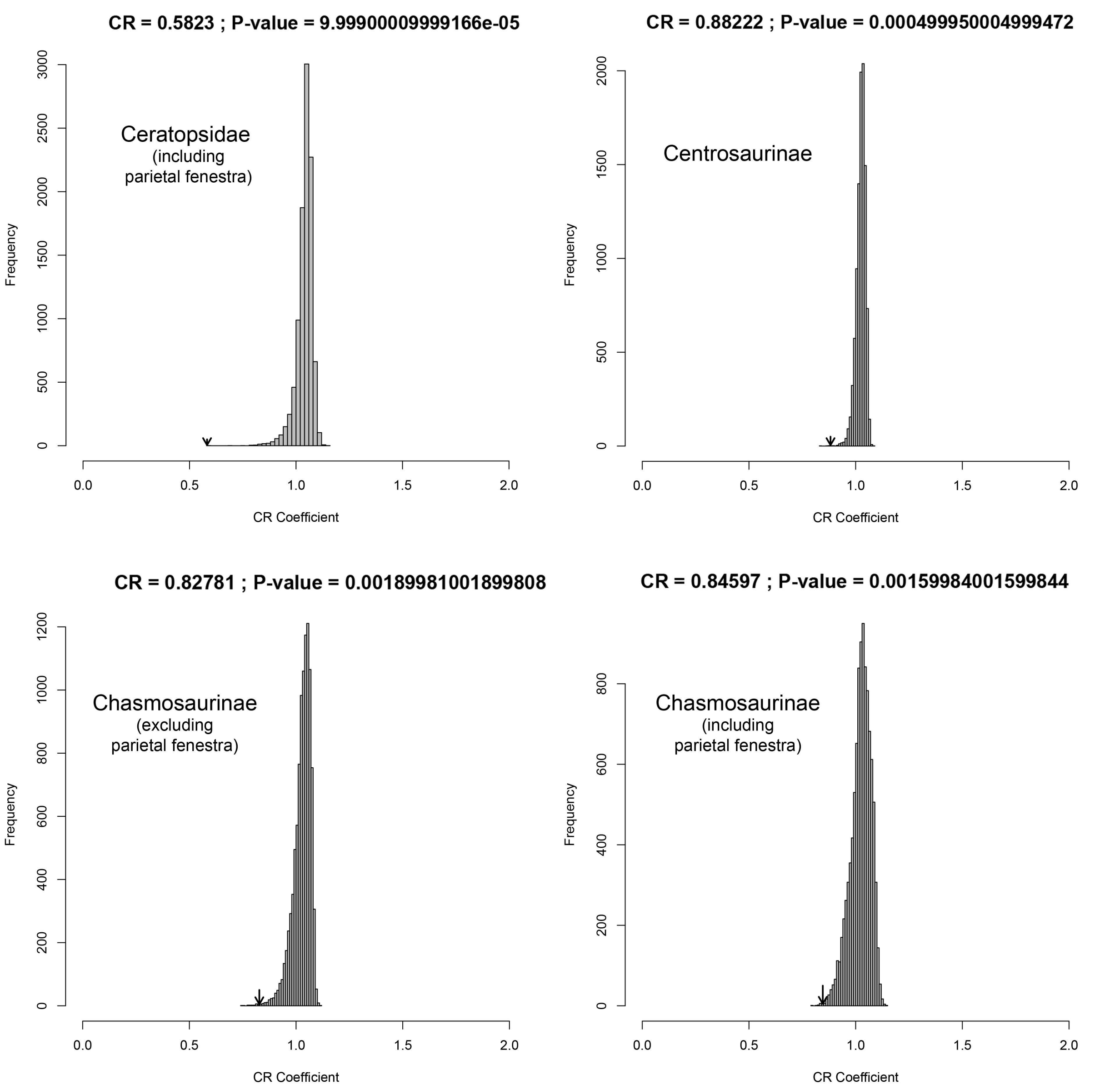
CR = 0.5823 ; P-value = 9.99900009999166e-05
Ceratopsidae
(including
parietal fenestra)
Frequency
0
500
1000
1500
2000
2500
3000
0.0
0.5
1.0
1.5
2.0
CR Coefficient
CR = 0.88222 ; P-value = 0.000499950004999472
Centrosaurinae
Frequency
0
500
1000
1500
2000
0.0
0.5
1.0
1.5
2.0
CR Coefficient
CR = 0.82781 ; P-value = 0.00189981001899808
Chasmosaurinae
(excluding
parietal fenestra)
Frequency
0
200
400
600
800
1000
1200
0.0
0.5
1.0
1.5
2.0
CR Coefficient
CR = 0.84597 ; P-value = 0.00159984001599844
Chasmosaurinae
(including
parietal fenestra)
Frequency
0
200
400
600
800
0.0
0.5
1.0
1.5
2.0
CR Coefficient

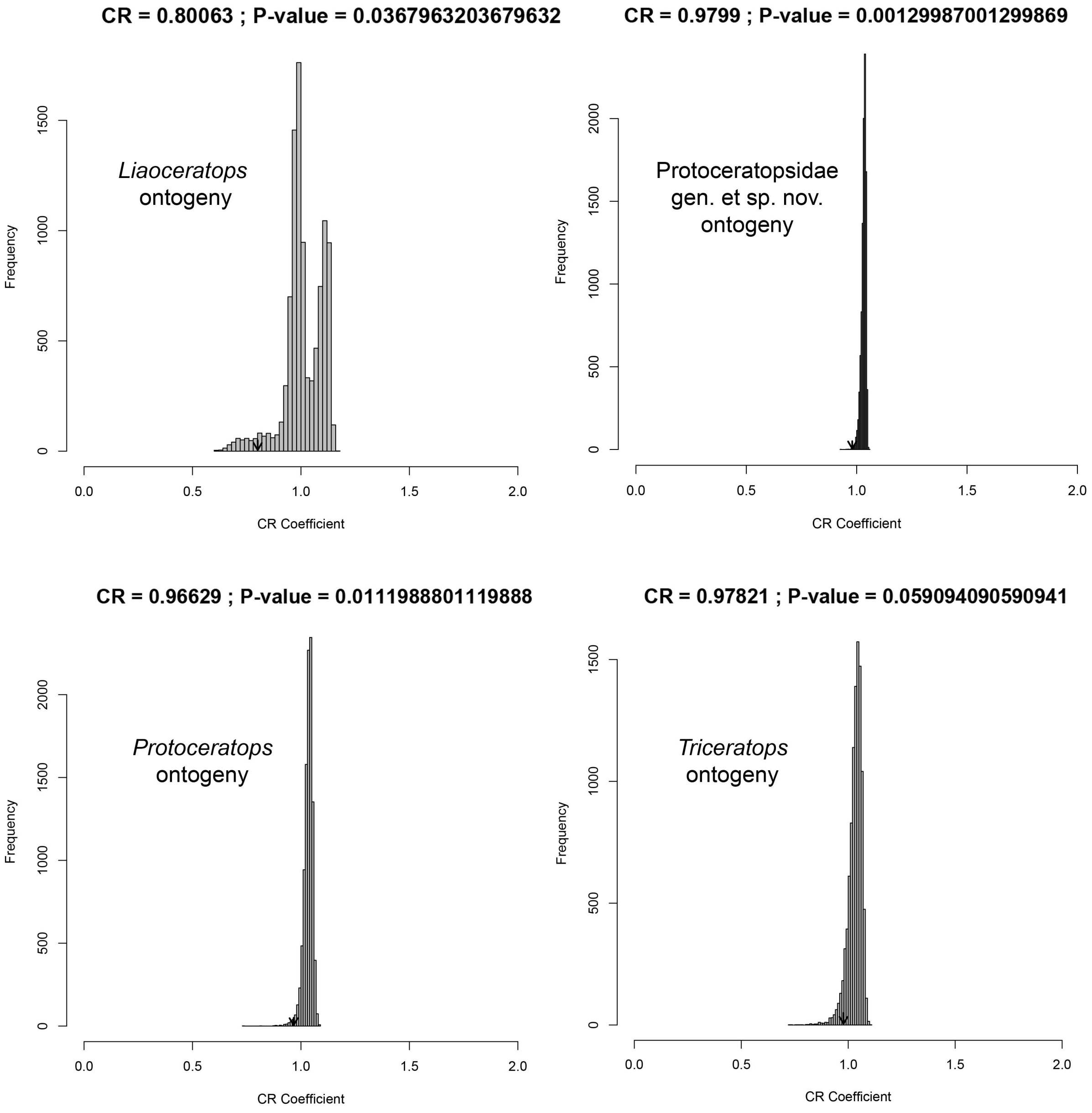
CR = 0.80063 ; P-value = 0.0367963203679632
Liaoceratops ontogeny
Frequency
CR Coefficient
CR = 0.9799 ; P-value = 0.00129987001299869
Protoceratopsidae gen. et sp. nov. ontogeny
Frequency
CR Coefficient
CR = 0.96629 ; P-value = 0.0111988801119888
Protoceratops ontogeny
Frequency
CR Coefficient
CR = 0.97821 ; P-value = 0.059094090590941
Triceratops ontogeny
Frequency
CR Coefficient
