# Supplementary material for: Modularity and heterochrony in the evolution of the ceratopsian dinosaur frill
Source: Ecol Evol. 2020 May 22;10(13):6288–309. doi: 10.1002/ece3.6361 (PMC7381594; doi:10.1002/ece3.6361)
Supplement: Supplementary file 9 — Appendix S9 [file ECE3-10-6288-s009.pdf]

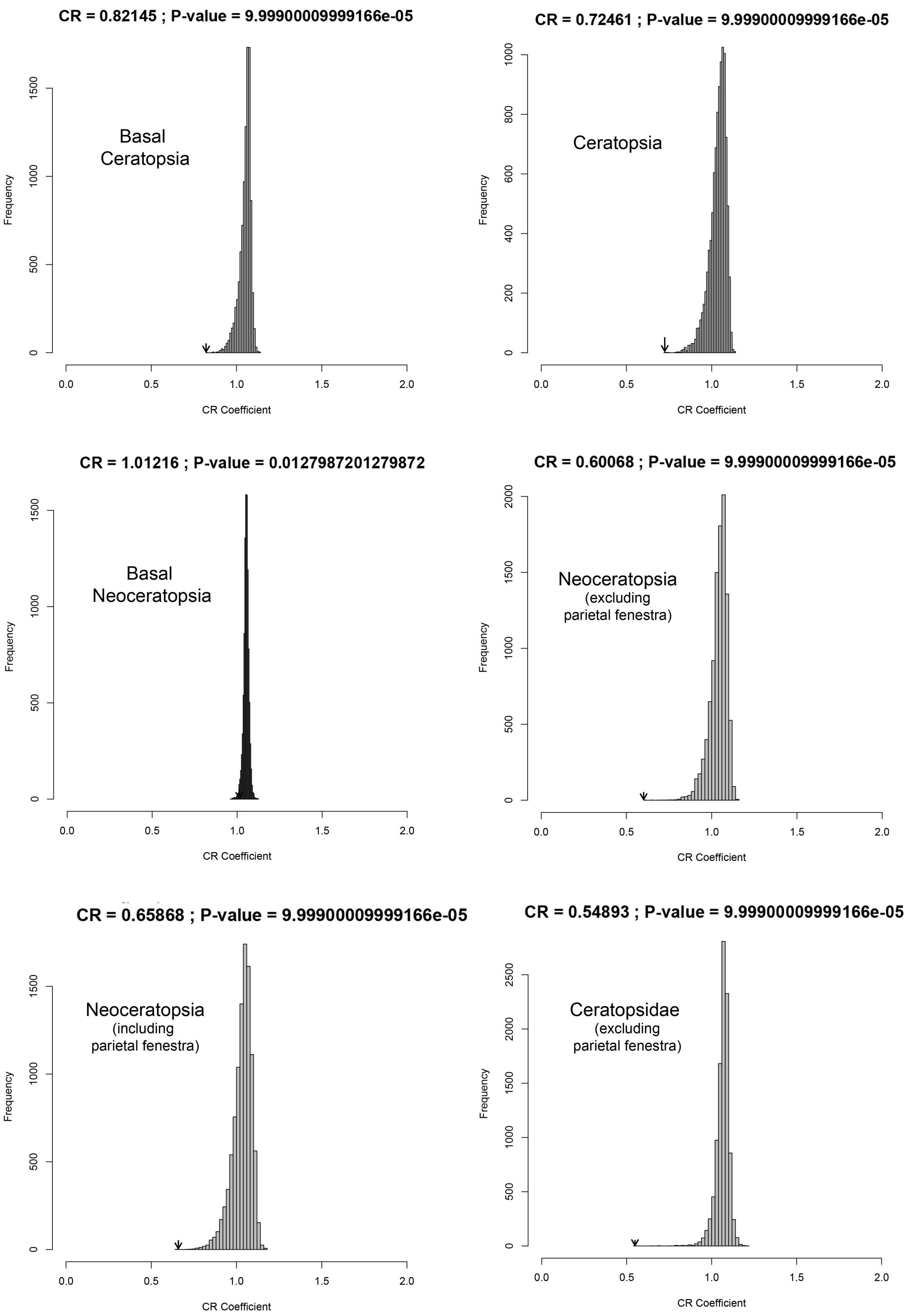
CR = 0.82145 ; P-value = 9.99900009999166e-05
Basal Ceratopsia
Frequency
CR Coefficient
0.0
0.5
1.0
1.5
2.0
0
500
1000
1500
CR = 0.72461 ; P-value = 9.99900009999166e-05
Ceratopsia
Frequency
CR Coefficient
0
200
400
600
800
1000
CR = 1.01216 ; P-value = 0.0127987201279872
Basal Neoceratopsia
Frequency
CR Coefficient
CR = 0.60068 ; P-value = 9.99900009999166e-05
Neoceratopsia (excluding parietal fenestra)
Frequency
CR Coefficient
2000
CR = 0.65868 ; P-value = 9.99900009999166e-05
Neoceratopsia (including parietal fenestra)
Frequency
CR Coefficient
CR = 0.54893 ; P-value = 9.99900009999166e-05
Ceratopsidae (excluding parietal fenestra)
Frequency
CR Coefficient
2500

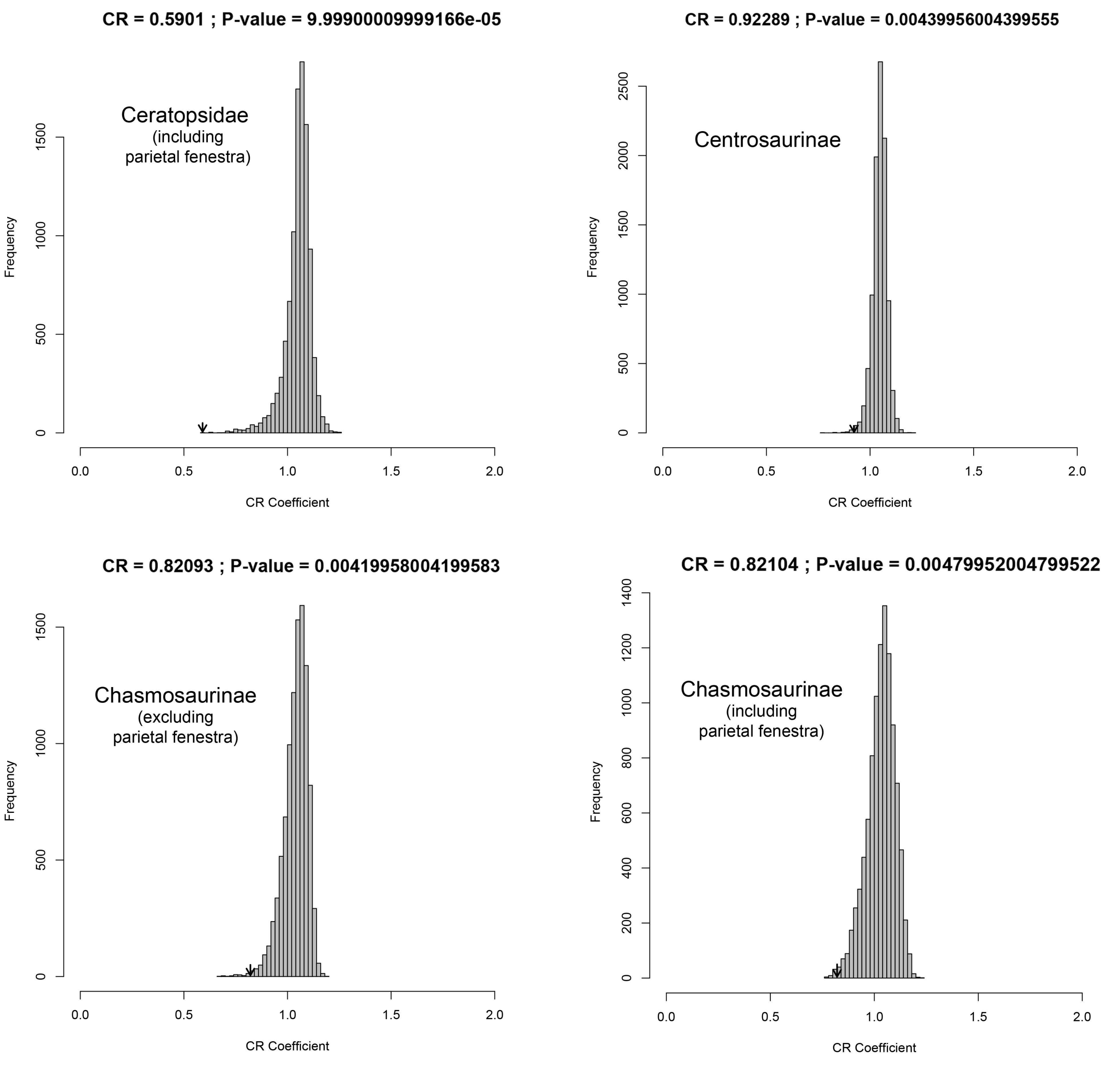
CR = 0.5901 ; P-value = 9.99900009999166e-05
Ceratopsidae
(including
parietal fenestra)
Frequency
CR Coefficient
CR = 0.92289 ; P-value = 0.00439956004399555
Centrosaurinae
Frequency
CR Coefficient
CR = 0.82093 ; P-value = 0.00419958004199583
Chasmosaurinae
(excluding
parietal fenestra)
Frequency
CR Coefficient
CR = 0.82104 ; P-value = 0.00479952004799522
Chasmosaurinae
(including
parietal fenestra)
Frequency
CR Coefficient

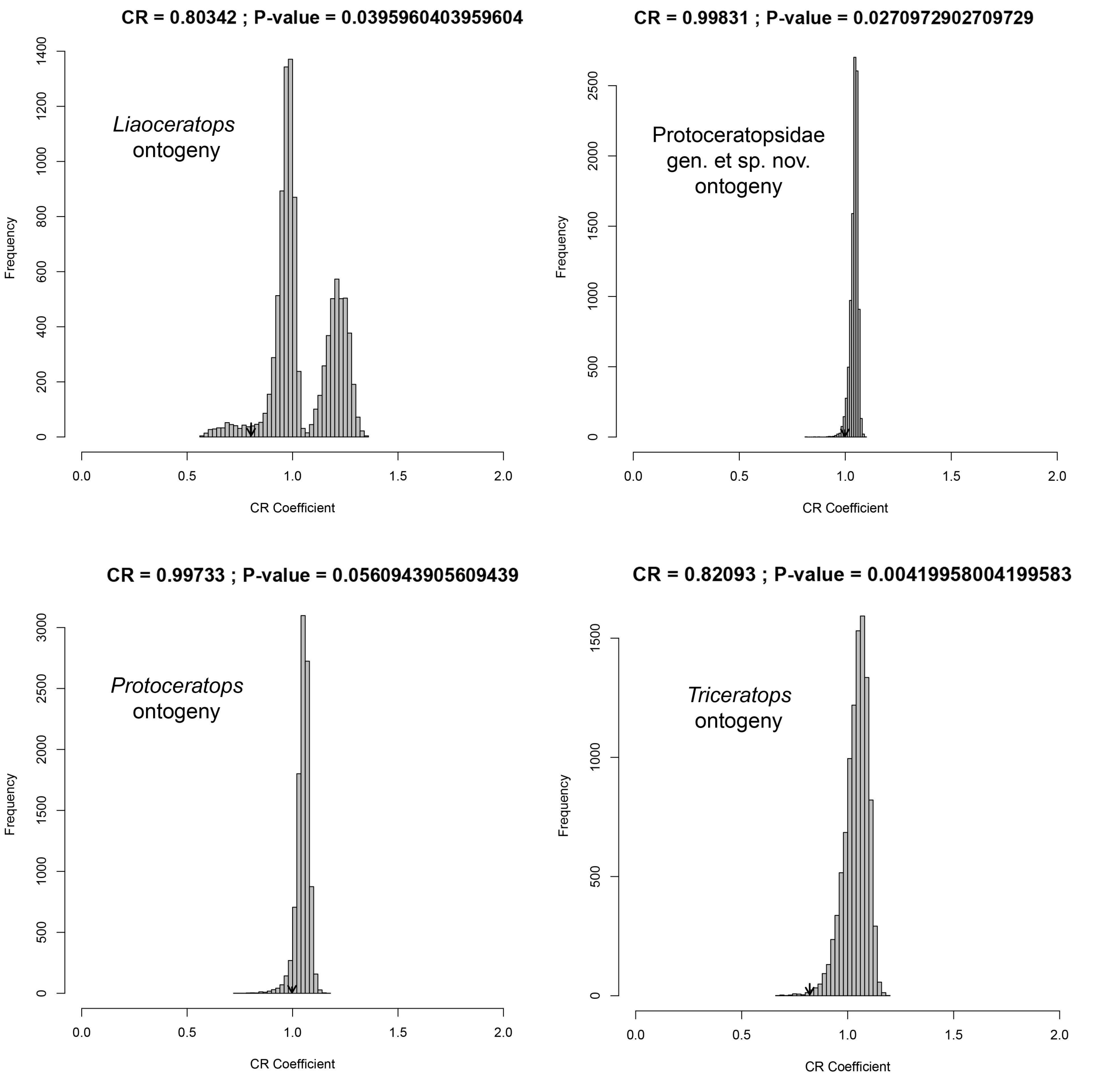
CR = 0.80342 ; P-value = 0.0395960403959604
Liaoceratops ontogeny
Frequency
CR Coefficient
CR = 0.99831 ; P-value = 0.0270972902709729
Protoceratopsidae gen. et sp. nov. ontogeny
Frequency
CR Coefficient
CR = 0.99733 ; P-value = 0.0560943905609439
Protoceratops ontogeny
Frequency
CR Coefficient
CR = 0.82093 ; P-value = 0.00419958004199583
Triceratops ontogeny
Frequency
CR Coefficient
