# Supplementary material for: Modularity and heterochrony in the evolution of the ceratopsian dinosaur frill
Source: Ecol Evol. 2020 May 22;10(13):6288–309. doi: 10.1002/ece3.6361 (PMC7381594; doi:10.1002/ece3.6361)
Supplement: Supplementary file 10 — Appendix S10 [file ECE3-10-6288-s010.pdf]

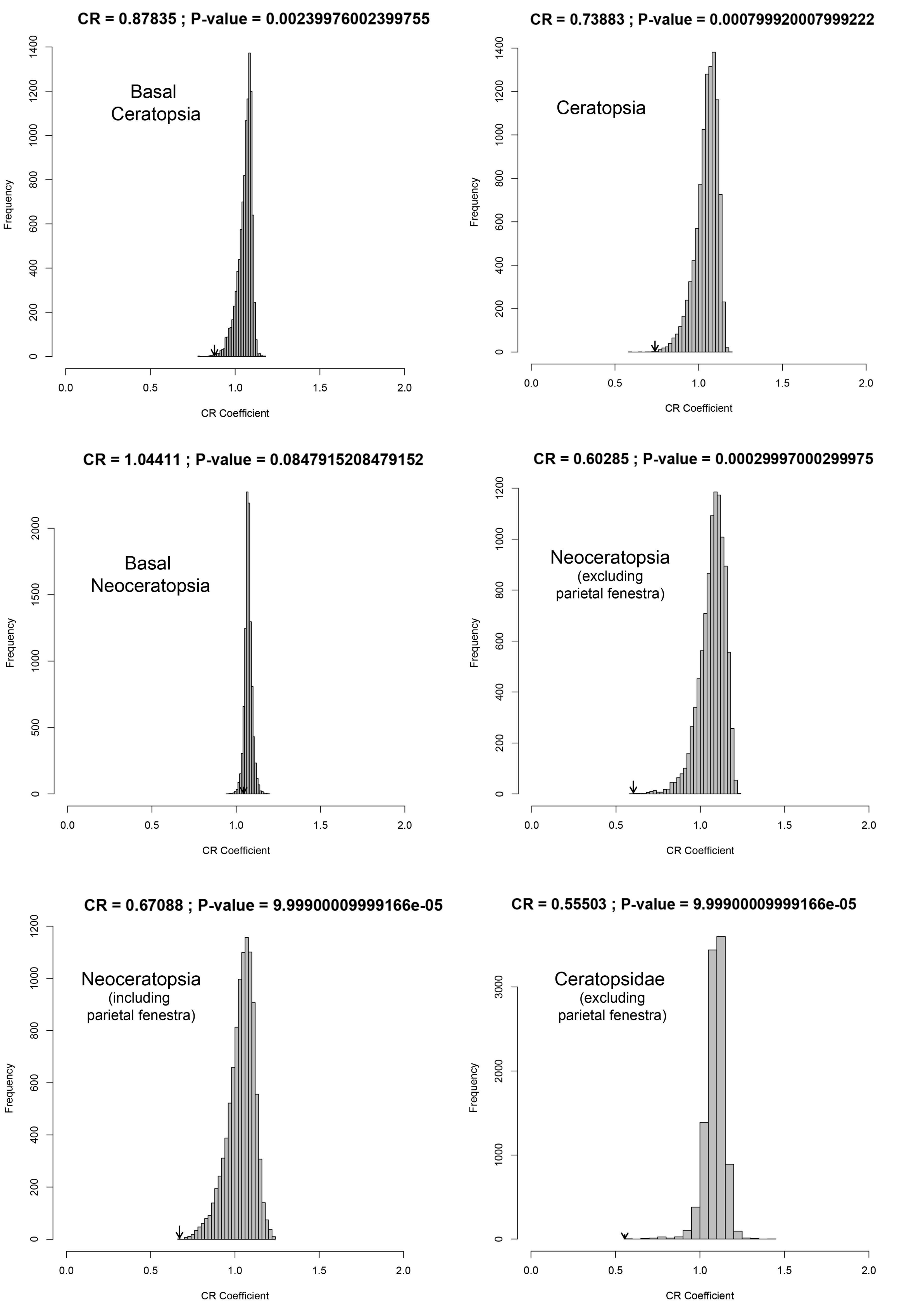
CR = 0.87835 ; P-value = 0.00239976002399755
Basal Ceratopsia
Frequency
CR Coefficient
CR = 0.73883 ; P-value = 0.000799920007999222
Ceratopsia
Frequency
CR Coefficient
CR = 1.04411 ; P-value = 0.0847915208479152
Basal Neoceratopsia
Frequency
CR Coefficient
CR = 0.60285 ; P-value = 0.00029997000299975
Neoceratopsia (excluding parietal fenestra)
Frequency
CR Coefficient
CR = 0.67088 ; P-value = 9.99900009999166e-05
Neoceratopsia (including parietal fenestra)
Frequency
CR Coefficient
CR = 0.55503 ; P-value = 9.99900009999166e-05
Ceratopsidae (excluding parietal fenestra)
Frequency
CR Coefficient

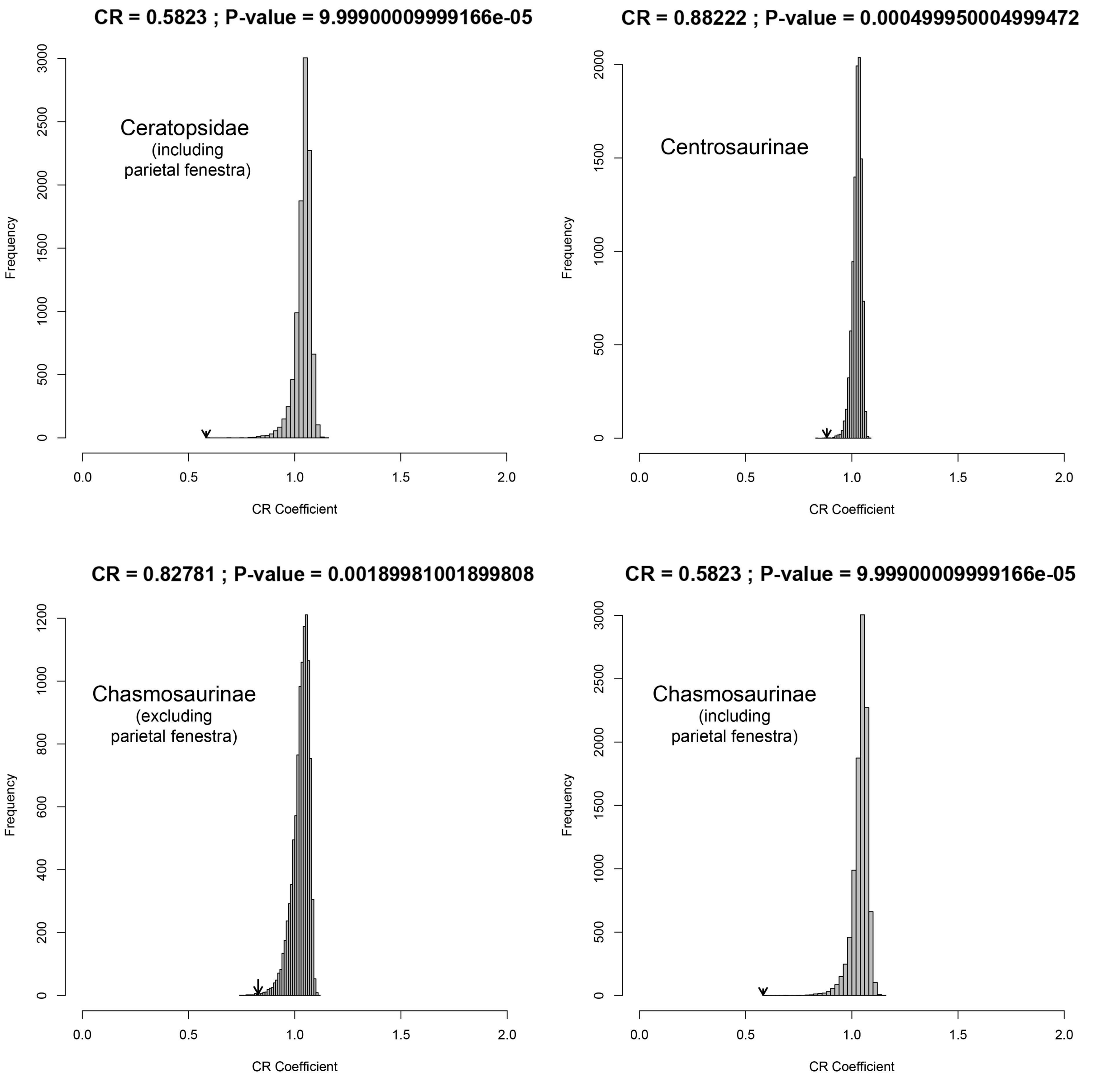
CR = 0.5823 ; P-value = 9.99900009999166e-05
Ceratopsidae
(including
parietal fenestra)
Frequency
CR Coefficient
CR = 0.88222 ; P-value = 0.000499950004999472
Centrosaurinae
Frequency
CR Coefficient
CR = 0.82781 ; P-value = 0.00189981001899808
Chasmosaurinae
(excluding
parietal fenestra)
Frequency
CR Coefficient
CR = 0.5823 ; P-value = 9.99900009999166e-05
Chasmosaurinae
(including
parietal fenestra)
Frequency
CR Coefficient

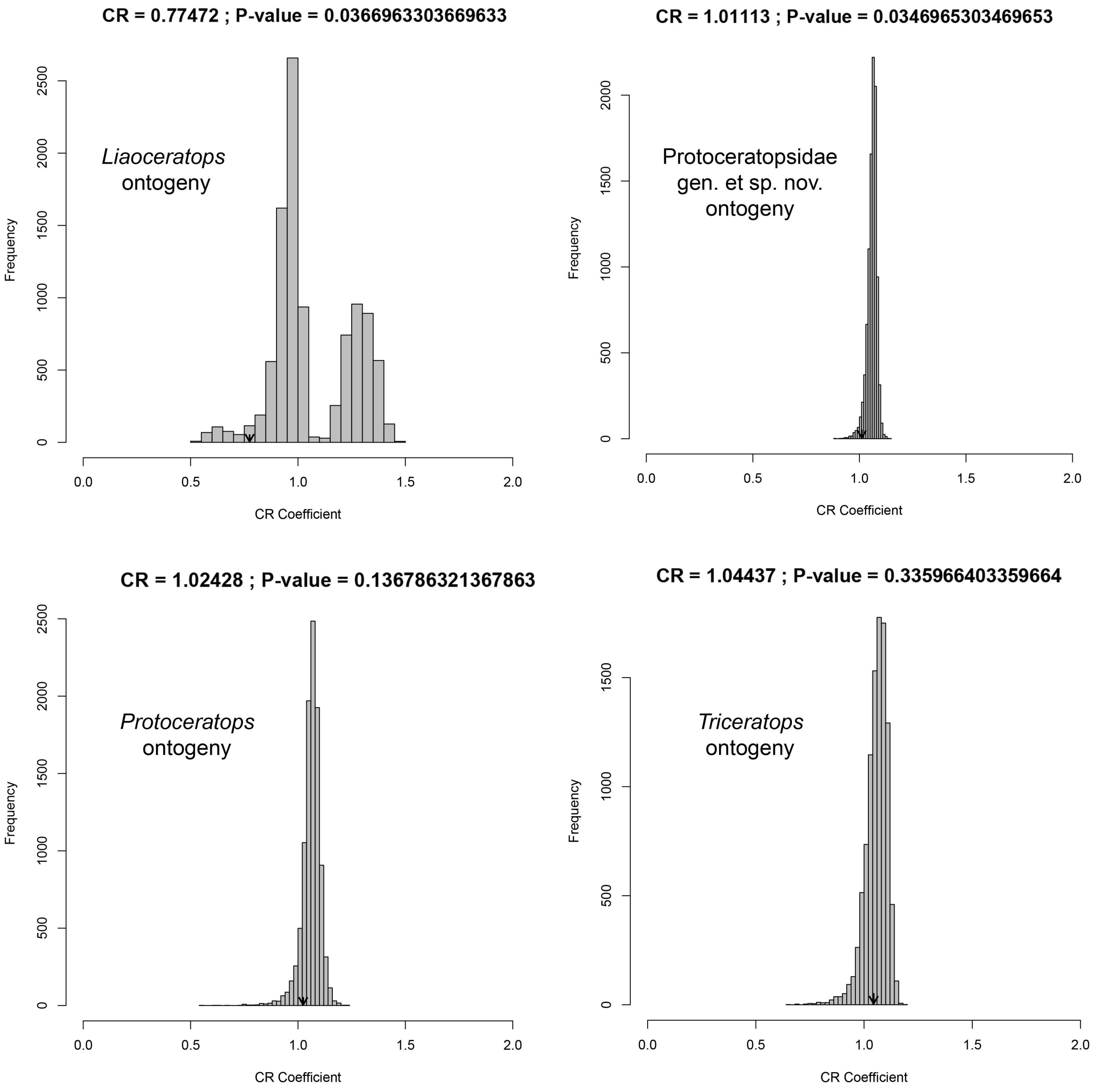

CR = 0.77472 ; P-value = 0.0366963303669633
Liaoceratops ontogeny
Frequency
CR Coefficient
0 500 1000 1500 2000 2500
0.0 0.5 1.0 1.5 2.0
CR = 1.01113 ; P-value = 0.0346965303469653
Protoceratopsidae gen. et sp. nov. ontogeny
Frequency
CR Coefficient
0 500 1000 1500 2000
0.0 0.5 1.0 1.5 2.0
CR = 1.02428 ; P-value = 0.136786321367863
Protoceratops ontogeny
Frequency
CR Coefficient
0 500 1000 1500 2000 2500
0.0 0.5 1.0 1.5 2.0
CR = 1.04437 ; P-value = 0.335966403359664
Triceratops ontogeny
Frequency
CR Coefficient
0 500 1000 1500
0.0 0.5 1.0 1.5 2.0
