# Supplementary material for: Modularity and heterochrony in the evolution of the ceratopsian dinosaur frill
Source: Ecol Evol. 2020 May 22;10(13):6288–309. doi: 10.1002/ece3.6361 (PMC7381594; doi:10.1002/ece3.6361)
Supplement: Supplementary file 11 — Appendix S11 [file ECE3-10-6288-s011.pdf]

Appendix 11. Values for various metrics resulting from the analysis of evolutionary rate of shape change.

Regression Score (RS) difference	Branch length (BL) in millions of years	RS/BL (evolutionary rate of shape change)	Taxon
0,002282	15,6	0,000146282	Psittacosauridae
0,00173697	11,375	0,000152701	Diabloceratops
0,00619907	11,375	0,000544973	Centrosaurinae
0,012632611	18,6225	0,000678352	New protoceratopsid
0,0083721	12	0,000697675	(Nasutoc. Centros., Styracos., Einios., Achelous., Pachyrhinos.)
0,00050267	0,625	0,000804272	Centros., Styracos
0,02941104	30,475	0,000965087	Coronosauria
0,000794704	0,791667	0,001003836	Kosmoceratops
0,015013561	14,6	0,001028326	Psittaco. Lujiatunesis
0,026547286	22,6	0,001174659	Psittaco. Mongoliensis
0,02475685	18,6225	0,001329405	Protoceratopsidae
0,04089499	23,375	0,001749518	Chasmosaurinae
0,00142999	0,791667	0,001806302	(Kosmoc., Arrhinoc., Treceratopsini)
0,02609985	11,375	0,002294492	Ceratopsidae
0,006957333	2,5	0,002782933	Arrhinoceratops
0,00925004	3,291667	0,002810138	(Arrhinoc., Treceratopsini)
0,001433776	0,4	0,00358444	Achelousaurus
0,007523667	1,75	0,004299238	Mojoceratops
0,00749039	1,65	0,00453963	(Einios.,Achelous., Pachyrhinos.)
0,013357866	2,875	0,004646214	Nasutoceratops
0,012313235	2,625	0,004690756	Styracosaurus
0,00333993	0,625	0,005343888	(Centros., Styracos., Einios.,Achelous., Pachyrhinos.)
0,003434383	0,625	0,005495013	Centros.
0,02237228	4	0,00559307	Triceratopsini
0,12124224	18,6225	0,006510524	Protoceratops
0,005868	0,791667	0,007412207	Utahceratops
0,175649586	19,1	0,009196313	Liaoceratops
0,0101614	1	0,0101614	(Yinlong, Psittacosauridae)
0,19408276	19,1	0,010161401	Neoceratopsia
0,010307731	1	0,010307731	Yinlong
0,037953455	3,2	0,011860455	Triceratops
0,05620362	4,2	0,013381814	Pachyrhinosaurus
0,0115482	0,791667	0,014587194	(Utahc., Pentac.)
0,0125848	0,791667	0,015896583	(Utahc., Pentac., Kosmoc., Arrhinoc., Triceratopsini)
0,0067865	0,4	0,01696625	(Achelou., Pachyrhinos.)

0,026180295	1,5	0,01745353	Regaliceratops
0,01102882	0,625	0,017646112	(Vagac., Mojoc., Chasmos.)
0,034409908	1,6	0,021506193	Einiosaurus
0,01371585	0,625	0,02194536	(Chasmosaurus+Vagaceratops)
0,018640206	0,791667	0,023545513	Pentaceratops
0,09293322	2,625	0,035403131	Vagaceratops
0,035842801	0,625	0,057348482	Chasmosaurus
3th quartile cut-off for RS/BL values		0,005017322	
4th quartile cut-off for RS/BL values		0,013001474	
