# Supplementary material for: Modularity and heterochrony in the evolution of the ceratopsian dinosaur frill
Source: Ecol Evol. 2020 May 22;10(13):6288–309. doi: 10.1002/ece3.6361 (PMC7381594; doi:10.1002/ece3.6361)
Supplement: Supplementary file 12 — Appendix S12 [file ECE3-10-6288-s012.pdf]

Appendix 12. Results of comparisons between observed vs. idealized models of evolution

Model	Parameter setting		log marginal LH	Log Bayes factor
random walk			21,552808	–
directional trend			11,926241	-19,253134
kappa	estimate (0.465)		22,030571	–
		0	22,585676	1,11021
		1	21,727032	-0,607078
lambda	estimate (0.8849)		23,427753	–
		0	-2,660838	-52,177182
		1	21,768573	-3,31836
delta	estimate (1.916)		22,175874	–
		1	21,583259	-1,18523
constant rate			8,428644	3,737996
varable rates			10,297642	–
